# Synthesis of 1,2,3-Triazole-Containing Methoxylated Cinnamides and Their Antileishmanial Activity against the *Leishmania braziliensis* Species

**DOI:** 10.3390/ph16081113

**Published:** 2023-08-07

**Authors:** Fabíola Suelen dos Santos, Rossimiriam Pereira de Freitas, Camila Simões de Freitas, Débora Vasconcelos Costa Mendonça, Daniela Pagliara Lage, Grasiele de Sousa Vieira Tavares, Amanda Sanchez Machado, Vivian Tamieti Martins, Adilson Vidal Costa, Vagner Tebaldi de Queiroz, Mariana Belizario de Oliveira, Fabrício Marques de Oliveira, Luciana Maria Ribeiro Antinarelli, Elaine Soares Coimbra, Eduardo Jorge Pilau, Geovane Perez da Silva, Eduardo Antonio Ferraz Coelho, Róbson Ricardo Teixeira

**Affiliations:** 1 Laboratório de Síntese Orgânica (LABSINTO), Departamento de Química, Universidade Federal de Minas Gerais, Belo Horizonte 31270-901, Minas Gerais, Brazil; fabiolasuelen92@hotmail.com (F.S.d.S.); rossipfreitas@gmail.com (R.P.d.F.); 2 Programa de Pós-Graduação em Ciências da Saúde: Infectologia e Medicina Tropical, Faculdade de Medicina, Universidade Federal de Minas Gerais, Belo Horizonte 31270-901, Minas Gerais, Brazil; camilasimoesf@gmail.com (C.S.d.F.); debs.mendonca@gmail.com (D.V.C.M.); danipagliara@hotmail.com (D.P.L.); grasysv@hotmail.com (G.d.S.V.T.); manda_sanchez92@hotmail.com (A.S.M.); viviantamietti@yahoo.com.br (V.T.M.); eduardoferrazcoelho@yahoo.com.br (E.A.F.C.); 3 Grupo de Estudo Aplicado em Produtos Naturais e Síntese Orgânica (GEAPS), Departamento de Química e Física, Universidade Federal do Espírito Santo, Alegre 29500-000, Espírito Santo, Brazil; avcosta@hotmail.com (A.V.C.); vagner.queiroz@ufes.br (V.T.d.Q.); mariana.b.oliveira@edu.ufes.br (M.B.d.O.); 4 Instituto Federal de Minas Gerais (IFMG), Campus Ouro Branco, Ouro Branco 36420-000, Minas Gerais, Brazil; fabricio.marques@ifmg.edu.br; 5 Departamento de Parasitologia, Microbiologia e Imunologia, Instituto de Ciências Biológicas, Universidade Federal de Juiz de Fora, Juiz de Fora 36036-900, Minas Gerais, Brazil; lucianantinarelli@yahoo.com.br (L.M.R.A.); elaine.coimbra@ufjf.edu.br (E.S.C.); 6 Centro de Ciências Exatas, Departamento de Química, Universidade Estadual de Maringá, Maringá 87020-900, Paraná, Brazil; ejpilau@uem.br (E.J.P.); geovane.perez13@gmail.com (G.P.d.S.); 7 Grupo de Síntese e Pesquisa de Compostos Bioativos (GSPCB), Departamento de Química, Universidade Federal de Viçosa, Viçosa 36570-900, Minas Gerais, Brazil

**Keywords:** tegumentary leishmaniasis, *Leishmania braziliensis*, click chemistry, cinnamic acid derivatives, CuAAC

## Abstract

Leishmaniasis is a group of infectious diseases caused by protozoan parasites that belong to the genus *Leishmania*. Currently, there is no human vaccine, and the available treatments are associated with toxicity, high cost, and the emergence of resistant strains. These factors highlight the need to identify new antileishmanial candidates. In this study, we synthesized twenty-four methoxylated cinnamides containing 1,2,3-triazole fragments and evaluated their antileishmanial activity against the *Leishmania braziliensis* species, which is the main etiological agent responsible for American Tegumentary Leishmaniasis (ATL). The cinnamides were synthetically prepared using nucleophilic acyl substitution and copper(I)-catalyzed azide–alkyne cycloaddition (CuAAC) reactions. The compounds were characterized using infrared, nuclear magnetic resonance, and high-resolution mass spectrometry techniques. We performed preliminary studies to evaluate the biological activity of these compounds against *L. braziliensis* promastigotes and axenic amastigotes. Compound **28**, *N*-((1-(7-(diethylamino)-2-oxo-2*H*-chromen-3-yl)-1*H*-1,2,3-triazole-4-yl) methyl)-3,4-dimethoxy cinnamide, demonstrated relevant antileishmanial activity with low toxicity in murine cells. The selectivity index values for this compound were superior compared with data obtained using amphotericin B. Furthermore, this cinnamide derivative reduced the infection percentage and number of recovered amastigotes in *L. braziliensis*-infected macrophages. It also induced an increase in reactive oxygen species production, depolarization of the mitochondrial potential, and disruption of the parasite membrane. Taken together, these findings suggest that this synthetic compound holds potential as an antileishmanial candidate and should be considered for future studies in the treatment of ATL.

## 1. Introduction

Leishmaniasis consists of a group of neglected tropical diseases caused by parasites that belong to the genus *Leishmania* [1]. This disease affects approximately 380 million people in 98 countries, with an estimated global incidence of 0.9 to 1.6 million cases per year [2,3]. The parasites are transmitted to mammals, including humans and dogs, through the bite of an infected female phlebotomine sandfly [4]. The majority of leishmaniasis cases occur in impoverished populations who often face malnutrition and have compromised immune systems. Other contributing factors include inadequate housing, limited financial resources, population displacement, and environmental changes [3].

**Figure 1:**
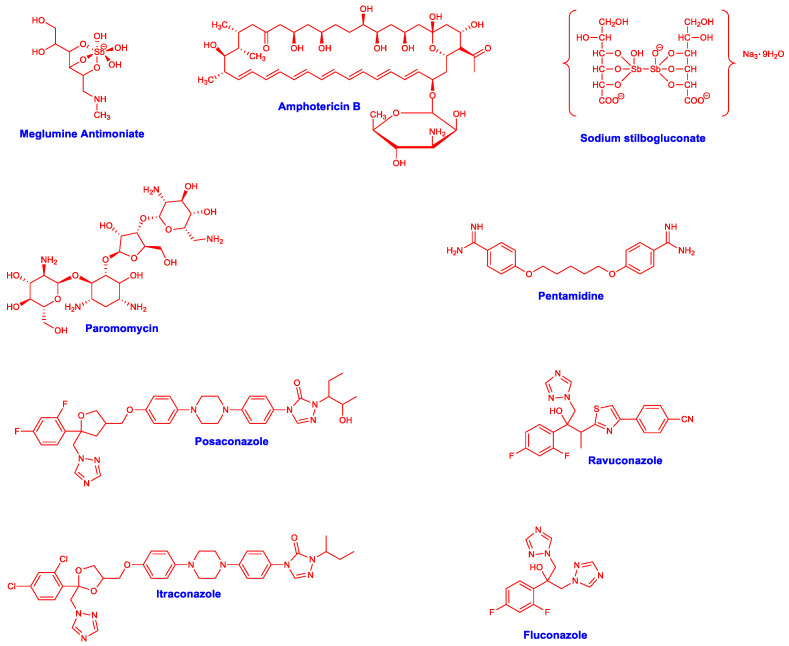
Structures of compounds with leishmanicidal effects.

The clinical manifestations of this disease consist of tegumentary (TL) and visceral leishmaniasis (VL) [5,6], with VL being the most severe form of the disease, as it can be fatal if left untreated. Currently, there is no human vaccine against leishmaniasis, and the currently available treatments are associated with toxicity, high cost, and the emergence of resistant strains [7]. These antileishmanial agents (Figure 1) include pentavalent antimonials (meglumine antimoniate and sodium stibogluconate), pentamidine, miltefosine, liposomal and free amphotericin B (AmpB), and paromomycin, all of which can cause toxicity, teratogenicity, and/or present a high cost [8,9,10]. Therefore, there is a pressing need to identify new compounds with selective action against *Leishmania*. Within this context, compounds presenting triazole functionalities, some of them shown in Figure 1, have been identified with relevant leishmanicidal effects [11,12,13,14].

Natural products are a valuable resource when exploring therapeutic agents, including for the treatment of leishmaniasis [15]. They can be used directly as medicinal agents or chemically modified to improve their biological activity. In this regard, cinnamic acid is an attractive compound found in several plant species, with a long history of use as human medicine for the treatment of various diseases [16,17,18]. Studies have demonstrated the biological activity of cinnamic acid derivatives and 1,2,3-triazole-based compounds, including their leishmanicidal effect [18,19,20,21,22,23,24]. However, few studies have assessed the antileishmanial activity of these compounds against the *Leishmania braziliensis* species, which is considered the main etiological agent of TL in the Americas.

**Figure 2:**
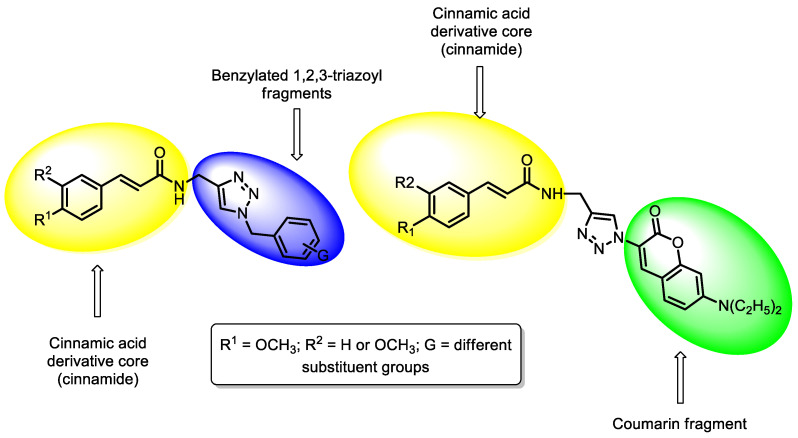
Design of the compounds investigated in this study.

In this study, a series of twenty-four methoxylated cinnamides containing 1,2,3-triazole fragments were synthesized. Previous investigations conducted by our research group have identified various bioactive compounds containing benzyl groups with different substitution patterns, linked to 1,2,3-triazolyl moieties [20,21,22,23,24,25,26]. Additionally, the combination of cinnamic acid with benzylated 1,2,3-triazolyl fragments resulted in compounds with significant antileishmanial effect on *L. braziliensis* [27]. Consequently, we decided to incorporate this structural motif (highlighted in blue in Figure 2) into the core of cinnamides (highlighted in yellow in Figure 2). Moreover, we planned to introduce coumarin fragments (highlighted in green in Figure 2) into the structure of the cinnamides, since coumarins are privileged scaffolds known for their diverse bioactivities, including antileishmanial action [28,29,30]. The connection between the cinnamide core and the coumarin fragment was planned via a 1,2,3-triazoyl group.

The synthesized compounds had their antileishmanial activity evaluated in vitro against *L. braziliensis* promastigotes and amastigotes. Additionally, their toxicity on mammalian cells, treatment of infected macrophages, and preliminary assay on the mechanism of action of the most promising synthetic compound, Compound **28**, were conducted. These results indicated that Compound **28** demonstrates superior and more selective action against parasites compared with the data obtained using AmpB. 

## 2. Results

### 2.1. Synthesis of Chemical Derivatives

**Scheme 1:**
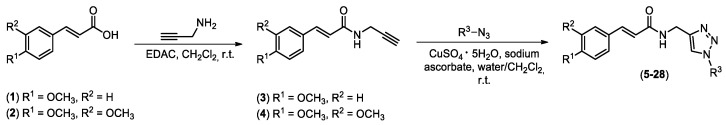
Synthetic steps involved in the preparation of cinnamides **5**–**28**.

The steps involved in the preparation of the twenty-four cinnamides containing 1,2,3-triazole functionalities, i.e., Compounds **5**–**28**, are shown in Scheme 1. The first step corresponds to the preparation of Compounds **3** and **4** via a nucleophilic acyl substitution reaction between the carboxylic acids **1** and **2** and the propargyl amine in the presence of EDAC [31,32]. Thereafter, amides **3** and **4** were reacted with different organic azides via copper(I)-catalyzed azide–alkyne cycloaddition (CuAAC) click reactions [33,34,35,36] which generated the cinnamides **5**–**28** in yields varying from 52–91% (Table 1). The azides used for the preparation of Compounds **5**–**28** were obtained from previously described methodologies [37,38].

The compounds were characterized by nuclear magnetic resonance (NMR) (^1^H and ^13^C) and infrared (IR) spectroscopic techniques as well as high-resolution mass spectrometry analysis. In the IR spectra, N-H stretching was observed within the 3218–3354 cm^−1^ range. The chemical shifts for the carbons of the carbonyl groups of the amide functionality were noticed at ~165 ppm. The signals for the hydrogens of the triazole fragment were found as singlets within the 7.95–8.44 ppm range. The molecular formulas of the cinnamides were confirmed based on HRMS analysis. The spectra that support the structures of the twenty-four 1,2,3-triazole-containing methoxylated cinnamides are presented in the Supplementary Material (Figures S1–S96).

### 2.2. Antileishmanial Activity and Cytotoxicity on Mammalian Cells

An initial screening was performed to evaluate the in vitro antileishmanial activity of the twenty-four 1,2,3-triazole-containing methoxylated cinnamides against *L. braziliensis* promastigotes and axenic amastigotes. The results indicated that Compounds **5**–**27** presented with low antileishmanial activity, with IC_50_ values greater than 500 µg/mL against both parasite stages, and they were therefore not considered for additional studies. Compound **28** showed IC_50_ values of 105.7 ± 16.7 and 87.97 ± 9.7 µg/mL against promastigotes and amastigotes, respectively (Table 2). Its cytotoxicity, which is indicated by the CC_50_ value, was 1169.0 ± 113.2 µg/mL. The IC_50_ results for AmpB were 0.974 ± 0.2 and 1.30 ± 0.4 µg/mL against the promastigotes and amastigotes, respectively, and the CC_50_ value was 8.90 ± 0.8 µg/mL (Table 2).

The selectivity index (SI) was calculated as the ratio between the CC_50_ and IC_50_ values, and the results for Compounds **28** and AmpB were 11.1 and 9.1 against parasite promastigotes and 13.3 and 6.8 against axenic amastigotes, respectively, suggesting a lower cytotoxicity of Compound **28** against mammalian cells compared with AmpB. This is an important advantage considering that AmpB is an extremely toxic drug.

### 2.3. Treatment of Infected Macrophages and Inhibition of Infection

The treatment of infected macrophages was evaluated to investigate the potential in vitro therapeutic action against *L. braziliensis* in mammalian cells. The results showed that Compound **28** presented satisfactory antileishmanial action, with a considerable reduction in the parasite load and in the number of recovered amastigotes being noted. In this context, using 5.0 µg/mL of this compound, the infection percentage was 60.2% and the number of amastigotes per cell was 2.9 (Table 3). Using 5.0 µg/mL of AmpB, the reduction in the parasite load was higher than that by Compound **28**; however, it is relevant to consider that, as indicated by the CC_50_ values, the drug is more toxic to mammalian cells, thus raising the possibility of testing our compound in delivery systems to optimize the antileishmanial activity so as to still be detrimental at possibly a lower concentration. Infected and untreated murine cells presented an infection degree of 92.8%, and the number of recovered amastigotes was 10.3. To evaluate the inhibitory action of Compound **28** against *Leishmania* infection, which would suggest a preventive/prophylactic role, parasites were first incubated with the molecule and later used to infect mammalian cells. The results showed that promastigotes that were pre-incubated with 5.0 µg/mL of Compound **28** presented an infection percentage of 71.5%, and the number of amastigotes per cell was 3.6 (Table 3). Parasites not incubated with the molecule caused an infection percentage of 90.5% and a number of recovered amastigotes of 8.9, suggesting a preventive/prophylactic action of Compound **28** against a later infection. 

### 2.4. Preliminary Evaluation of the Mechanism of Action on Leishmania

**Figure 3:**
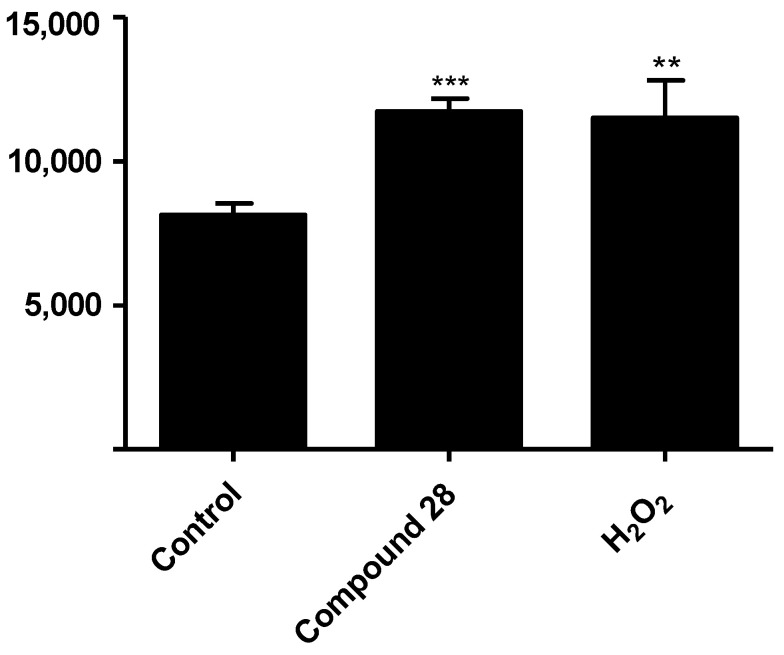
**Production of ROS in *L. braziliensis*-infected macrophages treated with Compound 28**. *Leishmania braziliensis*-infected macrophages (1 × 10^5^ cells) were incubated with Compound **28** (5.0 µg/mL) for 48 h at 37 °C in a 5% CO_2_ environment. Subsequently, 2′,7′-dichlorodihydrofluorescein diacetate (H_2_DCFDA, 9.7 μg/mL) was added for 30 min under dark conditions. The fluorescence levels were measured in a spectrofluorometer at 485/528 nm. H_2_O_2_ (0.5 µmol/L) was used as a positive control. Statistical significance is denoted by (**) and (***), indicating significant differences in comparison with the control (*p* < 0.01 and *p* < 0.001, respectively).

To perform a preliminary analysis of the mechanism of action of Compound **28** in *L. braziliensis*-infected macrophages, reactive oxygen species (ROS) production was evaluated. The results show that treatment using Compound **28** at 5.0 µg/mL increased the ROS levels in the treated and infected cells by 44.1% compared with data obtained from the untreated cells. As a positive control, H_2_O_2_-treated and infected cells showed a 41.4% increase in ROS production (Figure 3).

### 2.5. Evaluation of Leishmania Targets by Compound ***28***

**Figure 4:**
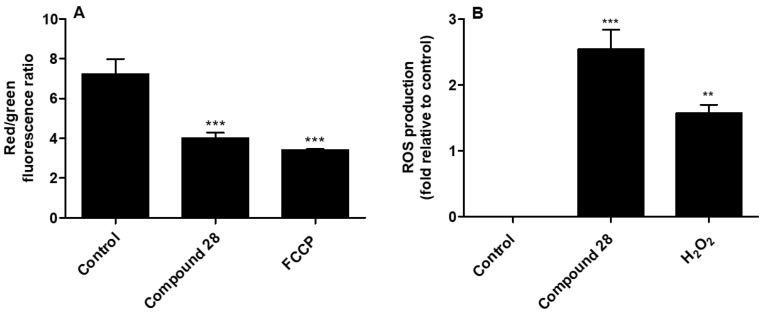
**ΔΨm and ROS production in *Leishmania* after exposure to Compound 28.** *Leishmania braziliensis* promastigotes were not exposed (negative control) or exposed to Compound **28** (at a concentration corresponding to the IC_50_ value) for 48 h. (**A**) Depolarization of ΔΨm was evaluated using the JC-1 fluorescent probe. Samples were analyzed fluorometrically, and the results are expressed as the ratio of red/green fluorescence. FCCP (0.02 μg/mL) was used as a positive control. (**B**) ROS production was evaluated using the H_2_DCFDA probe. The fluorescence intensity was measured by fluorimetry, and the results are expressed as the fold increase in the ROS level compared with the non-treated control. H_2_O_2_-treated parasites were used as a positive control. In both assays, three independent experiments were performed. (**) and (***) indicate statistically significant differences compared with the non-treated control (*p* < 0.01 and *p* < 0.001, respectively).

The mitochondrial membrane potential (ΔΨm) was evaluated in the treated parasites, and the results showed a significant reduction in parasites treated with Compound **28**, similar to those that received carbonyl cyanide-*p*-trifluoromethoxyphenylhydrazone (FCCP) treatment as a positive control (Figure 4A). The untreated parasites presented with a higher ΔΨm value. Based on the effect of Compound **28** causing a reduction of the ΔΨm, ROS levels were also evaluated, and the results showed that Compound **28** promoted a strong 2.5-fold increase in ROS production compared with the untreated parasites. H_2_O_2_-incubated promastigotes exhibited a significant 1.6-fold increase in ROS levels when compared with the untreated parasites (Figure 4B).

**Figure 5:**
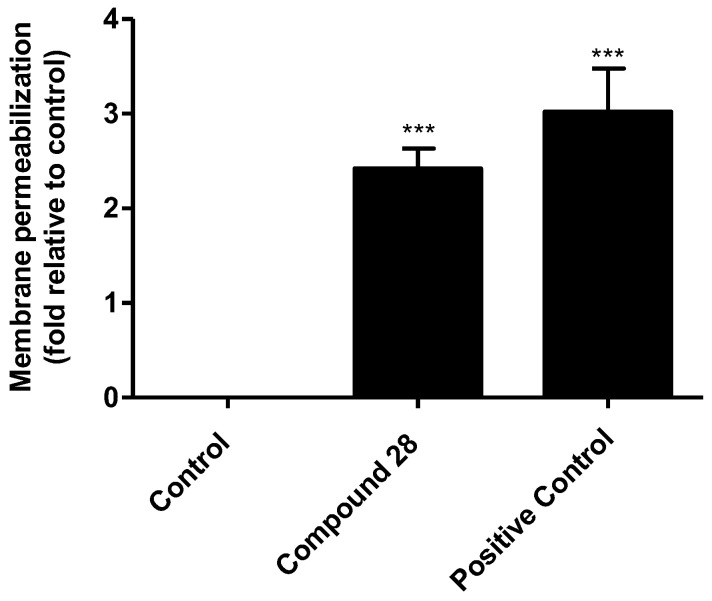
**Effect of Compound 28 on parasite membrane permeability.** Parasite promastigotes were either left untreated or treated with Compound **28** (at a concentration corresponding to the IC_50_ value) for 48 h. The PI dye fluorescence was measured fluorometrically at excitation and emission wavelengths of 540 and 600 nm, respectively. Parasites heated at 65 °C for 10 min were used as a positive control. The data is presented as the fold increase in PI fluorescence relative to the untreated control. (***), signifying *p* < 0.001, indicates a statistically significant difference compared with the non-treated control.

To evaluate the extent of damage to the *Leishmania* membrane caused by Compound **28**, staining with propidium iodide (PI) was performed, and the results showed a significant 2.3-fold increase in PI-fluorescence after treatment with Compound **28** compared with values obtained using untreated parasites. Like the positive control, heat-killed parasites presented a 3.0-fold increase in the plasma membrane permeabilization (Figure 5).

## 3. Discussion

In the present study, a series of twenty-four 1,2,3-triazole-containing methoxylated cinnamides were synthesized and tested against *L. braziliensis*, the main *Leishmania* species responsible for American TL. The leishmanicidal effect of amides derived from cinnamic acid remains relatively unexplored [39,40,41]. Therefore, in this study, we aimed to investigate the potential of 4-methoxylated and 3,4-dimethoxylated cinnamides as a scaffold (depicted in yellow in Figure 2). 

Once prepared, Compounds **5**–**28** were assessed using bioassays to evaluate their antileishmanial activity. Results showed that Compound **28** presented with satisfactory antileishmanial activity and low toxicity in mammalian cells, showing a satisfactory SI that was higher than that found for AmpB, a known antileishmanial drug [42,43].

The screening of antileishmanial candidates is usually performed on parasite promastigote cultures, primarily due to the ease of cultivating the parasites and the resulting yield [44,45,46]. However, evaluations using amastigotes should also be performed, since this is the parasite stage that directly interacts with the host immune system and is responsible for the active disease [47]. Compound **28** exhibited superior in vitro efficacy against axenic amastigotes compared with the promastigotes. Moreover, the treatment of infected macrophages was assessed to investigate the potential in vitro therapeutic efficacy of this compound against *L. braziliensis* in mammalian cells. The results demonstrated that the Compound **28** also exhibited satisfactory action when compared with the data obtained from the use of AmpB. A significant reduction in the parasite load was observed in treated and infected macrophages as well as in the number of recovered amastigotes per treated cell.

Considering the reduction in the number of amastigotes after the treatment of infected macrophages as an in vitro parameter for the antileishmanial success of the candidate compounds, we investigated whether the treatment of infected cells with Compound **28** could reduce the infection percentage and parasite load in the treated cells. The results showed that the cinnamide **28** presented good antileishmanial activity against infection, with significant reductions in the percentage and number of recovered amastigotes when compared with data obtained using untreated cells. Furthermore, this 1,2,3-triazole derivative also exhibited potential as a preventive/prophylactic agent in future studies. Pre-incubation of parasites with Compound **28** reduced the infection percentage and the number of recovered amastigotes, indicating the possibility of employing this derivative as a preventive agent in additional in vivo investigations.

ROS are highly reactive biomolecules that can eliminate intracellular parasites by inducing oxidative mechanisms in the host cells [48,49]. Therefore, to investigate whether Compound **28** induced the upregulation of these oxidative functions in infected macrophages, the accumulation of ROS in the infected macrophages treated with Compound **28** was measured. Although an immunological evaluation of the culture supernatant after treatment was not performed, it can be inferred that the reduction in the presence of intracellular parasites upon treatment may be attributed to the production of anti-parasitic effector molecules, as previously described [50,51]. This suggests that cell activation through pro-inflammatory cytokines, nitric oxide, and oxidative mediators may kill the parasites without causing toxicity in these mammalian cells. The presented results collectively suggest that treatment with Compound **28**, either before or after infection, impairs *L. braziliensis* macrophage infection, possibly via oxidative stress mechanisms, including the induction of ROS as well as reactive nitrogen species (RNS). However, additional studies are required to further investigate this mechanism.

Considering the potential antileishmanial effect of Compound **28**, we investigated cellular targets which may be involved in parasite death and focused our studies on the evaluation of mitochondrial function and plasma membrane integrity. The mitochondria of *Leishmania* parasites are functionally distinct from those in mammalian cells in terms of their bioenergetics and oxidative metabolism, biosynthetic pathway, antioxidant enzymes, and mitochondrial DNA compartmentalization in the kinetoplastid structure. Given its essential role in parasite survival, impaired mitochondrial function is critical for the activation of the cell death machinery in *Leishmania* parasites. Therefore, this organelle serves as a selective target to identify effective antileishmanial compounds [52,53,54,55].

The analysis of ΔΨm depolarization is a key indicator to assess mitochondrial dysfunction [55]. The ΔΨm assay was performed using the cationic fluorescent dye JC-1, which selectively accumulates in viable mitochondria as aggregates and exhibits red fluorescence. In collapsed mitochondria with low membrane potential, JC-1 remains as a monomer in the cytoplasm and emits green fluorescence. The results were expressed as a ratio of red/green fluorescence, which showed that the JC-1 ratio was significantly reduced to 4.0 in promastigotes of *L. braziliensis* treated with Compound **28**, while it was 7.2. in the non-treated parasites. In the FCCP-treated parasites, the ratio was reduced to 3.4.

Similar to mammalian cells, the mitochondrial respiratory chain of trypanosomatids is the primary site of ROS production. Therefore, the depolarization of the mitochondrial membrane potential leads to respiratory chain breakdown, resulting in the overproduction of ROS. Excess ROS can cause irreversible damage to cell structures, including lipid peroxidation, protein oxidation, and DNA damage, thus compromising parasite survival [56,57]. Based on the effect of Compound **28** on the ΔΨm reduction, the ROS level in promastigote forms was also evaluated during the incubation period. Our data demonstrated that Compound **28** significantly increased ROS production by 2.5-fold compared with the non-treated control. In addition, promastigotes incubated with H_2_O_2_ exhibited a significant 1.6-fold increase in ROS levels compared with the non-treated control (Figure 3B).

Targeting the plasma membrane is a promising approach in the search for new therapeutic candidates against leishmaniasis. In mammalian cells, cholesterol is a key sterol in the cell membrane, while trypanosomatids contain ergosterol and other 24-methyl sterols that are essential for their growth and viability [58,59]. Current drugs used to treat leishmaniasis are known to target the plasma membrane of the parasite. For example, AmpB binds to ergosterol, which causes pore formation and membrane disruption [60]. Similarly, miltefosine inhibits lipid biosynthesis [61]. Membrane disruption can have various effects on the plasma membrane, such as interfering with the transport of metabolites and ionic gradients, impeding the acquisition of essential nutrients, and altering membrane fluidity. These disruptions ultimately lead to parasite death through osmotic lysis [62,63,64]. To assess the extent of damage caused by Compound **28** to the promastigote plasma membrane, PI staining was performed. PI is impermeable to live cell membranes; therefore, this dye does not cross intact membranes. However, if the cell plasma membrane is not intact, this probe can cross the nuclear membrane and bind to nucleic acids. The results revealed a significant 2.3-fold increase in PI fluorescence after treatment with Compound **28** compared with the non-treated parasites. This finding indicates that this cinnamide derivative alters the integrity of the plasma membrane, which is primarily associated with necrosis, as the rupture of the plasma membrane is an event closely linked to this process. In our previous work, we conducted ultrastructural analysis on a series of cinnamic acid derivatives containing isobenzofuranone functionality and found that they caused a loss of cytoplasmic content in *L. braziliensis* parasites. This finding is indicative of plasma membrane rupture, providing further support for the results presented here [27]. Compound **28**, in particular, possesses two methoxy groups and a triazole fragment bond with coumarin functionality, which potentially explains its selective action against parasites. 

## 4. Materials and Methods

### 4.1. Chemicals and General Information

The solvents used in this study were purchased from Vetec (Rio de Janeiro, RJ, Brazil), Sigma-Aldrich (St. Louis, MO, USA), and Synth (Diadema, São Paulo, Brazil) and were distilled before use. Distilled water was utilized in the experiments. Other reagents were procured from Vetec, Sigma-Aldrich, Synth, and Oakwood Chemical (Estill, South Carolina, USA) and used without further purification. The progress of the reactions was monitored by thin-layer chromatography (TLC). For the purification of the reaction products, silica-gel column chromatography was employed (SiliCycle 0.035–0.070 mm, pore diameter 6 nm). The NMR spectra were recorded on Bruker (Billerica, Massachusetts, USA) AVANCE DPX 200 MHz and AVANCE-III Onebay, and Nanobay 400 MHz instruments, using DMSO-*d_6_* as a deuterated solvent. The ^1^H NMR data are presented as follows: chemical shift (δ) in ppm, multiplicity, number of hydrogens, and *J* values in Hz. Multiplicities are indicated by the following abbreviations: s (singlet), d (doublet), dd (doublet of doublet), sept (septet), t (triplet), m (multiplet), and q (quartet). For fluorine-containing derivatives, the multiplicity of some carbon signals is described, along with *J* values in Hz. The IR spectra were obtained using Varian 660-IR (Palo Alto, CL, USA) equipped with GladiATR scanning from 4000–500 cm^−1^. The melting points were determined using an MQAPF-302 melting point apparatus (Microquímica, Santa Catarina, Brazil) and are uncorrected. 

### 4.2. Synthesis of Compounds ***5**–**28*** Exemplified through the Synthesis of N-((1-(4-methyl benzyl)-1H-1,2,3-triazol-4-yl)methyl) (4-methoxy) Cinnamide (***5***)

In a 10 mL round-bottom flask, azide (0.116 g, 0.79 mmol), water (3.00 mL), dichloromethane (3.00 mL), sodium ascorbate (0.063 g, 0.32 mmol), *N*-(prop-2-yn-1-yl) (4-methoxy) cinnamide (**3**) (0.170 g, 0.79 mmol), and CuSO_4_·5H_2_O (0.0400 g, 0.160 mmol) were added. The mixture was vigorously stirred for 30 min at room temperature. Subsequently, water (10 mL) was added, and the resulting aqueous phase was extracted with dichloromethane (3 × 20 mL). The organic extracts were combined, and the resulting organic phase was dried over anhydrous Na_2_SO_4_, filtered, and concentrated under reduced pressure. Compound **5** was purified from the residue by silica-gel column chromatography and eluted with hexane/ethyl acetate/methanol (5:3:1, *v*/*v*). The described procedure produced Compound **5** with an 84% yield (0.241 g, 0.660 mmol) and the following characteristics: White solid, m.p. 166.0–167.2 °C. IR (ATR) ${\bar{v}}_{max}$ (cm^−1^): 3252, 1600, 1511, 1215, 1028, 822. ^1^H NMR (400 MHz, DMSO-*d_6_*) δ: 2.27 (s, 3H), 3.77 (s, 3H), 4.40 (d, 2H, *J* = 5.6 Hz), 5.50 (s, 2H), 6.49 (d, 1H, *J* = 15.8 Hz), 6.96 (d, 2H, *J* = 8.7 Hz), 7.16 (d, 2H, *J* = 8.0 Hz), 7.22 (d, 2H, *J* = 8.0 Hz), 7.39 (d, 1H, *J* = 15.8 Hz), 7.49 (d, 2H, *J* = 8.7 Hz), 7.95 (s, 1H), 8.49 (t, 1H, *J* = 5.6 Hz). ^13^C NMR (100 MHz, DMSO-*d_6_*) δ: 20.7, 34.4, 52.6, 55.3, 114.4, 119.4, 122.9, 127.4, 128.1, 129.2, 129.3, 133.1, 137.6, 138.8, 145.1, 160.4, 165.3. HRMS (ESI^+^) calcd. for [C_21_H_22_N_4_O_2_Na]^+^ [M + Na]^+^: 385.1641; found: 385.1616. 

Compounds **6**–**27** were prepared from the corresponding alkyne and azides as described for Compound **5**. The structures of the compounds are supported by the following data.

*N*-((1-(4-isopropyl benzyl)-1*H*-1,2,3-triazol-4-yl) methyl) (4-methoxy) cinnamide (**6**):

White solid, obtained at 67% yield, m.p. 187.4–188.3 °C. IR (ATR) ${\bar{v}}_{max}$ (cm^−1^): 3275, 2955, 1604, 1512, 828. ^1^H NMR (400 MHz, DMSO-*d_6_*) δ: 1.16 (d, 6H, *J* = 6.9 Hz), 2.85 (sept, 1H, *J* = 6.9 Hz), 3.78 (s, 3H), 4.41 (d, 2H, *J* = 5.6 Hz), 5.51 (s, 2H), 6.49 (d, 1H, *J* = 15.8 Hz), 6.97 (d, 2H, *J* = 8.7 Hz), 7.23 (d, 2H, *J* = 8.7 Hz), 7.26 (d, 2H, *J* = 8.7 Hz), 7.40 (d, 1H, *J* = 15.8 Hz), 7.50 (d, 2H, *J* = 8.7 Hz), 7.99 (s, 1H), 8.49 (t, 1H, *J* = 5.6 Hz). ^13^C NMR (100 MHz, DMSO-d_6_) δ: 23.8, 33.1, 34.3, 52.6, 55.3, 114.4, 119.4, 122.9, 126.6, 127.4, 128.1, 129.1. 133.5, 138.7, 145.1, 148.4, 160.4, 165.2. HRMS (ESI^+^) calcd. for [C_23_H_27_N_4_O_2_Na]^+^ [M + Na]^+^: 391.2134; found: 391.2107.

*N*-((1-(4-fluoro benzyl)-1*H*-1,2,3-triazol-4-yl) methyl) (4-methoxy) cinnamide (**7**): 

White solid, obtained at 90% yield, m.p. 178.6–180.0 °C. IR(ATR) ${\bar{v}}_{max}$ (cm^−1^): 3284, 1600, 1508, 1214, 824. ^1^H NMR (400 MHz, DMSO-*d_6_*) δ: 3.77 (s, 3H), 4.41 (d, 2H, *J* = 5.4 Hz), 5.56 (s, 2H), 6.50 (d, 1H, *J* = 15.7 Hz), 6.96 (d, 2H, *J* = 8.4 Hz), 7.20 (t, 2H, *J* = 8,6 Hz), 7.38–7.42 (m, 3H), 7.49 (d, 2H, *J* = 8.4 Hz), 8.02 (s, 1H), 8.49 (t, 1H, *J* = 5.4 Hz). ^13^C NMR (100 MHz, DMSO-*d_6_*) δ: 34.3, 51.9, 55.2, 114.4, 115.6 (d, *J* = 22 Hz), 119.4, 123.0, 127.4, 129.1, 130.3 (d, *J* = 8.5 Hz), 132.4 (d, *J* = 3.0 Hz), 138.7, 145.3, 160.4, 161.9 (d, *J* = 244 Hz), 165.2. HRMS (ESI^+^) calcd. for [C_20_H_20_FN_4_O_2_]^+^ [M + H]^+^: 367.1570; found: 367.1543.

*N*-((1-(4-chloro benzyl)-1*H*-1,2,3-triazol-4-yl) methyl) (4-methoxy) cinnamide (**8**): 

White solid, obtained at 75% yield, m.p. 185.7–186.5 °C. IR (ATR) ${\bar{v}}_{max}$ (cm^−1^): 3237, 1598, 1511, 1215, 827. ^1^H NMR (400 MHz, DMSO-*d_6_*) δ: 3.78 (s, 3H), 4.42 (d, 2H, *J* = 5.6 Hz), 5.57 (s, 2H), 6.50 (d, 1H, *J* = 15.8 Hz), 6.96 (d, 2H, *J* = 8.7 Hz), 7.34 (d, 2H, *J* = 8.4 Hz), 7.40 (d, 1H, *J* = 15.8 Hz), 7.44 (d, 2H, *J* = 8.0 Hz), 7.50 (d, 2H, *J* = 8.7 Hz), 8.02 (s, 1H), 8.49 (t, 1H, *J* = 5.6 Hz). ^13^C NMR (100 MHz, DMSO-*d_6_*) δ: 34.3, 51.9, 55.3, 114.4, 119.4, 123.1, 127.4, 128.8, 129.1, 130.0, 132.9, 135.1, 138.8, 145.2, 160.4, 165.2. HRMS (ESI^+^) calcd. for [C_20_H_20_ClN_4_O_2_]^+^ [M + H]^+^: 383.1275; found: 383.1255.

*N*-((1-(4-bromo benzyl)-1*H*-1,2,3-triazol-4-yl) methyl) (4-methoxy) cinnamide (**9**): 

White solid, obtained at 65% yield, m.p. 193.1–194.7 °C. IR (ATR) ${\bar{v}}_{max}$ (cm^−1^): 3243, 1599, 1511, 1214, 1012, 827. ^1^H NMR (400 MHz, DMSO-*d_6_*) δ: 3.78 (s, 3H), 4.42 (d, 2H, *J* = 5.4 Hz), 5.56 (s, 2H), 6.50 (d, 1H, *J* = 15.8 Hz), 6.97 (d, 2H, *J* = 8.5 Hz), 7.28 (d, 2H, *J* = 8.2 Hz), 7.40 (d, 1H, *J* = 15.8 Hz), 7.50 (d, 2H, *J* = 8.5 Hz), 7.57 (d, 2H, *J* = 8.2 Hz), 8.02 (s, 1H), 8.50 (t, 1H, *J* = 5.4 Hz). ^13^C NMR (100 MHz, DMSO-*d_6_*) δ: 34.3, 52.0, 55.3, 114.4, 119.4, 121.4, 123.1, 127.4, 129.1, 130.3, 131.7, 135.5, 138.7, 145.2, 160.4, 165.2. HRMS (ESI^+^) calcd. for [C_20_H_19_BrN_4_O_2_Na]^+^ [M + Na]^+^: 449.0589; found: 449.0559.

*N*-((1-(4-iodo benzyl)-1*H*-1,2,3-triazol-4-yl) methyl) (4-methoxy) cinnamide (**10**): 

White solid, obtained at 76% yield, m.p. 200.6–202.0 °C. IR (ATR) ${\bar{v}}_{max}$ (cm^−1^): 3260, 1600, 1511, 1215, 1007, 826. ^1^H NMR (400 MHz, DMSO-*d_6_*) δ: 3.78 (s, 3H), 4.41 (d, 2H, *J* = 5.6 Hz), 5.53 (s, 2H), 6.49 (d, 1H, *J* = 15.8 Hz), 6.97 (d, 2H, *J* = 8.7 Hz), 7.12 (d, 2H, *J* = 8.3 Hz), 7.40 (d, 1H, *J* = 15.8 Hz), 7.50 (d, 2H, *J* = 8.7 Hz), 7.74 (d, 2H, *J* = 8.3 Hz), 8.01 (s, 1H), 8.49 (t, 1H, *J* = 5.6 Hz). ^13^C NMR (100 MHz, DMSO-*d_6_*) δ: 34.3, 52.2, 55.3, 94.4, 114.4, 119.4, 123.1, 127.4, 129.1, 130.3, 135.9, 137.5, 138.8, 145.2, 160.4, 165.2. HRMS (ESI^+^) calcd. for [C_20_H_20_IN_4_O_2_]^+^ [M + H]^+^: 475.0631; found: 475.0605.

*N*-((1-(4-nitro benzyl)-1*H*-1,2,3-triazol-4-yl) methyl) (4-methoxy) cinnamide (**11**): 

White solid, obtained at 65% yield, m.p. 192.2–193.4 °C. IR (ATR) ${\bar{v}}_{max}$ (cm^−1^): 3236, 1590, 1514, 1214, 1024, 828. ^1^H NMR (400 MHz, DMSO-*d_6_*) δ: 3.78 (s, 3H), 4.44 (d, 2H, *J* = 5.6 Hz), 5.76 (s, 2H), 6.50 (d, 1H, *J* = 15.7 Hz), 6.97 (d, 2H, *J* = 8.7 Hz), 7.40 (d, 1H, *J* = 15.7 Hz), 7.50 (d, 2H, *J* = 8.8 Hz), 7.54 (d, 2H, *J* = 8.7 Hz), 8.10 (s, 1H), 8.23 (d, 2H, *J* = 8.8 Hz), 8.52 (t, 1H, *J* = 5.6 Hz). ^13^C NMR (100 MHz, DMSO-*d_6_*) δ: 34.3, 51.8, 55.2, 114.4, 119.3, 123.5, 123.9, 127.4, 129.08, 129.09, 138.7, 143.5, 145.3, 147.2, 160.4, 165.2. HRMS (ESI^+^) calcd. for [C_20_H_20_N_5_O_4_]^+^ [M + H]^+^: 394.1515; found: 394.1500.

*N*-((1-(4-methoxy benzyl)-1*H*-1,2,3-triazol-4-yl) methyl) (4-methoxy) cinnamide (**12**): 

White solid, obtained at 63% yield, m.p. 169.0–170.2 °C. IR (ATR) ${\bar{v}}_{max}$ (cm^−1^): 3266, 1605, 1509, 1246, 1030, 824. ^1^H NMR (400 MHz, DMSO-*d_6_*) δ: 3.73 (s, 3H), 3.78 (s, 3H), 4.40 (d, 2H, *J* = 5.6 Hz), 5.47 (s, 2H), 6.50 (d, 1H, *J* = 15.8 Hz), 6.92 (d, 2H, *J* = 8.6 Hz), 6.97 (d, 2H, *J* = 8.7 Hz), 7.30 (d, 2H, *J* = 8.6 Hz), 7.39 (d, 1H, *J* = 15.8 Hz), 7.49 (d, 2H, *J* = 8.7 Hz), 7.95 (s, 1H), 8.49 (t, 1H, *J* = 5.6 Hz). ^13^C NMR (100 MHz, DMSO-*d_6_*) δ: 34.3, 52.3, 55.1, 55.3, 114.1, 114.4, 119.4, 122.7, 127.4, 128.1, 129.1, 129.7, 138.7, 145.1, 159.2, 160.4, 165.2. HRMS (ESI^+^) calcd. for [C_21_H_23_N_4_O_3_]^+^ [M + H]^+^: 379.1770; found: 379.1741.

*N*-((1-(4-(trifluoromethoxy) benzyl)-1*H*-1,2,3-triazol-4-yl) methyl) (4-methoxy) cinnamide (**13**):

White solid, obtained at 85% yield, m.p. 191.4–193.0 °C. IR (ATR) ${\bar{v}}_{max}$ (cm^−1^): 3248, 1595, 1511, 1246, 831. ^1^H NMR (400 MHz, DMSO-*d_6_*) δ: 3.78 (s, 3H), 4.42 (d, 2H, *J* = 5.6 Hz), 5.62 (s, 2H), 6.50 (d, 1H, *J* = 15.8 Hz), 6.97 (d, 2H, *J* = 8.7 Hz), 7.36–7.42 (m, 3H), 7.45 (d, 2H, *J* = 8.5 Hz), 7.50 (d, 2H, *J* = 8.7 Hz), 8.06 (s, 1H), 8.50 (t, 1H, *J* = 5.6 Hz). ^13^C NMR (100 MHz, DMSO-*d_6_*) δ: 34.3, 51.8, 55.4, 55.6, 110.1, 111.8, 119.6, 121.3, 123.1, 127.6, 130.0, 135.6, 139.1, 145.2, 148.1, 148.9, 150.2, 165.2. HRMS (ESI^+^) calcd. for [C_21_H_20_F_3_N_4_O_3_]^+^ [M + H]^+^: 433.1488; found: 433.1465.

*N*-((1-(4-(trifluoromethyl) benzyl)-1*H*-1,2,3-triazol-4-yl) methyl) (4-methoxy) cinnamide (**14**): 

White solid, obtained at 78% yield, m.p. 220.5–221.9 °C. IR (ATR) ${\bar{v}}_{max}$ (cm^−1^): 3244, 1597, 1513, 1122, 828. ^1^H NMR (400 MHz, DMSO-*d_6_*) δ: 3.78 (s, 3H), 4.42 (d, 2H, *J* = 5.6 Hz), 5.70 (s, 2H), 6.50 (d, 1H, *J* = 15.8 Hz), 6.97 (d, 2H, *J* = 8.7 Hz), 7.40 (d, 1H, *J* = 15.8 Hz), 7.50 (d, 2H, *J* = 8.0 Hz), 7.51 (d, 2H, *J* = 8.7 Hz), 7.75 (d, 2H, *J* = 8.0 Hz), 8.07 (s, 1H), 8.51 (t, 1H, *J* = 5.6 Hz). ^13^C NMR (100 MHz, DMSO-*d_6_*) δ: 34.3, 52.1, 55.3, 114.4, 119.4, 123.4, 124.1 (d, *J*_C-F_ = 272 Hz), 125.7 (q, *J*_C-F_ = 4 Hz), 127.4, 128.6 (d, *J*_C-F_ = 32 Hz), 128.7, 129.1, 138.8, 140.8, 145.3, 160.4, 165.2. HRMS (ESI^+^) calcd. for [C_21_H_20_F_3_N_4_O_2_]^+^ [M + H]^+^: 417.1538; found: 417.1522.

*N*-((1-(3,4-difluoro benzyl)-1*H*-1,2,3-triazol-4-yl) methyl) (4-methoxy) cinnamide (**15**): 

White solid, obtained at 90% yield, m.p. 166.3–167.7 °C. IR (ATR) ${\bar{v}}_{max}$ (cm^−1^): 3346, 1601, 1513, 1172, 1028. ^1^H NMR (400 MHz, DMSO-*d_6_*) δ: 3.78 (s, 3H), 4.42 (d, 2H, *J* = 5.6 Hz), 5.57 (s, 2H), 6.50 (d, 1H, *J* = 15.9 Hz), 6.97 (d, 2H, *J* = 8.7 Hz), 7.17–7.20 (m, 1H), 7.40 (d, 1H, *J* = 15.9 Hz), 7.42–7.47 (m, 2H), 7.50 (d, 2H, *J* = 8.7 Hz), 8.05 (s, 1H), 8.50 (t, 1H, *J* = 5.6 Hz). ^13^C NMR (100 MHz, DMSO-*d_6_*) δ: 34.3, 51.5, 55.3, 114.4, 117.4 (d, *J*_C-F_ = 17.3 Hz), 117.9 (d, *J*_C-F_ = 17.3 Hz), 119.4, 123.1, 125.2–125.3 (dd, *J*_C-F_ = 3.5 Hz, *J*_C-F_ = 6.8 Hz), 127.4, 129.1, 133.7–133.8 (dd, *J*_C-F_ = 3.8 Hz, *J*_C-F_ = 5.8 Hz), 138.7, 145.3, 148.0–148.1 (dd, *J*_C-F_ = 3.7 Hz, *J*_C-F_ = 246 Hz), 150.4–150.5 (dd, *J*_C-F_ = 3.7 Hz, *J*_C-F_ = 246 Hz), 160.4, 165.2. HRMS (ESI^+^) calcd. for [C_20_H_19_F_2_N_4_O_2_]^+^ [M + H]^+^: 385.1476; found: 385.1458.

*N*-((1-(4-methyl benzyl)-1*H*-1,2,3-triazol-4-yl) methyl) (3,4-dimethoxy) cinnamide (**16**): 

White solid, obtained at 85% yield, m.p. 191.6–192.2 °C. IR (ATR) ${\bar{v}}_{max}$ (cm^−1^): 3218, 1594, 1514, 1258, 1019, 799. ^1^H NMR (400 MHz, DMSO-*d_6_*) δ: 2.27 (s, 3H), 3.78 (s, 6H), 3.78 (s, 6H, 2 x OCH_3_), 4.41 (d, 2H, *J* = 5.6 Hz), 5.51 (s, 2H), 6.53 (d, 1H, *J* = 15.7 Hz), 6.97 (d, 1H, *J* = 8.3 Hz), 7.09–7.11 (dd, 1H, *J* = 8.3 Hz, *J* = 1.8 Hz), 7.14 (d, 1H, *J* = 1.8 Hz), 7.17 (d, 2H, *J* = 8.1 Hz), 7.22 (d, 2H, *J* = 8.1 Hz), 7.38 (d, 1H, *J* = 15.7 Hz), 7.95 (s, 1H), 8.44 (t, 1H, *J* = 5.6 Hz). ^13^C NMR (100 MHz, DMSO-*d_6_*) δ: 20.7, 34.3, 52.5, 55.4, 55.5, 110.1, 111.8, 119.6, 121.3, 122.8, 127.6, 128.1, 129.3, 133.1, 137.5, 139.0, 145.1, 148.9, 150.1, 165.1. HRMS (ESI^+^) calcd. for [C_22_H_25_N_4_O_3_]^+^ [M + H]^+^: 393.1927; found: 393.1911.

*N*-((1-(4-isopropyl benzyl)-1*H*-1,2,3-triazol-4-yl) methyl) (3,4-dimethoxy) cinnamide (**17**): 

White solid, obtained at 61% yield, m.p. 175.5–176.4 °C. IR (ATR) ${\bar{v}}_{max}$ (cm^−1^): 3286, 2957, 1618, 1510, 1258, 808. ^1^H NMR (400 MHz, DMSO-*d_6_*) δ: 2.17 (d, 6H, *J* = 6.9 Hz), 2.86 (sept, 1H, *J* = 6.9 Hz), 3.78 (s, 6H, 2 x OCH_3_), 4.42 (d, 2H, *J* = 5.6 Hz), 5.52 (s, 2H), 6.54 (d. 1H, *J* = 15.7 Hz), 6.98 (d, 1H, *J* = 8.3 Hz), 7.10–7.12 (dd, 1H, *J* = 8.3 Hz, *J* = 1.7 Hz), 7.15 (d, 1H, *J* = 1.7 Hz), 7.23 (d, 2H, *J* = 8.5 Hz), 7.26 (d, 2H, *J* = 8.5 Hz), 7.39 (d, 1H, *J* = 15.7 Hz), 8.00 (s, 1H), 8.47 (t, 1H, *J* = 5.6 Hz). ^13^C NMR (100 MHz, DMSO-*d_6_*) δ: 24.2, 33.6, 34.8, 53.0, 55.8, 56.0, 110.5, 112.1, 120.0, 121.8, 123.3, 127.1, 128.1, 128.6, 134.0, 139.5, 145.6, 148.9, 149.3, 150.6, 165.6. HRMS (ESI^+^) calcd. for [C_24_H_29_N_4_O_3_]^+^ [M + H]^+^: 421.2240; found: 421.2215.

*N*-((1-(4-fluoro benzyl)-1*H*-1,2,3-triazol-4-yl) methyl) (3,4-dimethoxy) cinnamide (**18**): 

White solid, obtained at 65% yield, m.p. 174.8–175.5 °C. IR (ATR) ${\bar{v}}_{max}$ (cm^−1^): 3226, 1594, 1511, 1258, 1020, 801. ^1^H NMR (400 MHz, DMSO-*d_6_*) δ: 3.78 (s, 6H, 2 x OCH_3_), 4.42 (d, 2H, *J* = 5.6 Hz), 5.56 (s, 2H), 6.54 (d, 1H, *J* = 15.7 Hz), 6.97 (d, 1H, *J* = 8.3 Hz), 7.10–7.12 (dd, 1H, *J* = 8.3 Hz, *J* = 1.7 Hz), 7.14 (d, 1H, *J* = 1.7 Hz), 7.20 (t, 2H, *J* = 8.8 Hz), 7.37–7.41 (m, 3H), 8.01 (s, 1H), 8.46 (t, 1H, *J* = 5.6 Hz). ^13^C NMR (100 MHz, DMSO-*d_6_*) δ: 34.3, 51.9, 55.4, 55.5, 110.1, 111.8, 115.6 (d, *J*_C-F_ = 22 Hz), 119.6, 121.3, 122.9, 127.6, 130.3 (d, *J*_C-F_ = 8.4 Hz), 132.4 (d, *J*_C-F_ = 3 Hz) 139.1, 145.2, 148.9, 150.1, 161.9 (d, *J*_C-F_ = 244 Hz), 165.2. HRMS (ESI^+^) calcd. for [C_21_H_22_FN_4_O_3_]^+^ [M + H]^+^: 397.1676; found: 397.1649.

*N*-((1-(4-chloro benzyl)-1*H*-1,2,3-triazol-4-yl) methyl) (3,4-dimethoxy) cinnamide (**19**): 

White solid, obtained at 64% yield, m.p. 189.6–190.8 °C. IR (ATR) ${\bar{v}}_{max}$ (cm^−1^): 3225, 1594, 1515, 1258, 1018, 797. ^1^H NMR (400 MHz, DMSO-*d_6_*) δ: 3.78 (s, 6H, 2 x OCH_3_), 4.42 (d, 2H, *J* = 5.5 Hz), 5.57 (s, 2H), 6.53 (d, 1H, *J* = 15.8 Hz), 6.97 (d, 1H, *J* = 8.3 Hz), 7.11 (d, 1H, *J* = 8.3 Hz), 7.14 (s, 1H), 7.34 (d, 2H, *J* = 8.4 Hz), 7.39 (d, 1H, *J* = 15.8 Hz), 7.44 (d, 2H, *J* = 8.4 Hz), 8.02 (s, 1H), 8.46 (t, 1H, *J* = 5.5 Hz). ^13^C NMR (100 MHz, DMSO-*d_6_*) δ: 34.3, 51.9, 55.4, 55.5, 110.1, 111.8, 119.6, 121.3, 123.0, 127.6, 128.7, 129.9, 132.8, 135.1, 139.1, 145.2, 148.9, 150.1, 165.2. HRMS (ESI^+^) calcd. for [C_21_H_22_ClN_4_O_3_]^+^ [M + H]^+^: 413.1380; found: 413.1363.

*N*-((1-(4-bromo benzyl)-1*H*-1,2,3-triazol-4-yl) methyl) (3,4-dimethoxy) cinnamide (**20**): 

White solid, obtained at 73% yield, m.p. 198.2–199.6 °C. IR (ATR) ${\bar{v}}_{max}$ (cm^−1^): 3281, 1594, 1514, 1257, 1018, 801. ^1^H NMR (400 MHz, DMSO-*d_6_*) δ: 3.78 (s, 6H, 2 x OCH_3_), 3.78 (s, 3H), 4.42 (d, 2H, *J* = 5.6 Hz), 5.56 (s, 2H), 6.53 (d, 1H, *J* = 15.7 Hz), 6.98 (d, 2H, *J* = 8.3 Hz), 7.10–7.12 (dd, 1H, *J* = 8.3 Hz, *J* = 1.7 Hz), 7.14 (d, 1H, *J* = 1.7 Hz), 7.28 (d, 2H, *J* = 8.4 Hz), 7.39 (d, 1H, *J* = 15.7 Hz), 7.57 (d, 2H, *J* = 8.4 Hz), 8.02 (s, 1H), 8.45 (t, 1H, *J* = 5.6 Hz). ^13^C NMR (100 MHz, DMSO-*d_6_*) δ: 34.3, 52.0, 55.4, 55.5, 110.1, 111.8, 119.6, 121.3, 121.4, 123.0, 127.6, 130.2, 131.7, 135.5, 139.0, 145.2, 148.9, 150.1, 165.2. HRMS (ESI^+^) calcd. for [C_21_H_22_BrN_4_O_3_]^+^ [M + H]^+^: 457.0875; found: 457.0843.

*N*-((1-(4-iodo benzyl)-1*H*-1,2,3-triazol-4-yl) methyl) (3,4-dimethoxy) cinnamide (**21**): 

White solid, obtained at 87% yield, m.p. 194.0–194.9 °C. IR (ATR) ${\bar{v}}_{max}$ (cm^−1^): 3282, 1651, 1510, 1259, 1007, 810. ^1^H NMR (400 MHz, DMSO-*d_6_*) δ: 3.78 (s, 6H, 2 x OCH_3_), 3.78 (s, 3H), 4.41 (d, 2H, *J* = 5.6 Hz), 5.53 (s, 2H), 6.53 (d, 1H, *J* = 15.7 Hz), 6.97 (d, 2H, *J* = 8.3 Hz), 7.09–7.14 (m, 4H), 7.38 (d, 1H, *J* = 15.7 Hz), 7.74 (d, 2H, *J* = 8.2 Hz), 8.00 (s, 1H), 8.45 (t, 1H, *J* = 5.6 Hz). ^13^C NMR (100 MHz, DMSO-*d_6_*) δ: 34.3, 52.1, 55.4, 55.6, 94.4, 110.1, 111.8, 119.6, 121.3, 123.4, 123.1, 130.3, 135.9, 137.5, 139.1, 145.2, 148.9, 150.2, 165.2. HRMS (ESI^+^) calcd. for [C_21_H_22_IN_4_O_3_]^+^ [M + H]^+^: 505.0737; found: 505.0715.

*N*-((1-(4-nitro benzyl)-1*H*-1,2,3-triazol-4-yl) methyl) (3,4-dimethoxy) cinnamide (**22**):

White solid, obtained at 63% yield, m.p. 217.6–218.9 °C. IR (ATR) ${\bar{v}}_{max}$ (cm^−1^): 3242, 1595, 1515, 1344, 1258, 1017, 804. ^1^H NMR (400 MHz, DMSO-*d_6_*) δ: 3.78 (s, 6H, 2 x OCH_3_), 4.44 (d, 2H, *J* = 5.6 Hz), 5.76 (s, 2H), 6.54 (d, 1H, *J* = 15.7 Hz), 6.97 (d, 1H, *J* = 8.3 Hz), 7.10–7.12 (dd, 1H, *J* = 8.3 Hz, *J* = 1.7 Hz), 7.14 (d, 1H, *J* = 1.7 Hz), 7.39 (d, 1H, *J* = 15.7 Hz), 7.54 (d, 2H, *J* = 8.7 Hz), 8.09 (s, 1H), 8.23 (d, 2H, *J* = 8.7 Hz), 8.48 (t, 1H, *J* = 5.6 Hz). ^13^C NMR (100 MHz, DMSO-*d_6_*) δ: 34.3, 51.8, 55.4, 55.5, 110.1, 111.8, 119.6, 121.3, 123.0, 123.9, 127.6, 129.1, 139.1, 143.5, 145.4, 147.2, 148.9, 150.2, 165.2. HRMS (ESI^+^) calcd. for [C_21_H_22_N_5_O_5_]^+^ [M + H]^+^: 424.1621; found: 424.1602.

*N*-((1-(4-methoxy benzyl)-1*H*-1,2,3-triazol-4-yl) methyl) (3,4-dimethoxy) cinnamide (**23**): 

White solid, obtained at 85% yield, m.p. 171.2–172.0 °C. IR (ATR) ${\bar{v}}_{max}$ (cm^−1^): 3258, 1600, 1510, 1025. ^1^H NMR (400 MHz, DMSO-*d_6_*) δ: 3.73 (s, 3H, OCH_3_), 3.78 (s, 6H, 2 x OCH_3_), 4.40 (d, 2H, *J* = 5.6 Hz), 5.48 (s, 2H), 6.53 (d, 1H, *J* = 15.7 Hz), 6.92 (d, 2H, *J* = 8.6 Hz), 6.97 (d, 2H, *J* = 8.3 Hz), 7.09–7.11 (dd, 1H, *J* = 8.3 Hz, *J* = 1.7 Hz), 7,14 (d, 1H, *J* = 1.7 Hz), 7.30 (d, 2H, *J* = 8.6 Hz), 7.38 (d, 1H, *J* = 15.7 Hz), 7.94 (s, 1H), 8.44 (t, 1H, *J* = 5.6 Hz). ^13^C NMR (100 MHz, DMSO-*d_6_*) δ: 34.3, 52.3, 55.1, 55.4, 55.5, 110.1, 111.8, 114.1, 119.6, 121.3, 122.6, 127.6, 128.0, 129.7, 139.1, 145.1, 148.9, 150.1, 159.1, 165.2. HRMS (ESI^+^) calcd. for [C_22_H_25_N_4_O_4_]^+^ [M + H]^+^: 409.1876; found: 409.1855.

*N*-((1-(4-(trifluoromethoxy) benzyl)-1*H*-1,2,3-triazol-4-yl) methyl) (3,4-dimethoxy) cinnamide (**24**):

White solid, obtained at 91% yield, m.p. 177.4–178.6 °C. IR (ATR) ${\bar{v}}_{max}$ (cm^−1^): 3258, 1593, 1514, 1256, 1021, 772. ^1^H NMR (400 MHz, DMSO-*d_6_*) δ: 3.78 (s, 6H, 2 x OCH_3_), 4.40 (d, 2H, *J* = 5.6 Hz), 5.48 (s, 2H), 6.53 (d, 1H, *J* = 15.7 Hz), 6.97 (d, 2H, *J* = 8.3 Hz), 7.09–7.11 (dd, 1H, *J* = 8.3 Hz, *J* = 1.7 Hz), 7.14 (d, 1H, *J* = 1.7 Hz), 7.38 (d, 1H, *J* = 15.7 Hz), 8.05 (s, 1H), 8.46 (t, 1H, *J* = 5.6 Hz). ^13^C NMR (100 MHz, DMSO-*d_6_*) δ: 34.3, 51.8, 55.4, 55.5, 110.1, 111.8, 119.6, 121.3, 123.1, 127.6, 130.0, 135.6, 139.1, 145.2, 148.1, 148.9, 150.2, 165.2. HRMS (ESI^+^) calcd. for [C_22_H_22_F_3_N_4_O_4_]^+^ [M + H]^+^: 463.1593; found: 463.1570.

*N*-((1-(4-(trifluoromethyl) benzyl)-1*H*-1,2,3-triazol-4-yl) methyl) (3,4-dimethoxy) cinnamide (**25**): 

White solid, obtained at 76% yield, m.p. 196.4–197.6 °C. IR (ATR) ${\bar{v}}_{max}$ (cm^−1^): 3243, 1592, 1515, 1120, 853. ^1^H NMR (400 MHz, DMSO-*d_6_*) δ: 3.78 (s, 6H, 2 x OCH_3_), 4.43 (d, 2H, *J* = 5.6 Hz), 5.70 (s, 2H), 6.53 (d, 1H, *J* = 15.7 Hz), 6.97 (d, 2H, *J* = 8.3 Hz), 7.10–7.12 (dd, 1H, *J* = 8.3 Hz, *J* = 1.7 Hz), 7.14 (d, 1H, *J* = 1.7 Hz), 7.39 (d, 1H, *J* = 15.7 Hz), 7.51 (d, 2H, *J* = 8.1 Hz), 7.75 (d, 2H, *J* = 8.1 Hz) 8.07 (s, 1H), 8.47 (t, 1H, *J* = 5.6 Hz). ^13^C NMR (100 MHz, DMSO-*d_6_*) δ: 34.3, 52.1, 55.4, 55.6, 110.1, 111.8, 119.6, 121.4, 123.3, 124.1 (d, *J*_C-F_ = 272 Hz), 125.7 (q, *J*_C-F_= 3.7 Hz), 127.6, 128.7, 128.7 (d, *J*_C-F_ = 32 Hz), 139.1, 140.8, 145.3, 148.9, 150.2, 165.2. HRMS (ESI^+^) calcd. for [C_22_H_22_F_3_N_4_O_2_Na]^+^ [M + Na]^+^: 469.1463; found: 469.1447.

*N*-((1-(3,4-difluoro benzyl)-1*H*-1,2,3-triazol-4-yl) methyl) (3,4-dimethoxy) cinnamide (**26**): 

White solid, obtained at 81% yield, m.p. 166.0–167.2 °C. IR (ATR) ${\bar{v}}_{max}$ (cm^−1^): 3354, 1598, 1515, 1267, 1019, 808. ^1^H NMR (400 MHz, DMSO-*d_6_*) δ: 3.78 (s, 3H), 3.78 (s, 3H), 4.42 (d, 2H, *J* = 5.6 Hz), 5.57 (s, 2H), 6.53 (d, 1H, *J* = 15.7 Hz), 6.97 (d, 1H, *J* = 8.3 Hz), 7.10–7.12 (dd, 1H, *J* = 8.3 Hz, *J* = 1.7 Hz), 7.14 (d, 1H, *J* = 1.7 Hz), 7.17–7.20 (m, 1H), 7.39 (d, 1H, *J* = 15.7 Hz), 7.42–7.48 (m, 2H), 8.05 (s, 1H), 8.46 (t, 1H, *J* = 5.6 Hz). ^13^C NMR (100 MHz, DMSO-*d_6_*) δ: 34.3, 51.5, 55.4, 55.5, 110.1, 111.8, 117.4 (d, *J* = 17.6 Hz), 117.9 (d, *J* = 17.6 Hz), 119.6, 121.3, 123.1, 125.2–125.3 (dd, *J* = 6.8 Hz, *J* = 3.5 Hz), 127.6, 133.7–133.8 (dd, *J* = 5.8 Hz, *J* = 3.7 Hz), 139.1, 145.3, 148.0–148.1 (dd, *J* = 3.6 Hz, *J* = 246 Hz), 148.1–150.5 (dd, *J* = 246 Hz, *J* = 3.6 Hz), 148.9, 150.2, 165.2. HRMS (ESI^+^) calcd. for [C_21_H_21_F_2_N_4_O_3_]^+^ [M + H]^+^: 415.1582; found: 415.1559.

*N*-((1-(7-(diethylamino)-2-oxo-2*H*-chromen-3-yl)-1*H*-1,2,3-triazol-4-yl) methyl) (4-methoxy) cinnamide (**27**): 

White solid, obtained at 52% yield, m.p. 191.3–192.7 °C. IR (ATR) ${\bar{v}}_{max}$ (cm^−1^): 3290, 2969, 1715, 1597, 821. ^1^H NMR (400 MHz, DMSO-*d_6_*) δ: 1.13 (t, 6H, *J* = 7.0 Hz), 3.46 (q, 4H, *J* = 7.0 Hz), 3.78 (s, 3H), 4.52 (d, 2H, *J* = 5.5 Hz), 6.54 (d, 1H, *J* = 15.9 Hz), 6.64 (s, 1H), 6.81 (d, 1H, *J* = 8.3 Hz), 6.97 (d, 2H, *J* = 8.6 Hz), 7.43 (d, 1H, *J* = 15.9 Hz), 7.51 (d, 2H, *J* = 8.6 Hz), 7.62 (d, 1H, *J* = 8.3 Hz), 8.38 (s, 1H), 8.43 (s, 1H), 861 (t, 1H, *J* = 5.5 Hz). ^13^C NMR (100 MHz, DMSO-*d_6_*) δ: 12.3, 34.2, 44.2, 55.3, 96.4, 106.5, 110.0, 114.4, 116.3, 119.3, 123.7, 127.4, 129.2, 130.5, 136.6, 138.9, 145.0, 151.4, 155.6, 156.7, 160.4, 165.3. HRMS (ESI^+^) calcd. for [C_26_H_28_N_5_O_4_]^+^ [M + H]^+^: 474.2175; found: 474.2124.

*N*-((1-(7-(diethylamino)-2-oxo-2*H*-chromen-3-yl)-1*H*-1,2,3-triazol-4-yl)methyl) (3,4-dimethoxy) cinnamide (**28**): 

White solid, obtained at 57% yield, m.p. 197.0–198.4 °C IR (ATR) ${\bar{v}}_{max}$ (cm^−1^): 3292, 2973, 1729, 1613, 815. ^1^H NMR (400 MHz, DMSO-*d_6_*) δ: 1.13 (t, 6H, *J* = 7.0 Hz), 3.48 (q, 4H, *J* = 7.0 Hz), 3.80 (s, 3H), 3.81 (s, 3H), 4.54 (d, 2H, *J* = 5.5 Hz), 6.59 (d, 1H, *J* = 15.8 Hz), 6.67 (d, 1H, *J* = 2.0 Hz), 6.82–6.84 (dd, 1H, *J* = 9.0 Hz, *J* = 2.0 Hz), 7.00 (d, 2H, *J* = 8.3 Hz), 7.13–7.15 (dd, 1H, *J* = 8.3 Hz, *J* = 1.6 Hz), 7.18 (d, 1H, *J* = 1.6 Hz), 7.43 (d, 1H, *J* = 15.8 Hz), 7.64 (d, 1H, *J* = 9.0 Hz), 8.41 (s, 1H), 8.46 (s, 1H), 8.59 (t, 1H, *J* = 5.5 Hz). ^13^C NMR (100 MHz, DMSO-*d_6_*) δ: 12.3, 34.2, 44.2, 55.4, 55.5, 96.4, 106.5, 110.0, 110.1, 111.8, 116.3, 119.6, 121.3, 123.6, 127.6, 130.5, 136.5, 139.2, 145.0, 149.0, 150.1, 151.4, 155.6, 156.7, 165.3. HRMS (ESI^+^) calcd. for [C_27_H_30_N_5_O_5_]^+^ [M + H]^+^: 504.2247; found: 504.2222.

### 4.3. Parasite Culture, Antileishmanial Action, and Cytotoxicity in Mammalian Cells

The study was approved by the Committee for the Ethical Handling of Research Animals from the Federal University of Minas Gerais (protocol number 085/2017). *Leishmania braziliensis* (MHOM/BR/1975/M2904) promastigotes were grown at 24 °C in Schneider’s medium (Sigma-Aldrich, USA) supplemented with 20% heat-inactivated fetal bovine serum (FBS, Sigma-Aldrich, USA) and 20 mmol/L L-glutamine at pH 7.4 [65].

To obtain the axenic amastigotes, 1 × 10^9^ stationary promastigotes were washed three times in sterile phosphate-buffered saline (PBS) and incubated in 5 mL FBS for 48 h at 37 °C. Parasites were washed with cold PBS, and their morphology was evaluated with an optical microscope after Giemsa staining as previously described [66]. 

The in vitro antileishmanial activity was evaluated by incubating the promastigotes or axenic amastigotes (1 × 10^6^ cells/well) with the 24 synthetic derivatives (Compounds **5**–**28**; 0 to 600 µg/mL) in 96-well culture plates (Nunc, Nunclon, Roskilde, Denmark) for 48 h at 24 °C. Due to the difficulty in obtaining a high concentration of murine macrophages, the cytotoxicity experiment was evaluated by incubating such cells (5 × 10^5^ per mL) with Compound **28** (0 to 600 µg/mL), which presented the best antileishmanial activity, for 48 h at 37 °C in a 5% CO_2_ environment. AmpB (0 to 10 µg/mL; Sigma-Aldrich, St. Louis, MO, USA) was used as a positive control in both experiments. The cell viability was assessed using the 3-(4.5-dimethylthiazol-2-yl)-2.5-diphenyl tetrazolium bromide (MTT, Sigma-Aldrich, St. Louis, MO, USA) method. The optical density (OD) values were read using a microplate spectrophotometer (Molecular Devices, Spectra Max Plus, San Jose, CA, USA) at 570 nm. Results were entered into Microsoft Excel (version 10.0) spreadsheets, and the 50% *Leishmania* and macrophage inhibitory concentrations (IC_50_ and CC_50_, respectively) were calculated by applying a sigmoidal regression of the dose–response curve [67]. The SI was calculated by dividing the CC_50_ with the respective IC_50_ values.

### 4.4. Treatment of Infected Macrophages and Inhibition of Infection

Murine macrophages (5 × 10^5^ cells) were plated on round glass coverslips in 24-well plates in RPMI 1640 medium with added 20% FBS and 20 mmol/L L-glutamine at pH 7.4 and incubated for 24 h at 37 °C in 5% CO_2_. Promastigotes were added to the wells (at a ratio of 10 parasites per macrophage), and cultures were incubated for 48 h at 37 °C in a 5% CO_2_ environment. Free parasites were removed by extensive washing with RPMI 1640 medium, and the infected macrophages were treated with Compound **28** or AmpB (0, 1.0, 2.5 and 5.0 µg/mL in both cases) for 48 h at 37 °C in 5% CO_2_. 

To evaluate the inhibition of infection, promastigotes (5 × 10^6^ cells) were first incubated with Compound **28** or AmpB (0, 1.0, 2.5 and 5.0 µg/mL in both cases) for 4 h at 24 °C; these were then used to infect murine macrophages (at a ratio of 10 parasites per macrophage) and incubated for 24 h at 37 °C in 5% CO_2_. Then, cells were fixed with 4% paraformaldehyde, washed, and stained with Giemsa. The percentage of infected macrophages and the number of recovered amastigotes were determined by counting 200 cells, in triplicate, using an optical microscope.

### 4.5. ROS Levels in Leishmania-Infected Macrophages

Macrophages (1 × 10^6^ cells/mL) were incubated in 96-well culture plates (Nunc, Nunclon, Roskilde, Denmark) and infected with *L. braziliensis* promastigotes (at a ratio of 10 parasites per cell). The infected macrophages were either left untreated or treated with Compound **28** (at a concentration of 5.0 µg/mL) for 48 h at 37 °C in 5% CO_2_. The cells were washed with PBS and incubated with 20 μmol/L H_2_DCFDA (Molecular Probes, Eugene, Oregon, USA) for 30 min in the dark. The fluorescence intensity was measured using a spectrofluorometer (FLx800; BioTek Instruments, Winooski, VT, USA) at 485 nm excitation and 528 nm emission. As a positive control, the infected macrophages were incubated with H_2_O_2_ at a concentration of 0.02 µg/mL (0.5 µmol/L). Parasites that were untreated with Compound **28** and not labeled with the fluorescent probe were used as the sample blank.

### 4.6. Depolarization of Mitochondrial Membrane Potential (ΔΨm)

*Leishmania braziliensis* promastigotes (1 × 10^7^ cells/mL) were treated with compound **28** (at a concentration corresponding to one IC_50_ value) for 48 h at 24 °C. The parasites (3 × 10^6^ cells/mL) were then labeled with JC-1 (5 µg/mL, Sigma-Aldrich, St. Louis, MO, USA) for 30 min at 37 °C. After washing twice with PBS to remove the non-internalized dye, the samples were added to a black 96-well plate, and the ΔΨm was analyzed in a spectrofluorometer (FLx800, BioTek Instruments, Winooski, VT, USA) with an excitation filter of 485 nm and emission wavelength of 528 nm and 540 nm to measure the green and red fluorescence, respectively. The ΔΨm was determined using the ratio between the red/green fluorescence intensity. Parasites that were exposed to 1.3 µg/mL (5.0 µmol/L) of FCCP (Sigma-Aldrich, St. Louis, MO, USA) for 20 min were used as a control [68]. Parasites that were untreated or treated with Compound **28** and not labeled with the fluorescent probe were used as the sample blank.

### 4.7. Evaluation of Reactive Oxygen Species (ROS) Production

After treatment with Compound **28** (at a concentration corresponding to one IC_50_ value) for 48 h at 24 °C, *L. braziliensis* promastigotes were washed with PBS, and the parasite concentration was adjusted to 3 × 10^6^ cells per well. H_2_DCFDA (Sigma-Aldrich, St. Louis, MO, USA) was then added and incubated for 30 min in the dark, and samples were read in a fluorometric microplate reader (FLx800, BioTek Instruments, Winooski, VT, USA) with excitation and emission wavelengths of 485 and 528 nm, respectively [68]. Parasites that were untreated or treated with Compound **28** and not labeled with the fluorescent probe were used as the blank.

### 4.8. Membrane Permeability Evaluation

*L. braziliensis* promastigotes (1 × 10^7^ cells/mL) were incubated with Compound **28** (at a concentration corresponding to one IC_50_ value) for 48 h at 24 °C. After washing with PBS, parasites (3 × 10^6^ cells/well) were incubated with 1.0 µg/mL PI (Sigma-Aldrich, St. Louis, MO, USA) in the dark for 15 min. The samples were analyzed using a fluorometer microplate reader (FLx800, BioTek Instruments, Winooski, VT, USA), with excitation and emission wavelengths of 485 and 528 nm, respectively [68]. Parasites that were killed by heating to 65 °C for 10 min were used as controls for the membrane permeabilization. Parasites that were untreated or treated with Compound **28** and not labeled with the fluorescent probe were used as the sample blank.

## 5. Conclusions

In conclusion, a series of twenty-four 1,2,3-triazole-containing cinnamides were prepared and tested against the *L. braziliensis* promastigote and axenic amastigote forms. Among the evaluated cinnamides, Compound **28** was the only one with relevant antileishmanial activity, demonstrating low toxicity in mammalian cells and showing better SI values compared with AmpB. The compound was effective at reducing the infection percentage and parasite load in treated macrophages, and it also exhibited inhibitory effects when parasites were pre-incubated with it, indicating its potential as a preventive/prophylactic agent. Moreover, the cinnamide **28** induced an increase in reactive oxygen species (ROS) production, causing mitochondrial depolarization and disruption of the membrane, leading to the death of *L. braziliensis*. These findings suggest that Compound **28** holds promise for further evaluation in mammalian models as an antileishmanial agent for the treatment of TL.

## Figures and Tables

**Table 1:** Yields obtained in the preparation of Compounds **5**–**28**.

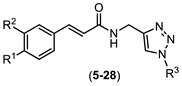				
Compound	R^1^	R^2^	R^3^	Yield (%)
**5**	OMe	H	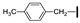	84
**6**	OMe	H	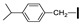	67
**7**	OMe	H	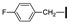	90
**8**	OMe	H	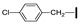	75
**9**	OMe	H	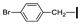	65
**10**	OMe	H	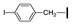	76
**11**	OMe	H	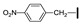	65
**12**	OMe	H	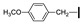	63
**13**	OMe	H	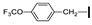	85
**14**	OMe	H	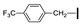	78
**15**	OMe	H	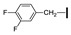	90
**16**	OMe	OMe	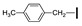	85
**17**	OMe	OMe	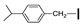	61
**18**	OMe	OMe	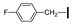	65
**19**	OMe	OMe	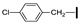	64
**20**	OMe	OMe	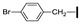	73
**21**	OMe	OMe	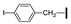	87
**22**	OMe	OMe	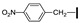	63
**23**	OMe	OMe	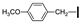	85
**24**	OMe	OMe	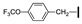	91
**25**	OMe	OMe	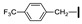	76
**26**	OMe	OMe	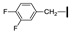	81
**27**	OMe	H	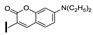	52
**28**	OMe	OMe	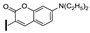	57

**Table 2:** **In vitro antileishmanial activity and cytotoxicity.** *Leishmania braziliensis* stationary promastigotes and axenic amastigotes (1 × 10^6^ cells each) were incubated with the 24 synthetic derivatives (Compounds **5**–**28**; 0 to 600 µg/mL) or AmpB (0 to 10 µg/mL) for 48 h at 24 °C. The cytotoxicity was evaluated by incubating murine cells (5 × 10^5^ per mL) with Compound **28** (0 to 600 µg/mL), which presented higher antileishmanial activity, for 48 h at 37 °C in 5% CO_2_. AmpB (0 to 10 µg/mL; Sigma-Aldrich, USA) was used as a control. Cell viability was assessed by the 3-(4.5-dimethylthiazol-2-yl)-2.5-diphenyl tetrazolium bromide (MTT, Sigma-Aldrich, USA) method. The optical density (OD) values were measured at 570 nm using a spectrophotometer, and results were recorded in Microsoft Excel (version 10.0) spreadsheets. The 50% *Leishmania* and macrophage inhibitory concentrations (IC_50_ and CC_50_, respectively) were calculated by applying a sigmoidal regression to the dose–response curve. The results are presented as the mean ± standard deviation.

Compound	IC_50_ (µg/mL)		CC_50_ (µg/mL)
	Promastigotes	Axenic Amastigotes	
**5**	>600.0	>600.0	-
**6**	>600.0	>600.0	-
**7**	>600.0	>600.0	-
**8**	>600.0	>600.0	-
**9**	>600.0	>600.0	-
**10**	>600.0	>600.0	-
**11**	>600.0	>600.0	-
**12**	>600.0	>600.0	-
**13**	>600.0	>600.0	-
**14**	>600.0	>600.0	-
**15**	>600.0	>600.0	-
**16**	>600.0	>600.0	-
**17**	>600.0	>600.0	-
**18**	>600.0	>600.0	-
**19**	>600.0	>600.0	-
**20**	>600.0	>600.0	-
**21**	>600.0	>600.0	-
**22**	>600.0	>600.0	-
**23**	>600.0	>600.0	-
**24**	>600.0	>600.0	-
**25**	>600.0	>600.0	-
**26**	>600.0	>600.0	-
**27**	>600.0	>600.0	-
**28**	105.7 ± 16.7	87.8 ± 9.7	1169.0 ± 113.2
Amphotericin B	0.974 ± 0.2	1.30 ± 0.4	8.90 ± 0.8

(-): Not determined.

**Table 3:** **Treatment of infected macrophages and infection inhibition.** Murine macrophages were infected with *L. braziliensis* promastigotes (at a ratio of 10 parasites per macrophage) and treated with Compound **28** or Amp B (0, 1.0, 2.5, and 5.0 µg/mL in both cases) for 48 h at 37 °C in a 5% CO_2_ environment. In another experiment, parasites were first incubated with the aforementioned compounds (at the same concentrations) for 1 h at 24 °C and then used to infect murine macrophages (at a ratio of 10 parasites per cell). In both analyses, the percentage of infection and the number of recovered amastigotes per infected cell were determined after counting 200 macrophages in triplicate. Results were expressed as the mean ± standard deviation.

Compounds	Concentration (µg/mL)	Percentage of Infected Macrophages after Treatment/Number of Amastigotes per Cell	Percentage of Infected Macrophages Using Pre-Incubated Parasites/Number of Amastigotes per Cell
**28**	5.0	60.2/2.9	71.5/3.6
	2.5	70.3/4.0	80.5/4.9
	1.0	79.5/6.7	84.5/5.8
	0	92.8/10.3	90.5/8.9
Amphotericin B	5.0	19.8/1.8	51.4/5.3
	2.5	27.6/3.5	67.6/6.5
	1.0	39.1/4.2	90.5/8.9
	0	92.8/10.3	90.5/8.9

## Data Availability

Data is contained within the article and Supplementary Material.

## Supplementary Materials

The following supporting information can be downloaded at: https://www.mdpi.com/article/10.3390/ph16081113/s1, Figures S1–S96.
