# Supplementary material for: Synthesis of 1,2,3-Triazole-Containing Methoxylated Cinnamides and Their Antileishmanial Activity against the Leishmania braziliensis Species
Source: Pharmaceuticals (Basel). 2023 Aug 7;16(8):1113. doi: 10.3390/ph16081113 (PMC10459042; doi:10.3390/ph16081113)
Supplement: Supplementary file 1 [file pharmaceuticals-16-01113-s001.zip › pharmaceuticals-2428487-supplementary.pdf]

SUPPLEMENTARY MATERIAL

Synthesis and antileishmanial activity against *Leishmania braziliensis* species of 1,2,3-triazole containing methoxylated cinnamides

Fabíola Suelen dos Santos[1], Rossimiriam Pereira de Freitas[1], Camila Simões de Freitas[2], Débora Vasconcelos Costa Mendonça[2], Daniela Pagliara Lage[2]; Grasiele Vieira de Souza Tavares[2], Amanda Sanchez Machado[2], Vivian Tamieti Martins[2], Adílson Vidal Costa[3], Vagner Tebaldi Queiroz[3], Mariana Belizário de Oliveira[3], Fabrício Marques de Oliveira[4], Luciana Maria Ribeiro Antinarelli[5], Elaine Soares Coimbra[5], Eduardo Jorge Pilau[6], Geovane Perez da Silva[6], Eduardo Antonio Ferraz Coelho[2], and Róbson Ricardo Teixeira[7,*]

[1] Laboratório de Síntese Orgânica (LABSINTO), Departamento de Química, Universidade Federal de Minas Gerais, Belo Horizonte, Minas Gerais, Brazil; fabiolasuelen92@hotmail.com (F.S. dos S.); rossipfreitas@gmail.com (R.P.F.)
[2] Programa de Pós-Graduação em Ciências da Saúde: Infectologia e Medicina Tropical, Faculdade de Medicina, Universidade Federal de Minas Gerais, Belo Horizonte, Minas Gerais, Brazil; camilasimoesf@gmail.com (C.S. de F.); eduardoferrazcoelho@yahoo.com.br (E. A. F. C.); debs.mendonca@gmail.com (D.V.C.M.); danipagliara@hotmail.com (D. P. L.); grasysv@hotmail.com (G.V. de S.T.); manda_sanchez92@hotmail.com (A. S. M.); viviantamietti@yahoo.com.br (V. T. M.)
[3] Grupo de Estudo Aplicado em Produtos Naturais e Síntese Orgânica (GEAPS), Departamento de Química e Física, Universidade Federal do Espírito Santo, Alegre, Espírito Santo, Brazil; avcosta@hotmail.com (A. V. C.); vagner.queiroz@ufes.br (V. T. Q); mariana.b.oliveira@edu.ufes.br (M. B. de O.)
[4] Instituto Federal de Minas Gerais (IFMG), Campus Ouro Branco, Ouro Branco, Ouro Branco, Minas Gerais, Brazil; fabrício.marques@ifmg.edu.br
[5] Departamento de Parasitologia, Microbiologia e Imunologia, Instituto de Ciências Biológicas, Universidade Federal de Juiz de Fora, Juiz de Fora, Minas Gerais, Brazil; lucianantinarelli@yahoo.com.br (L. M. R. A.); elaine.coimbra@ufjf.edu.br (E. S.C.);
[6] Departamento de Química, Centro de Ciências Exatas, Universidade Estadual de Maringá, Maringá, Paraná, Brazil; ejpilau@uem.br (E. J. P.); geovane.perez13@gmail.com (G. P. da S.)
[7] Grupo de Síntese e Pesquisa de Compostos Bioativos (GSPCB), Departamento de Química, Universidade Federal de Viçosa, 36570-900, Viçosa, Minas Gerais, Brazil.
* Correspondence: robsonr.teixeira@ufv.br

For the presentation of the spectra, the following abbreviation were utilized:

IR………………………………………....Infrared

ATR………………………………………Attenuated total reflectance

^{1}H NMR…………………………………Hydrogen-1 nuclear magnetic resonance

^{13}C NMR…………………………………Carbon-13 nuclear magnetic resonance

HRMS……………………………………..High resolution mass spectrometry

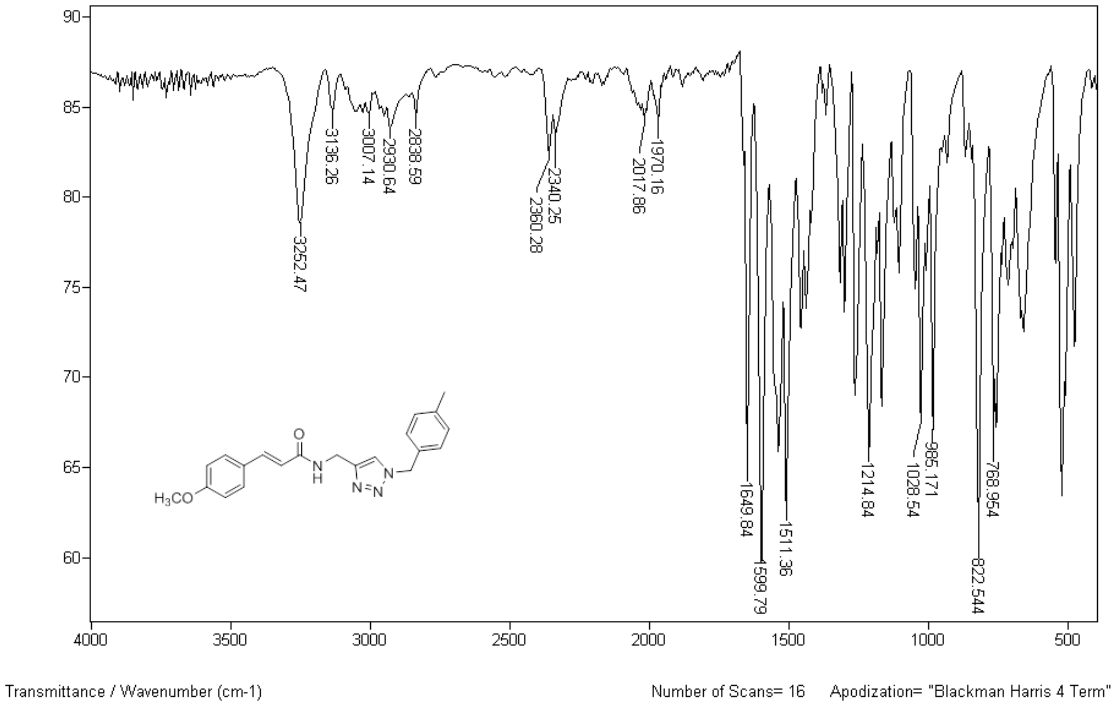

Figure S1 - IR spectrum (ATR) of **5**.

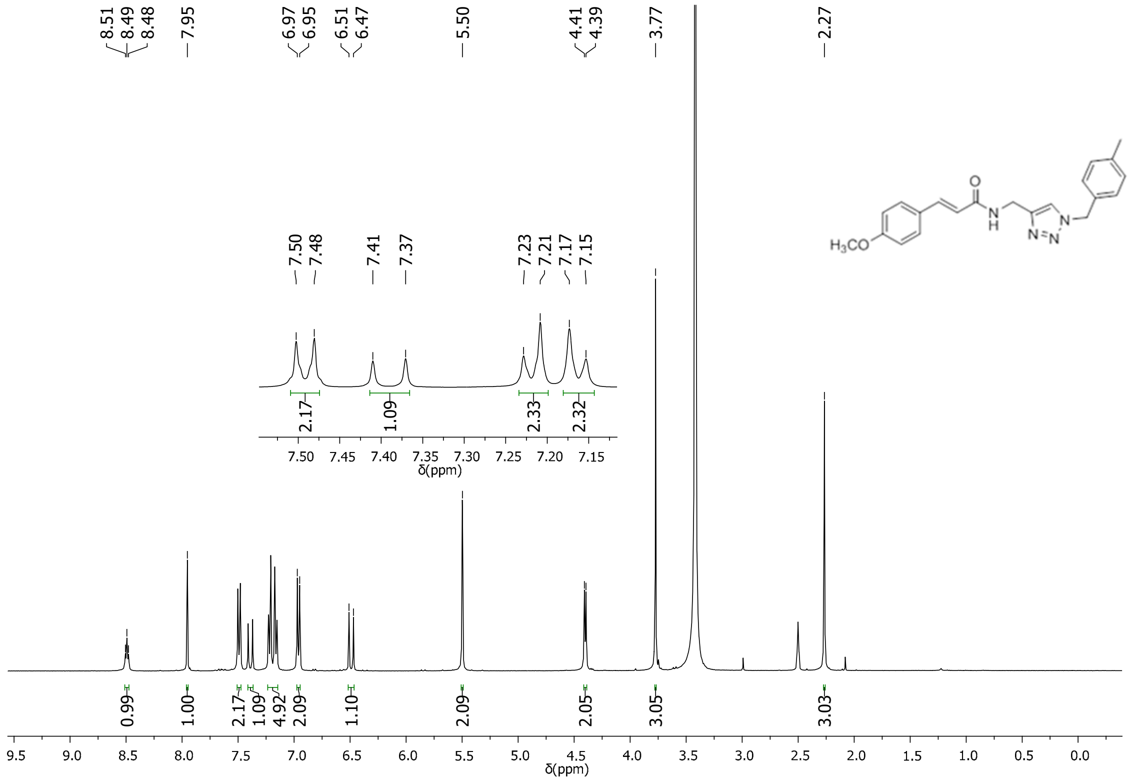

Figure S2 - ^{1}H NMR spectrum (400 MHz, DMSO-d_6) of **5**.

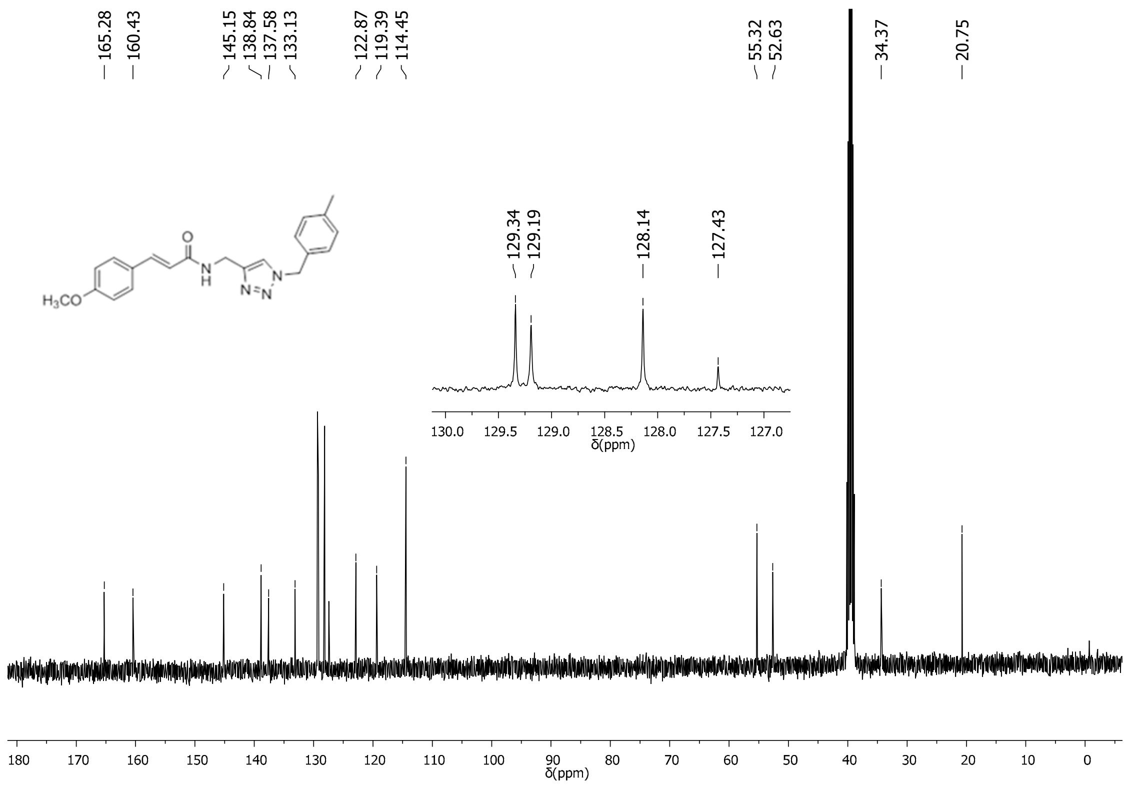

Figure S3 - ^{13}C NMR spectrum (100 MHz, DMSO-d_6) of **5**.

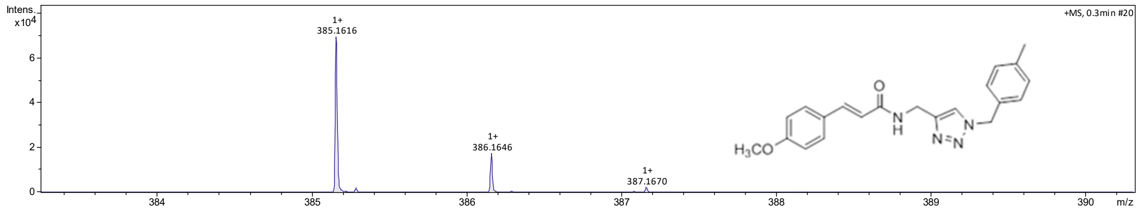

Figure S4 - HRMS spectrum of **5**.

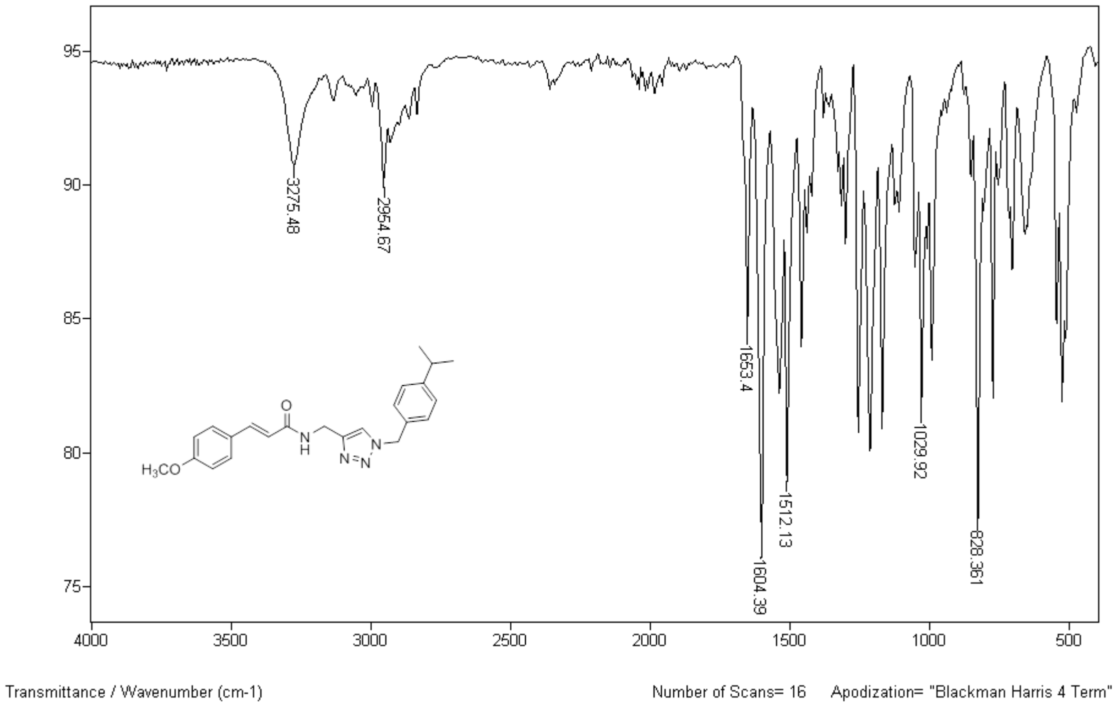

Figure S5 - IR spectrum (ATR) of **6**.

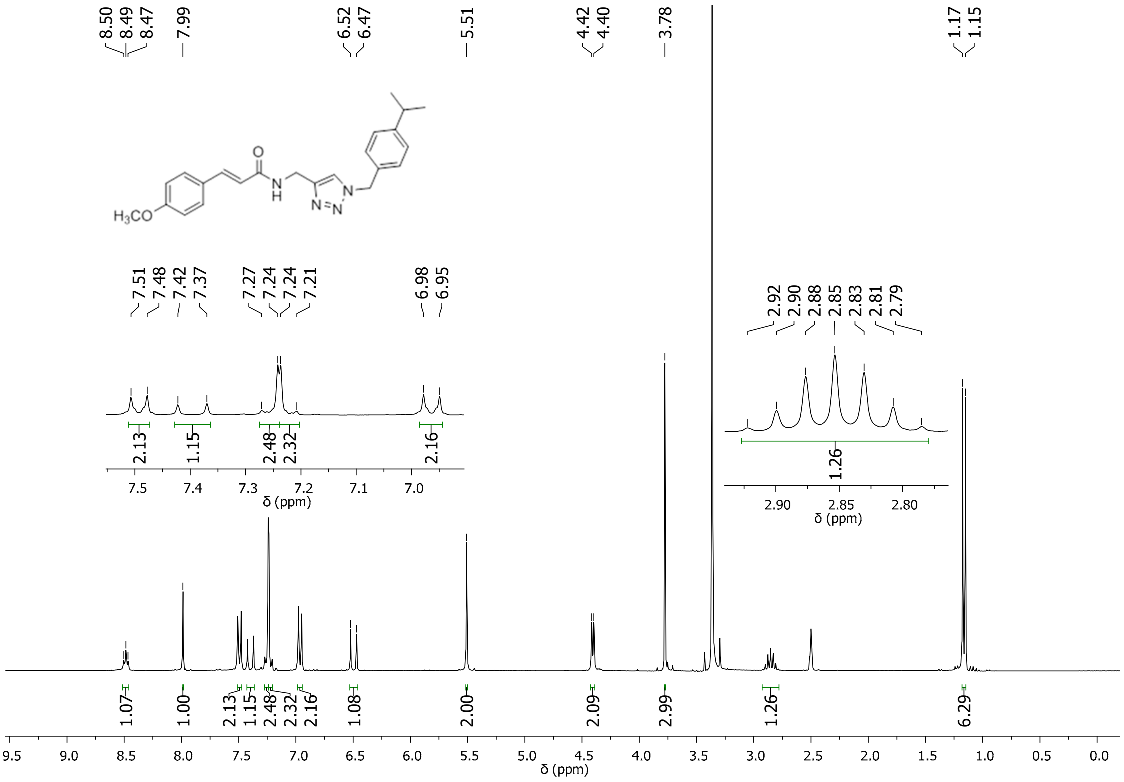

Figure S6 - ^{1}H NMR spectrum (400 MHz, DMSO-d_6) of **6**.

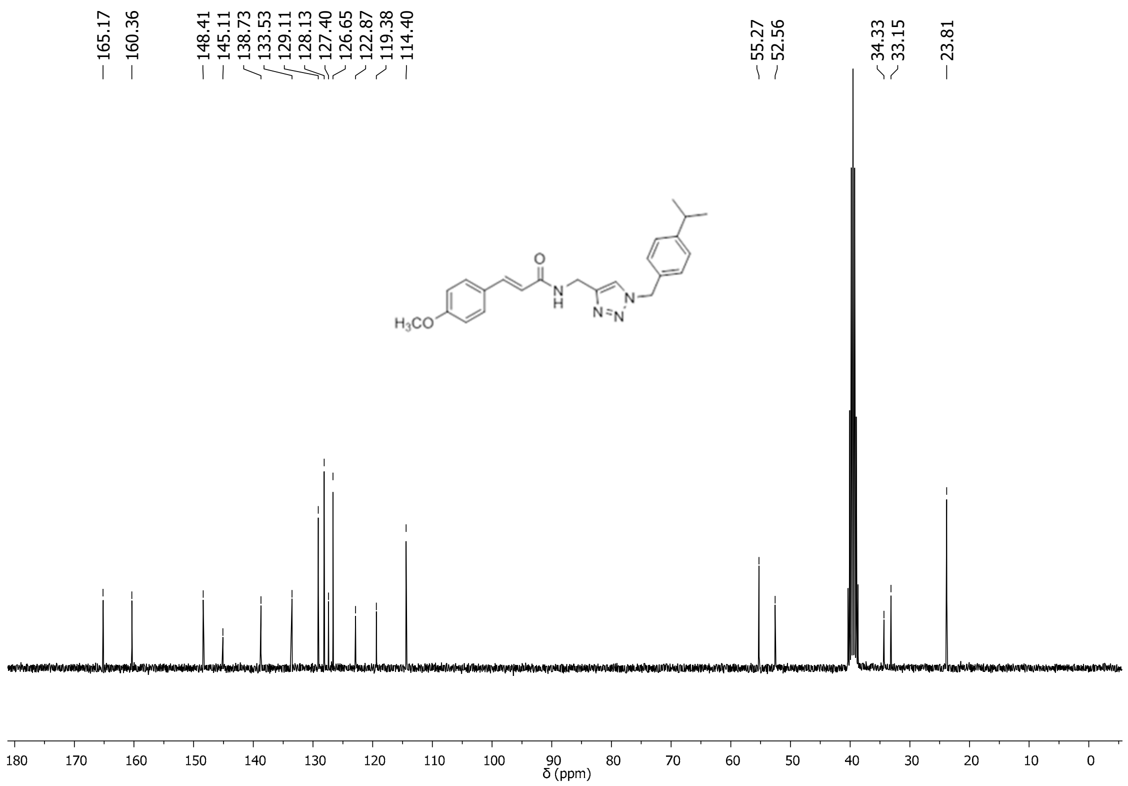

Figure S7 - ^{13}C NMR spectrum (100 MHz, DMSO-d_6) of **6**.

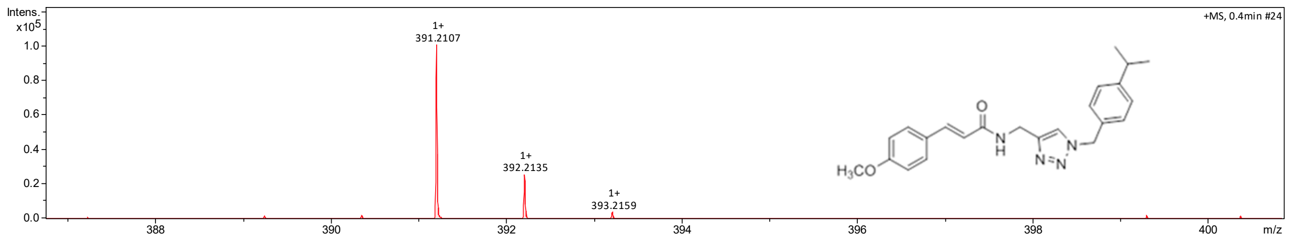

Figure S8 - HRMS spectrum of **6**.

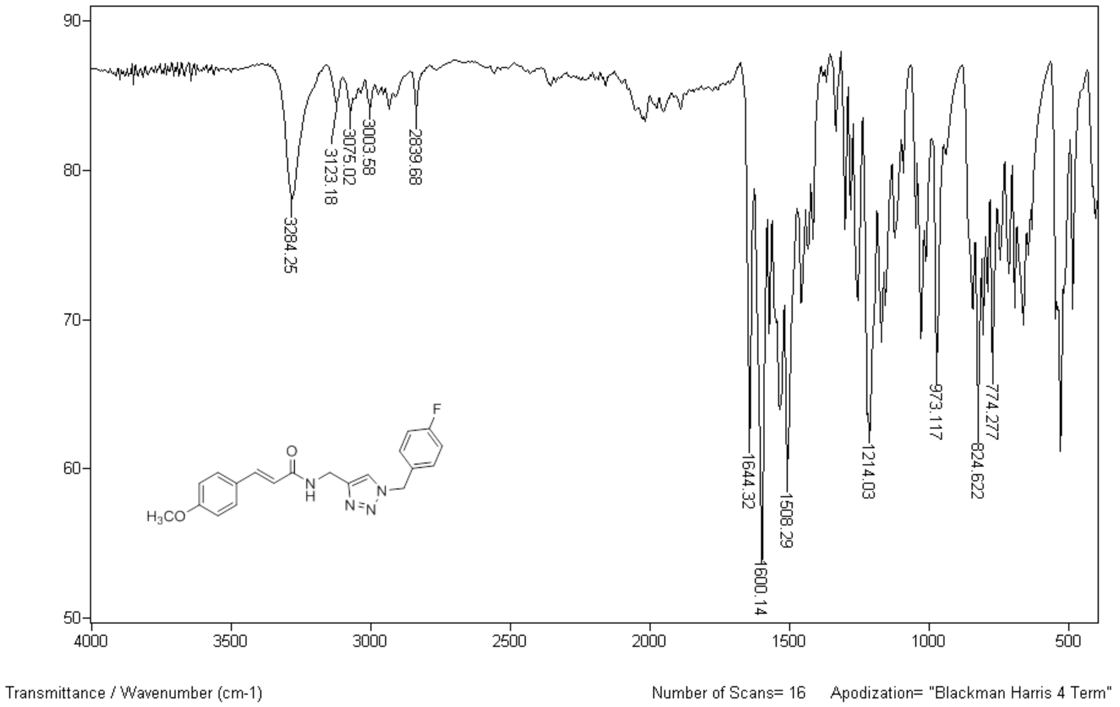

Figure S9 - IR spectrum (ATR) of **7**.

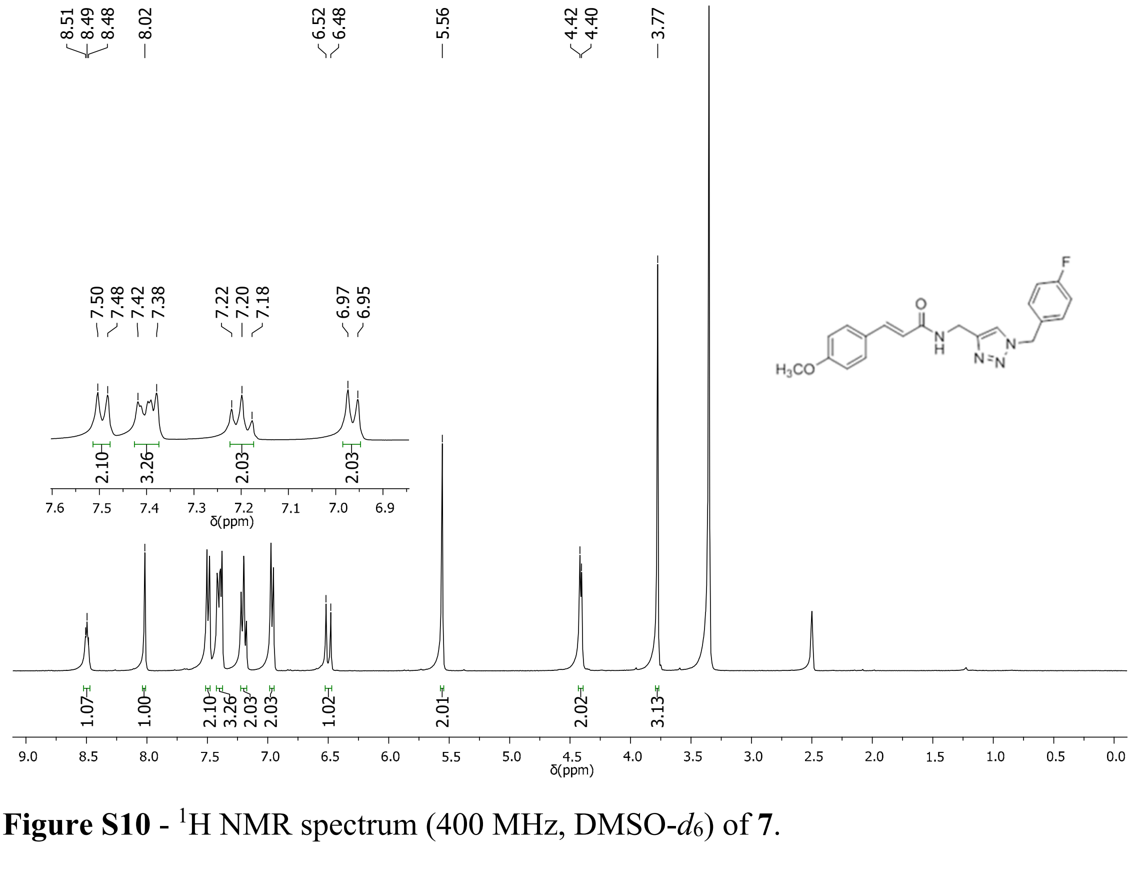

Figure S10 - ^{1}H NMR spectrum (400 MHz, DMSO-*d*6) of **7**.

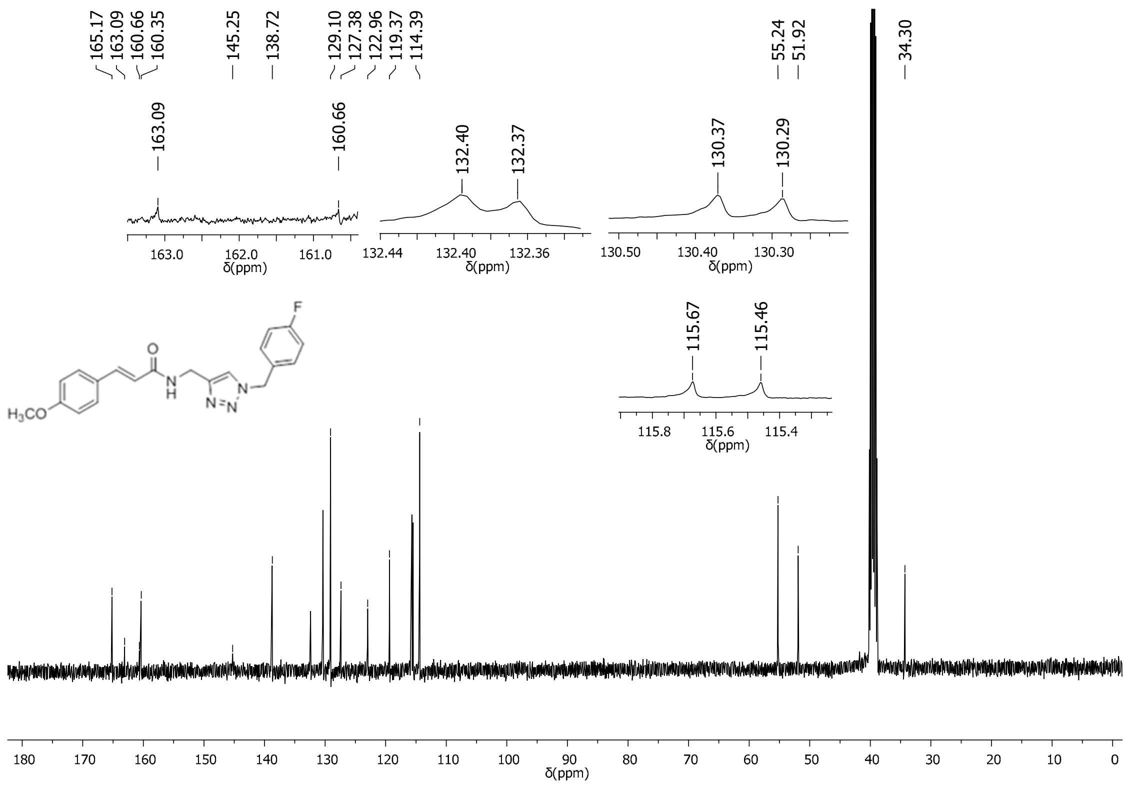

Figure S11 - ^{13}C NMR spectrum (100 MHz, DMSO-d_6) of **7**.

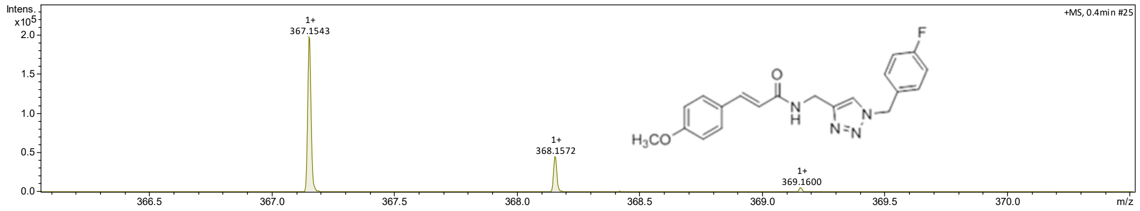

Figure S12 - HRMS spectrum of **7**.

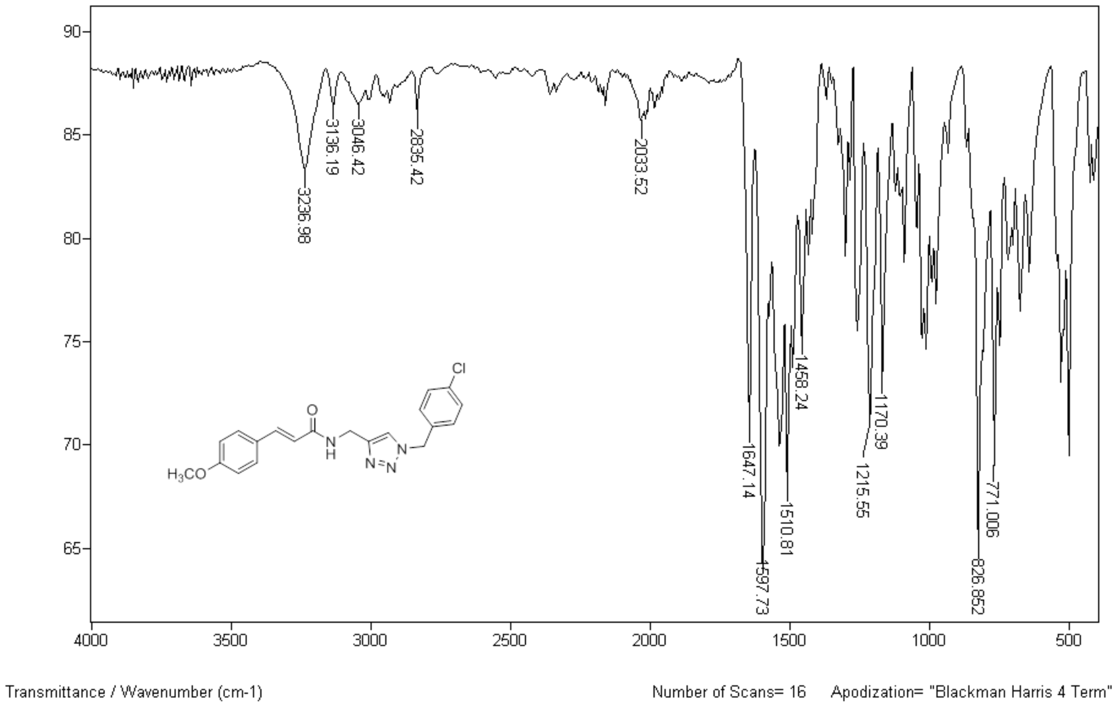

Figure S13 - IR spectrum (ATR) of **8**.

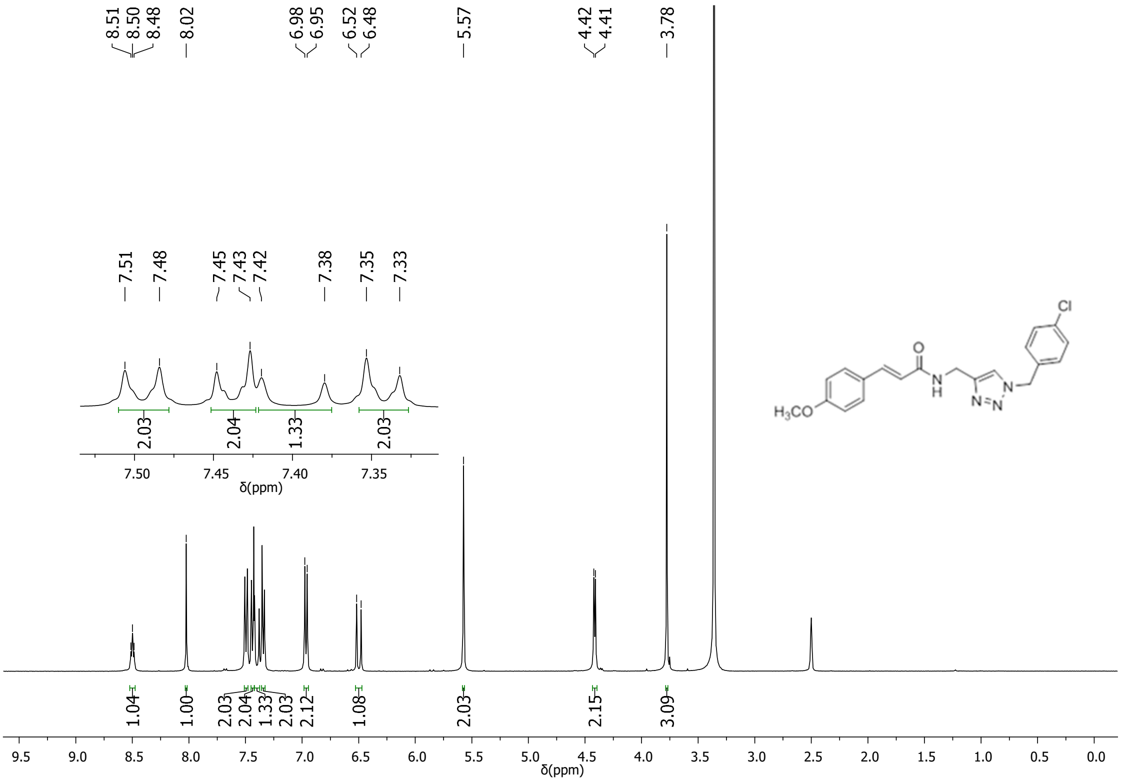

Figure S14 - ^{1}H NMR spectrum (400 MHz, DMSO-d_6) of **8**.

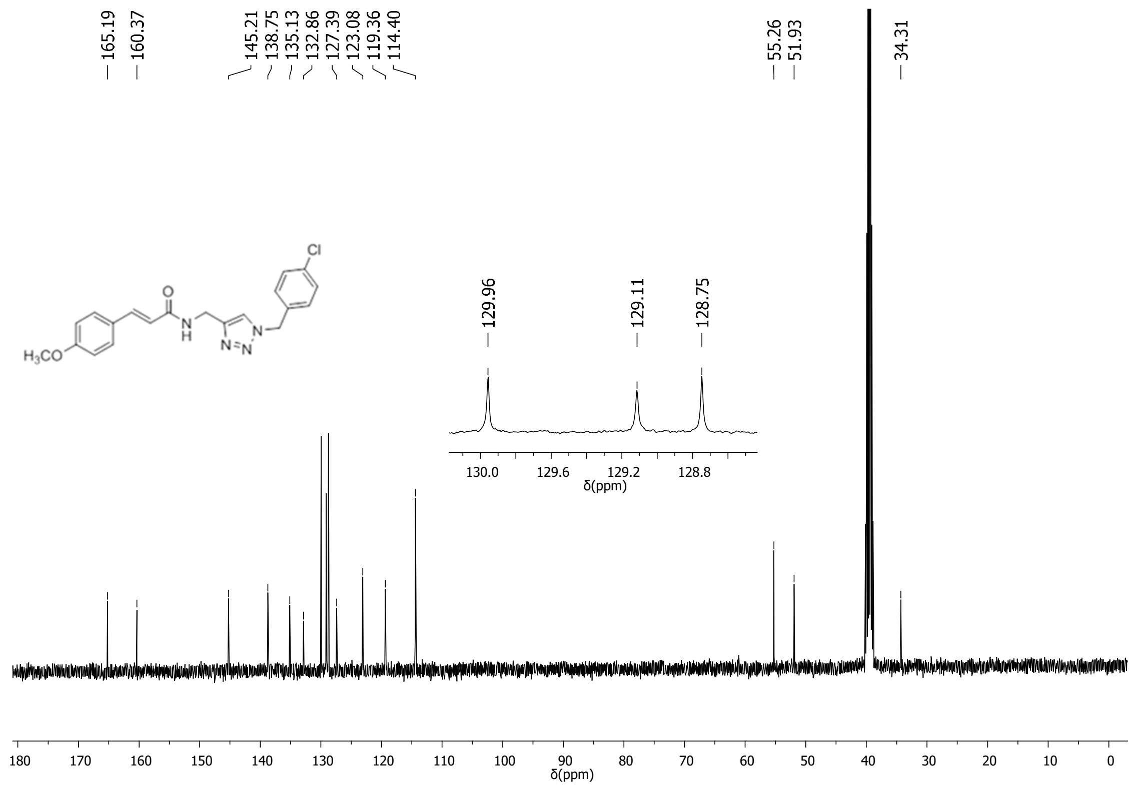

Figure S15 - ^{13}C NMR spectrum (100 MHz, DMSO-d_6) of **8**.

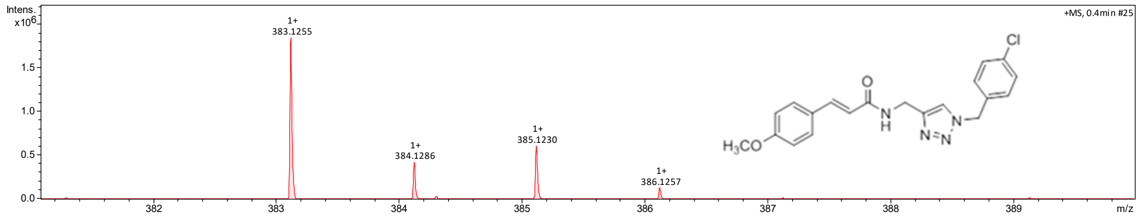

Figure S16 - HRMS spectrum of **8**.

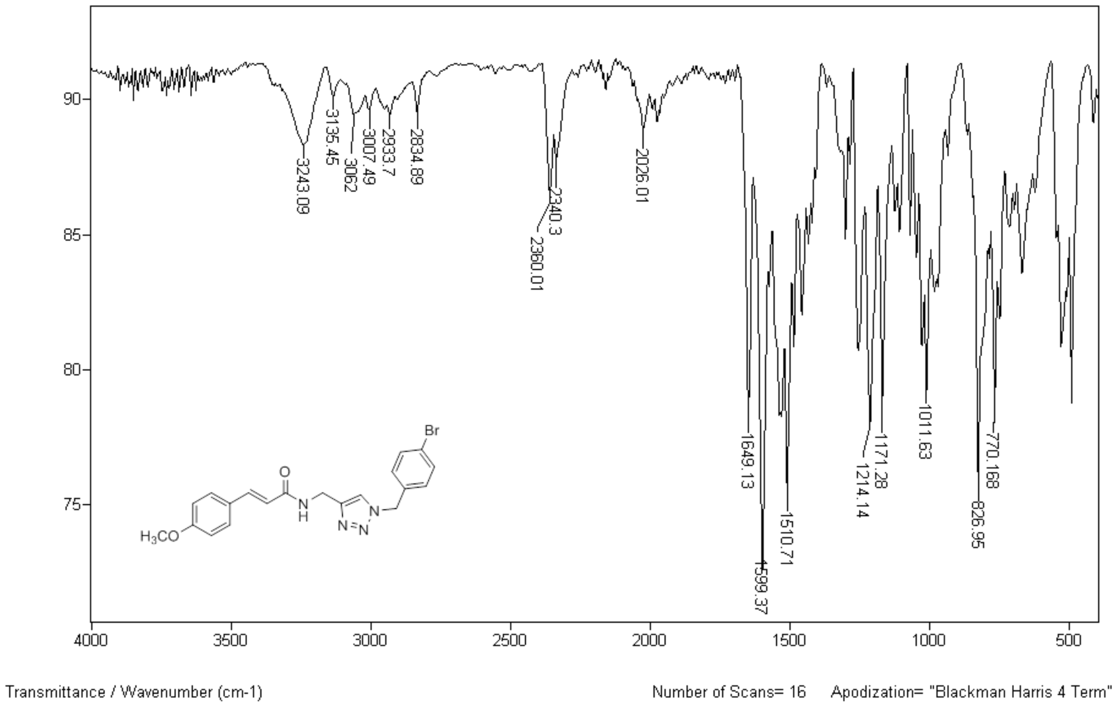

Figure S17 - IR spectrum (ATR) of **9**.

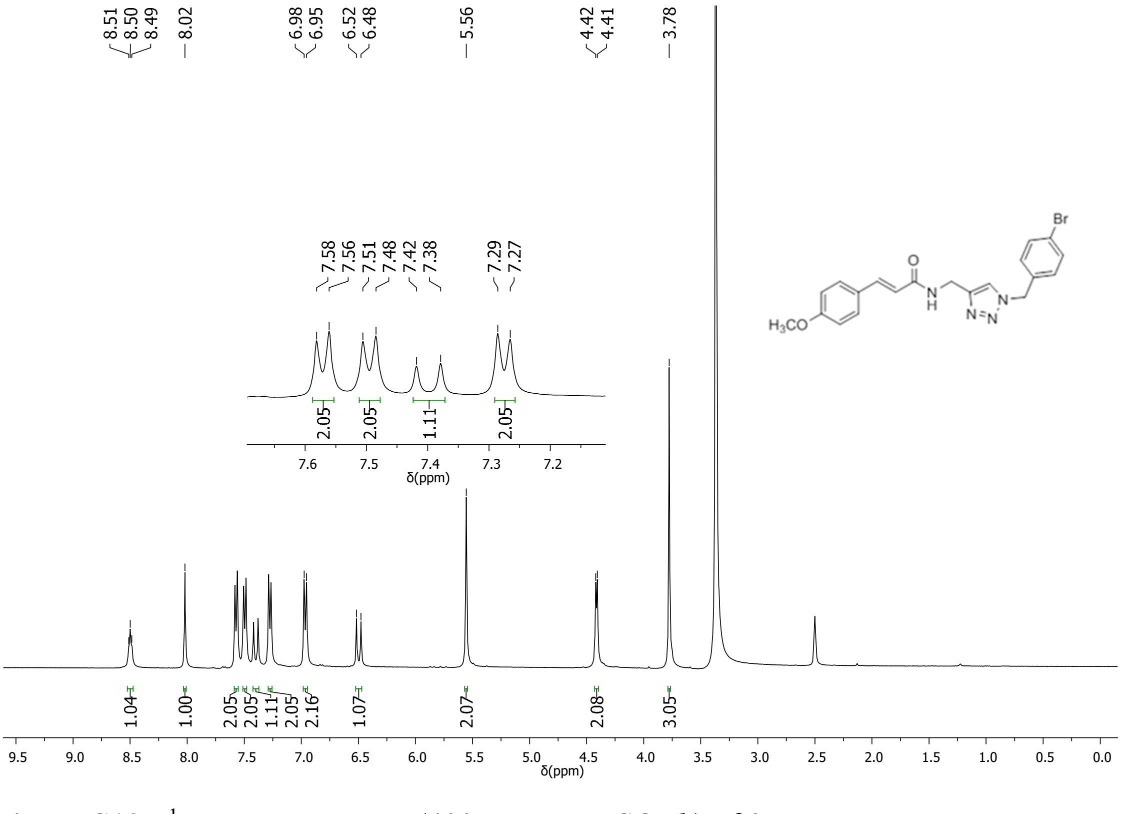

Figure S18 - ^{1}H NMR spectrum (400 MHz, DMSO-d_6) of **9**.

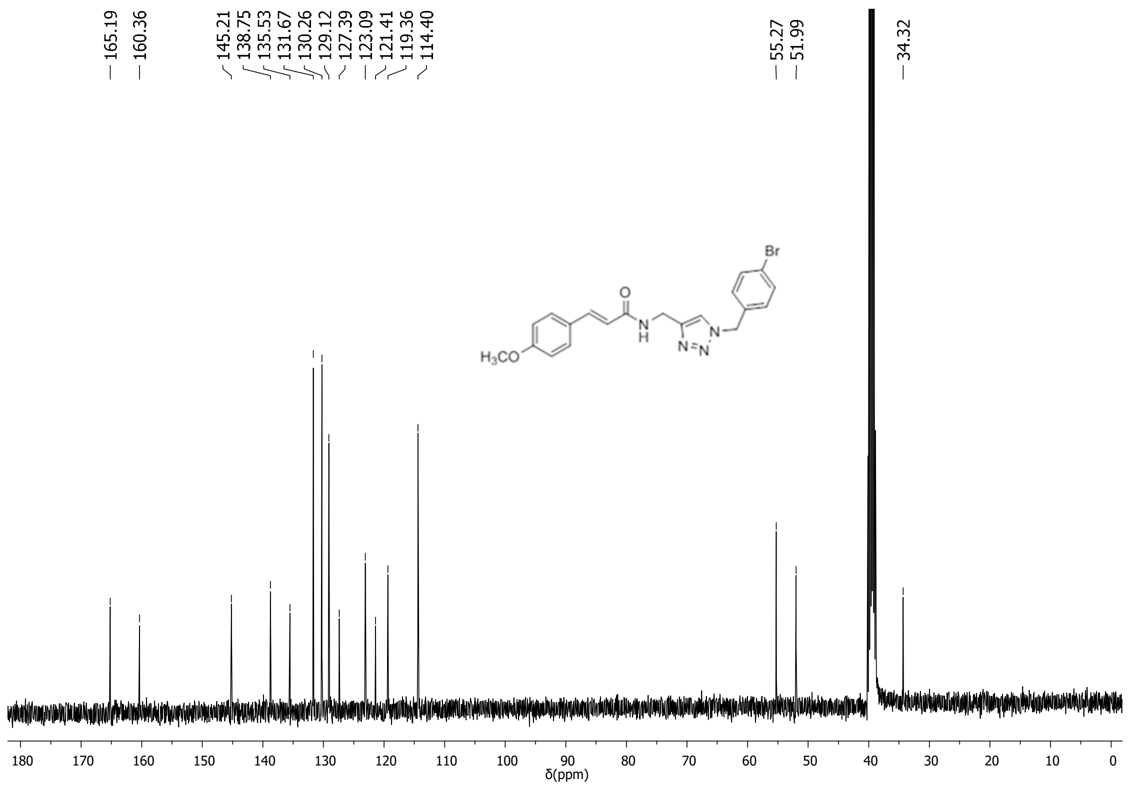

Figure S19 - ^{13}C NMR spectrum (100 MHz, DMSO-d_6) of **9**.

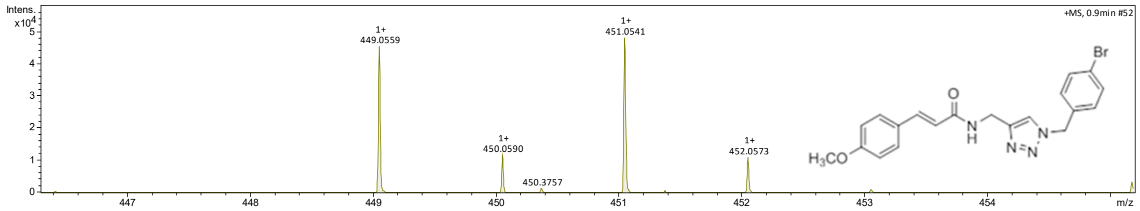

Figure S20 - HRMS spectrum of **9**.

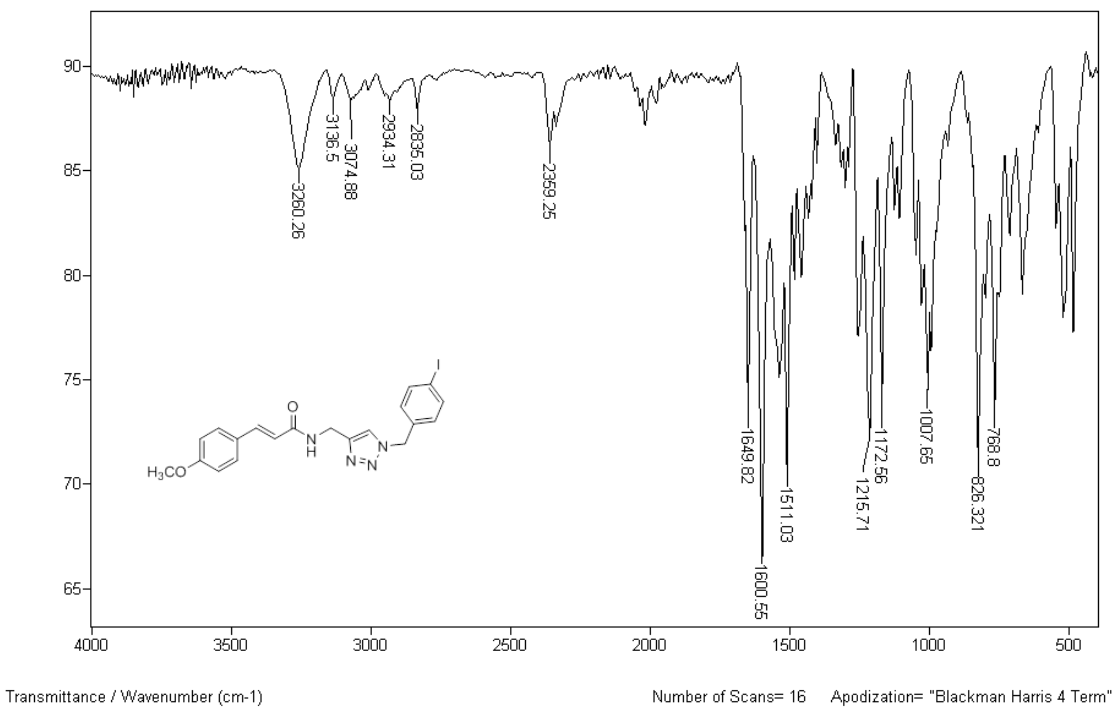

Figure S21 - IR spectrum (ATR) of **10**.

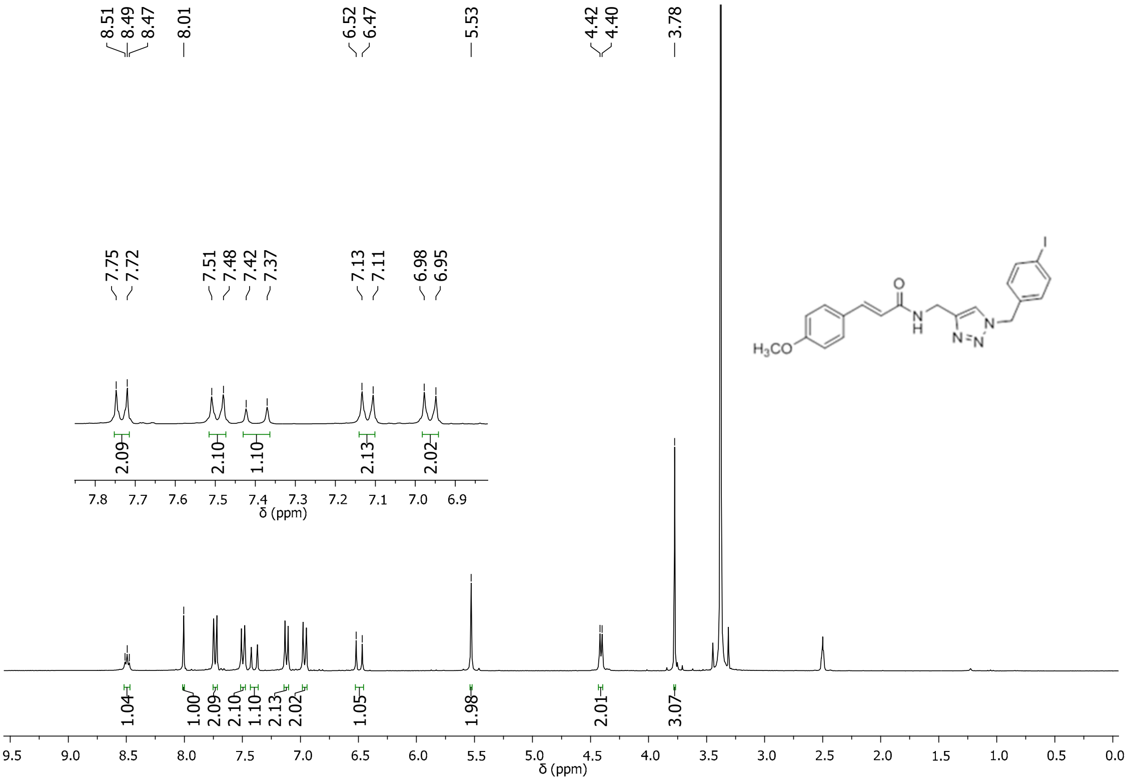

Figure S22 - ^{1}H NMR spectrum (400 MHz, DMSO-d_6) of **10**.

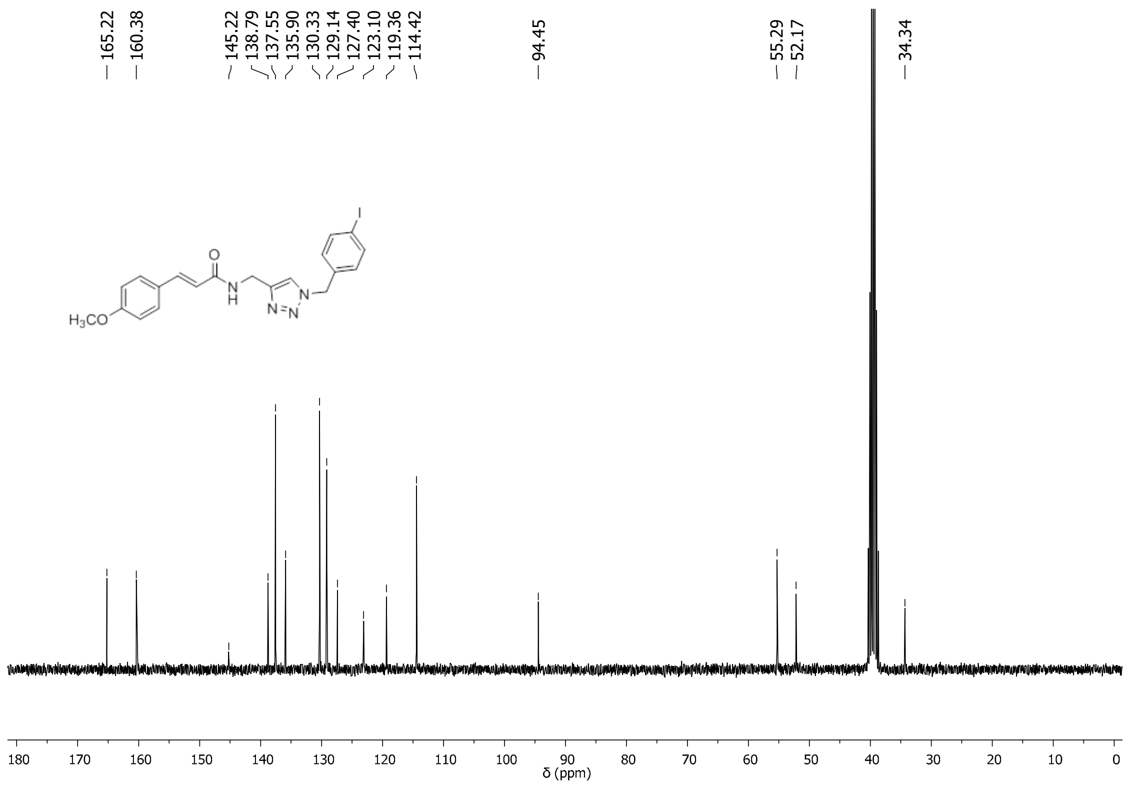

Figure S23 - ^{13}C NMR spectrum (100 MHz, DMSO-d_6) of **10**.

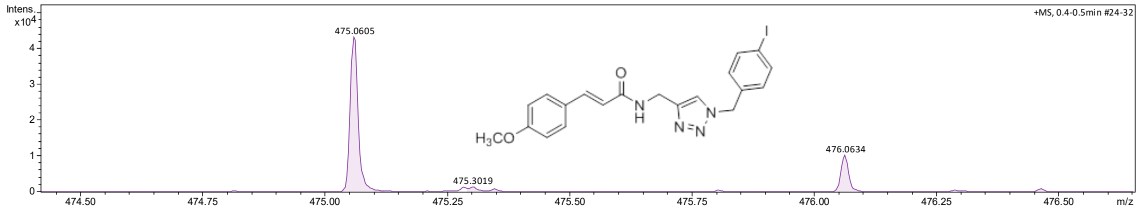

Figure S24 - HRMS spectrum of **10**.

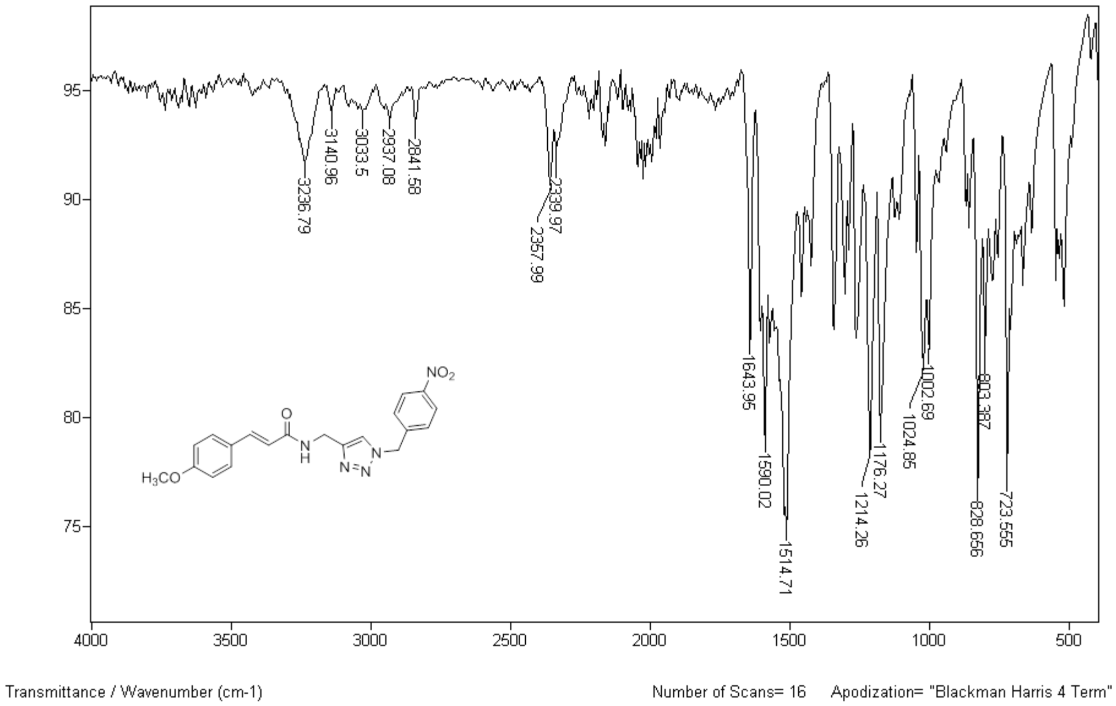

Figure S25 - IR spectrum (ATR) of **11**.

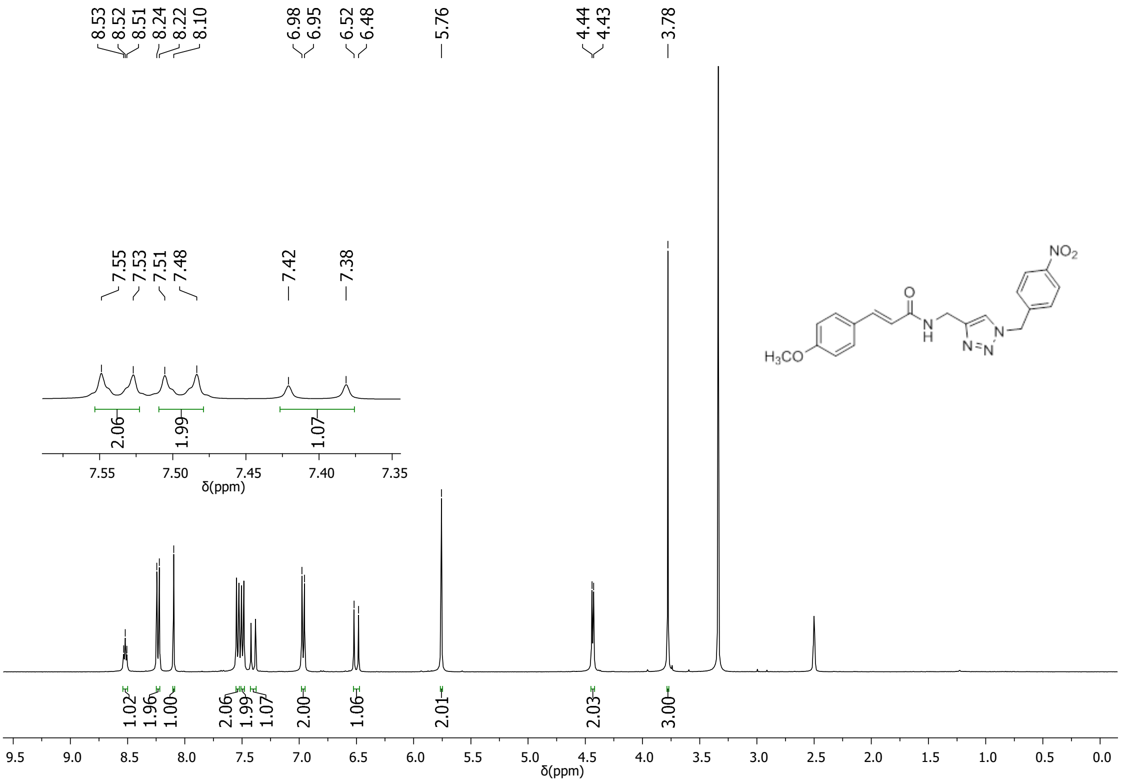

Figure S26 - ^{1}H NMR spectrum (400 MHz, DMSO-d_6) of **11**.

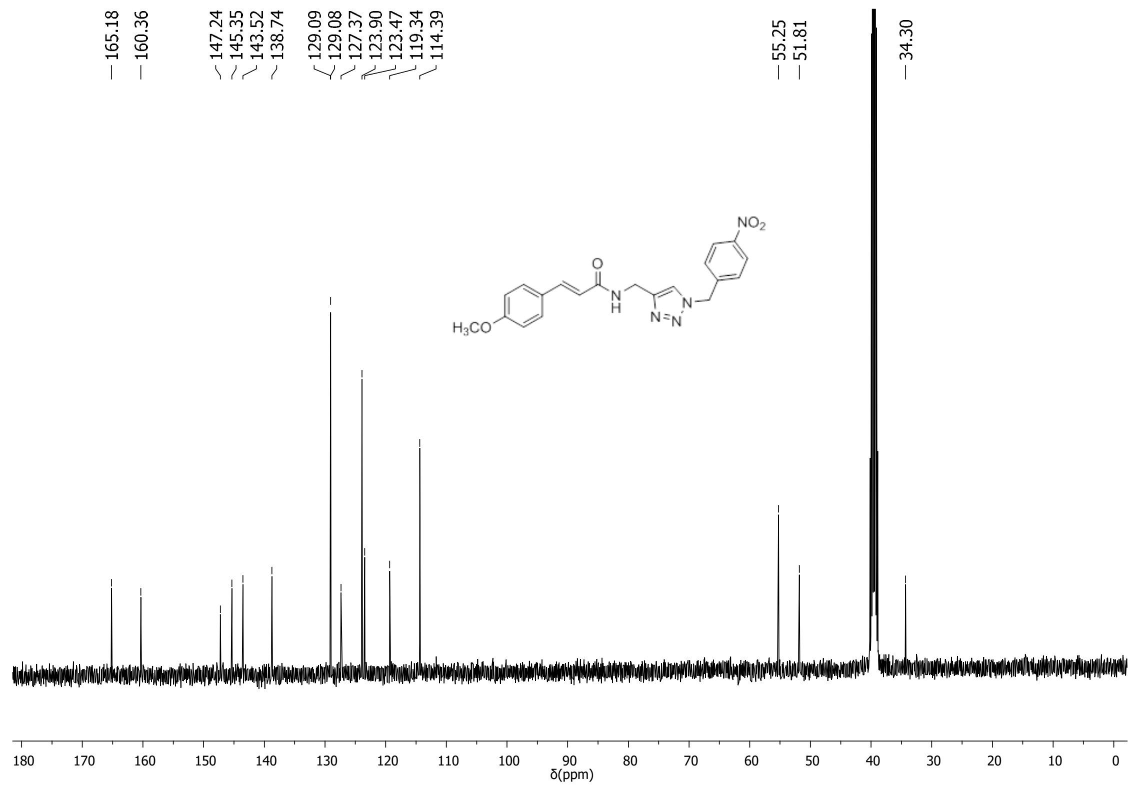

Figure S27 - ^{13}C NMR spectrum (100 MHz, DMSO-d_6) of **11**.

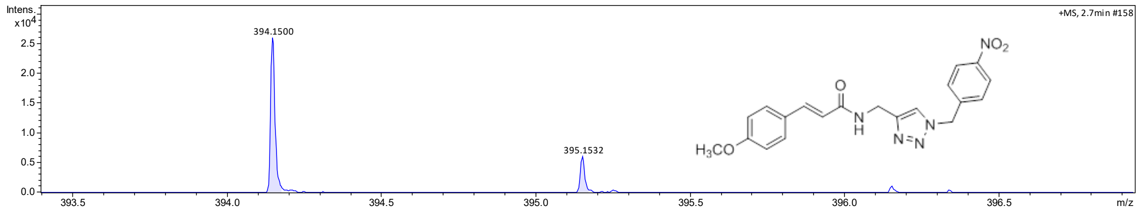

Figure S28 - HRMS spectrum of **11**.

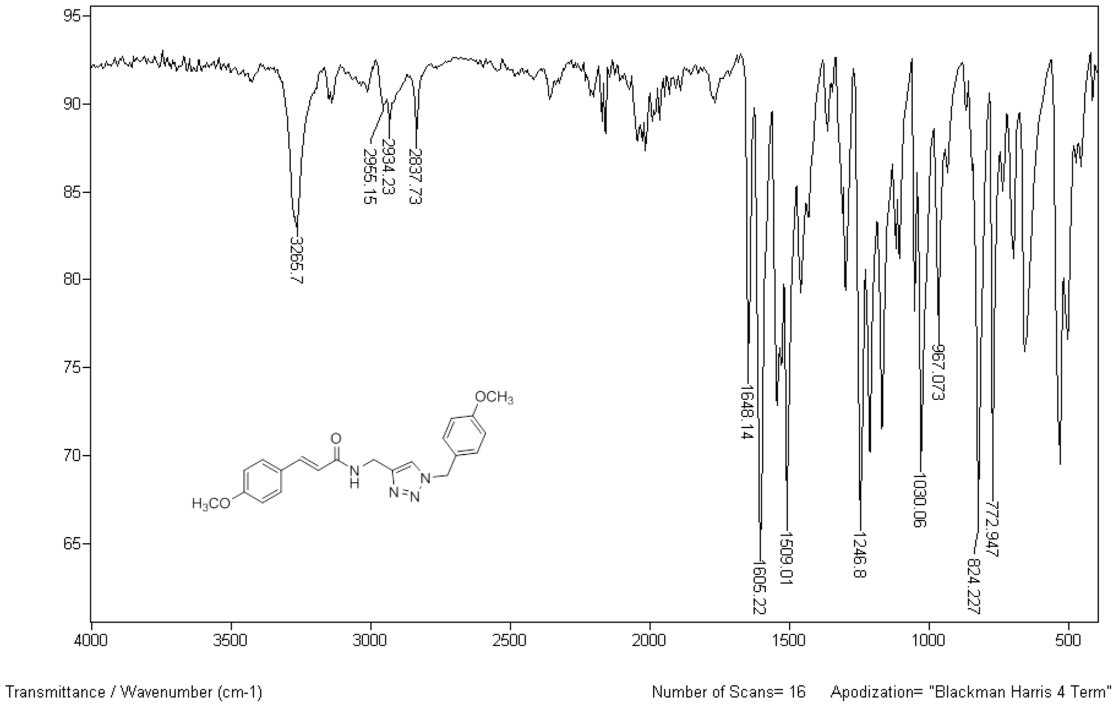

Figure S29 - IR spectrum (ATR) of **12**.

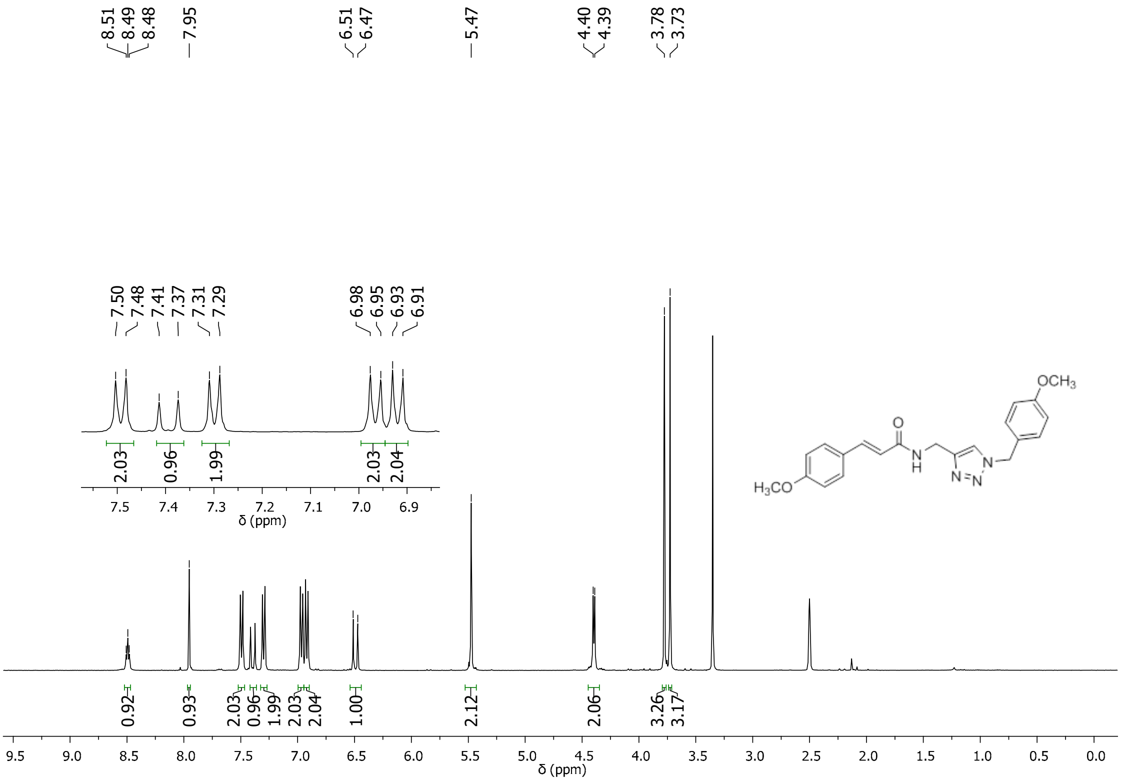

Figure S30 - ^{1}H NMR spectrum (400 MHz, DMSO-d_6) of **12**.

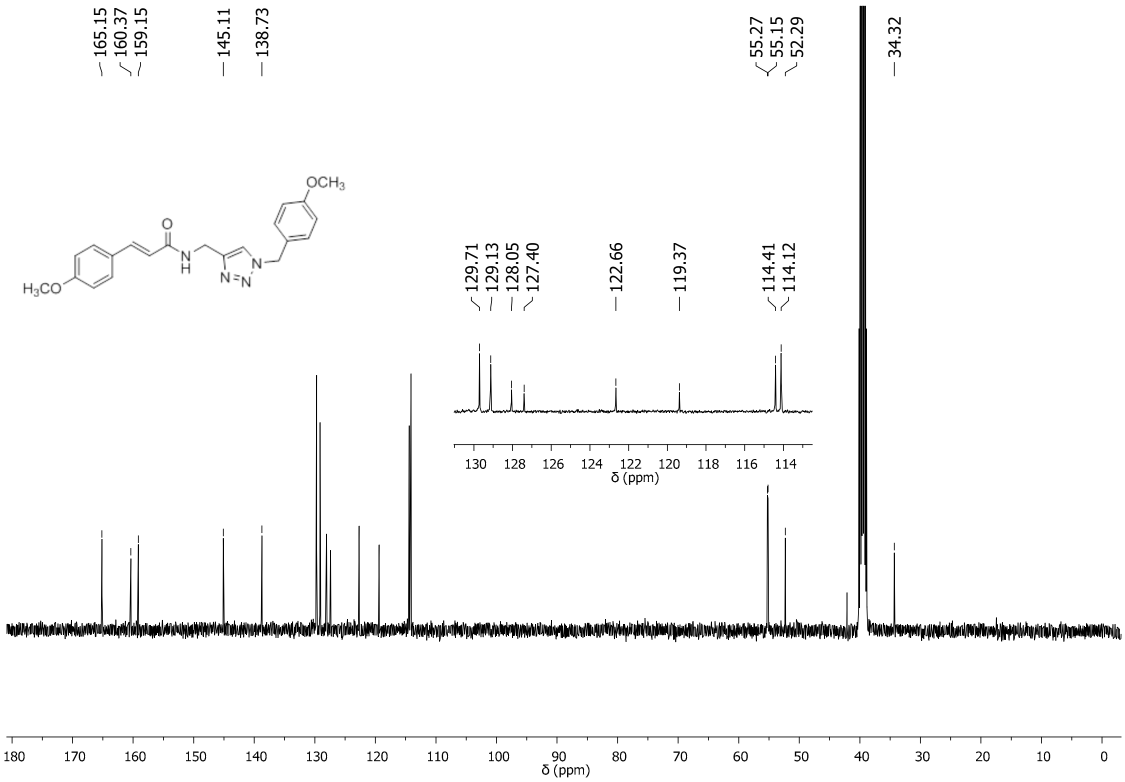

Figure S31 - ^{13}C NMR spectrum (100 MHz, DMSO-d_6) of **12**.

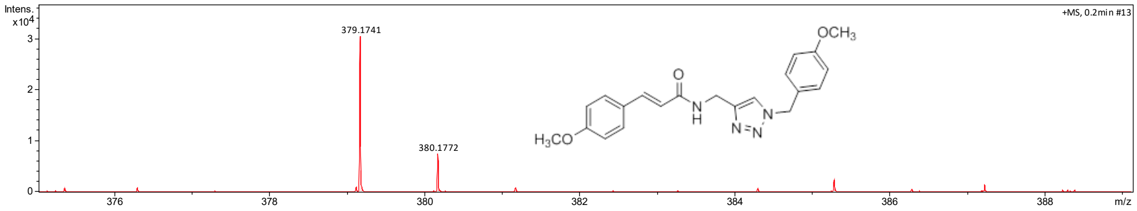

Figure S32 - HRMS spectrum of **12**.

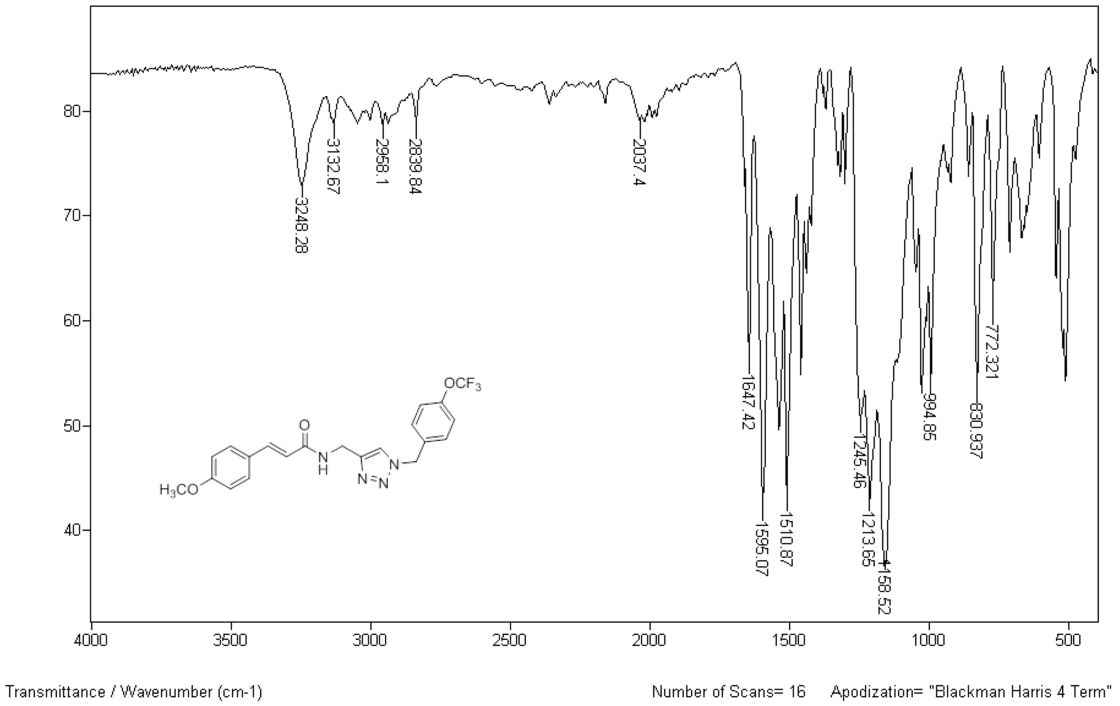

Figure S33 - IR spectrum (ATR) of **13**.

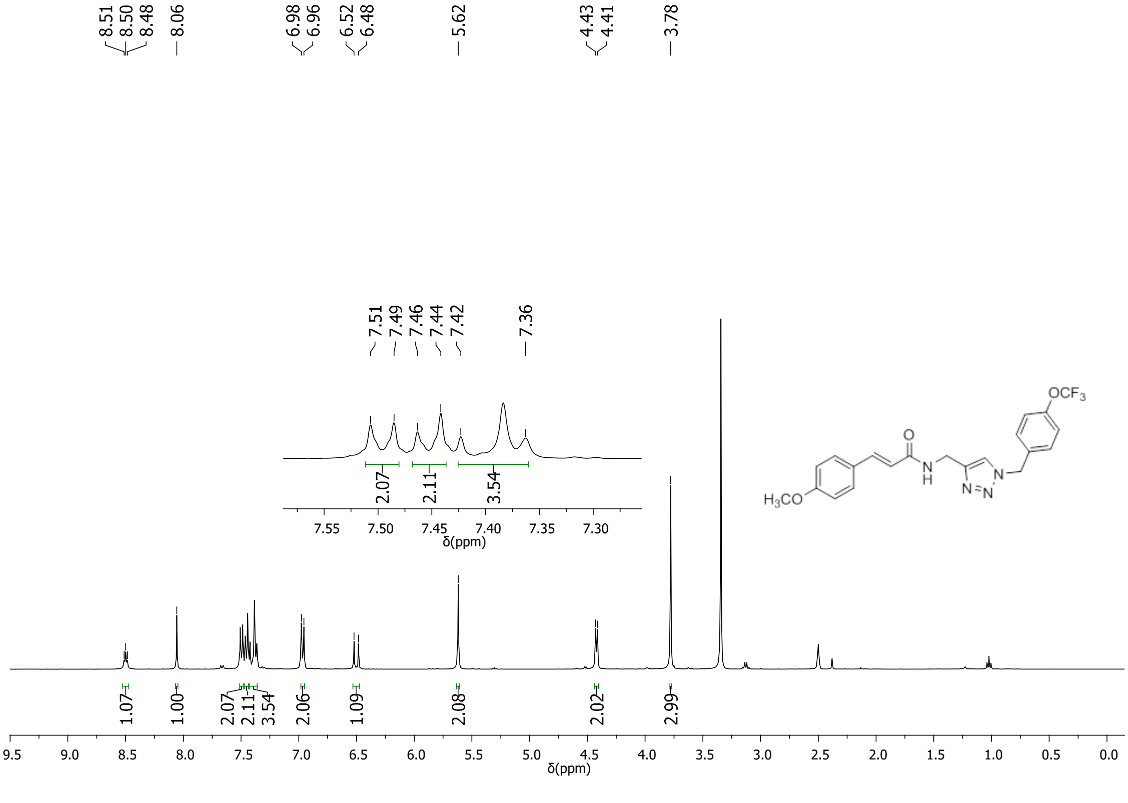

Figure S34 - ^{1}H NMR spectrum (400 MHz, DMSO-d_6) of **13**.

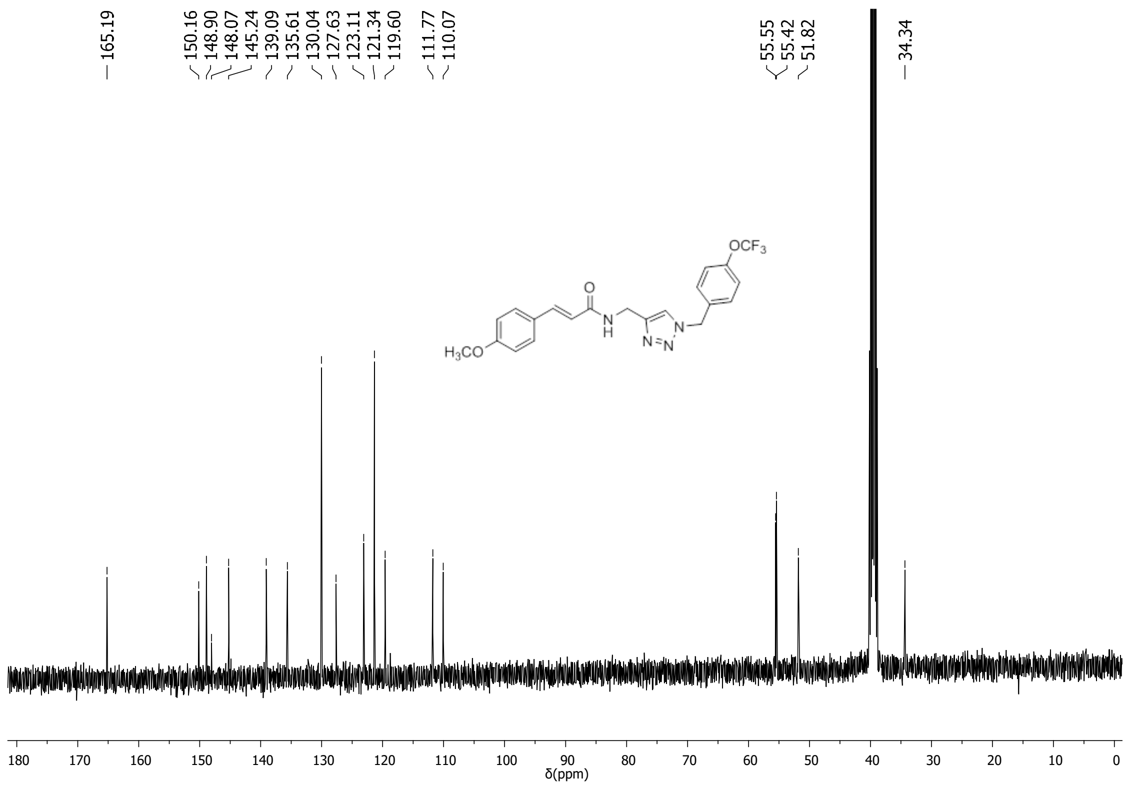

Figure S35 - ^{13}C NMR spectrum (100 MHz, DMSO-d_6) of **13**.

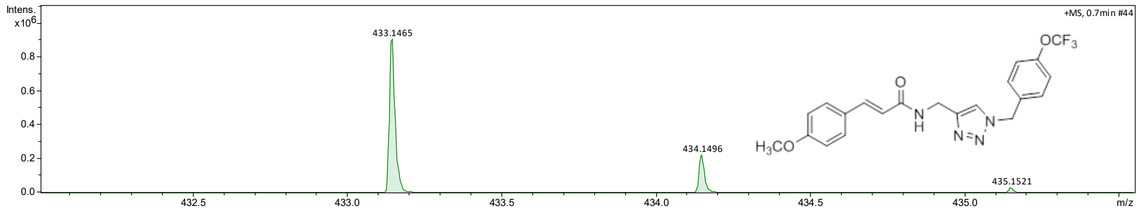

Figure S36 - HRMS spectrum of **13**.

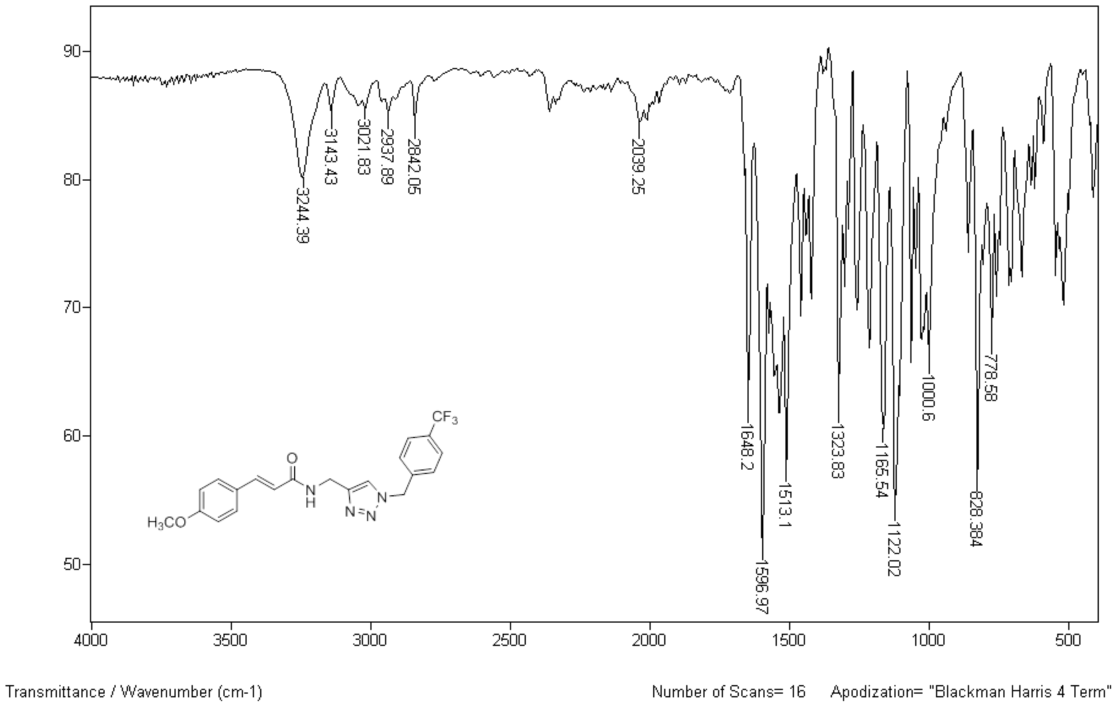

Figure S37 - IR spectrum (ATR) of **14**.

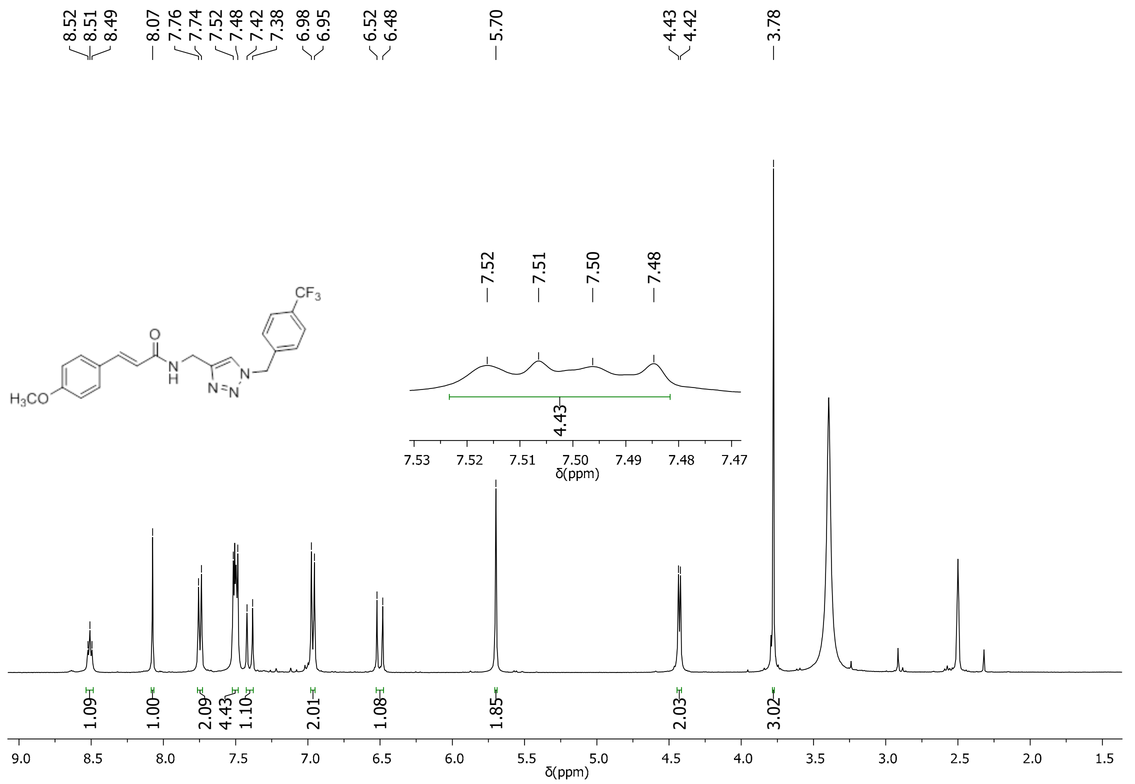

Figure S38 - ^{1}H NMR spectrum (400 MHz, DMSO-d_6) of **14**.

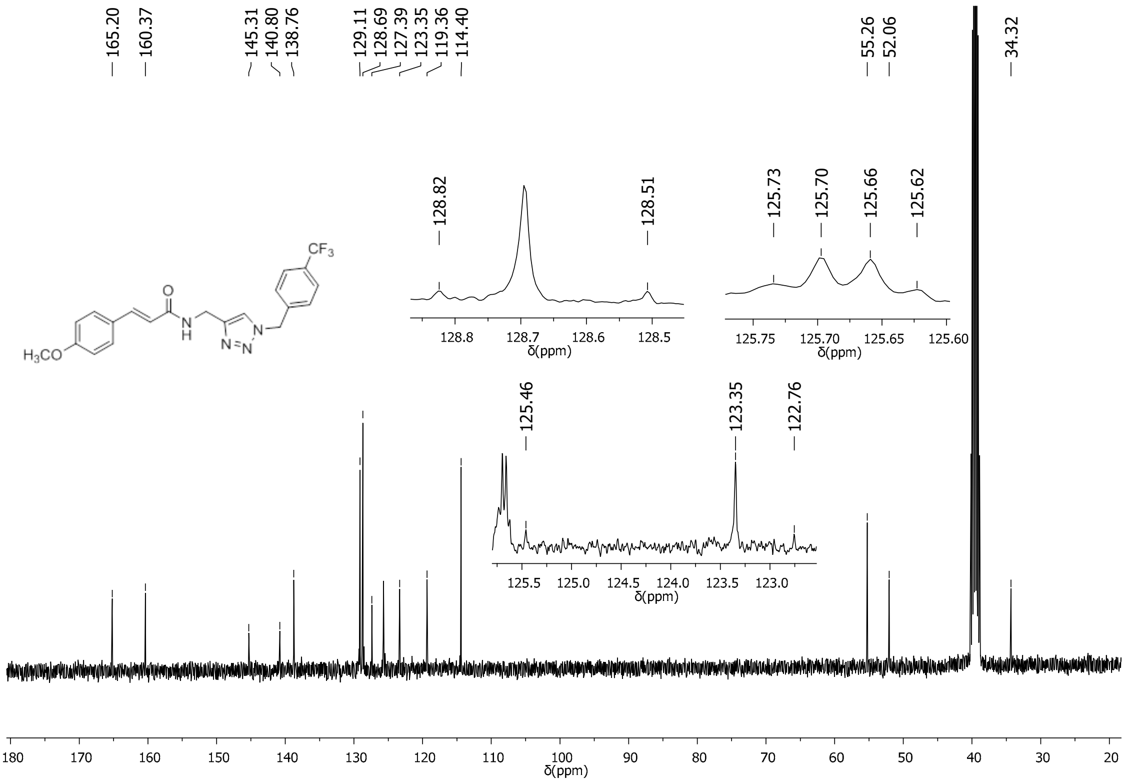

Figure S39 - ^{13}C NMR spectrum (100 MHz, DMSO-d_6) of **14**.

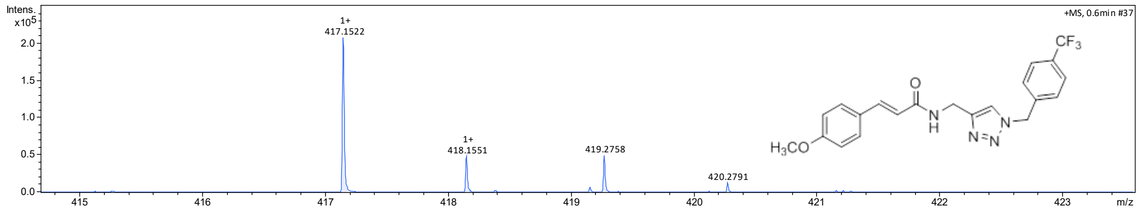

Figure S40 - HRMS spectrum of **14**.

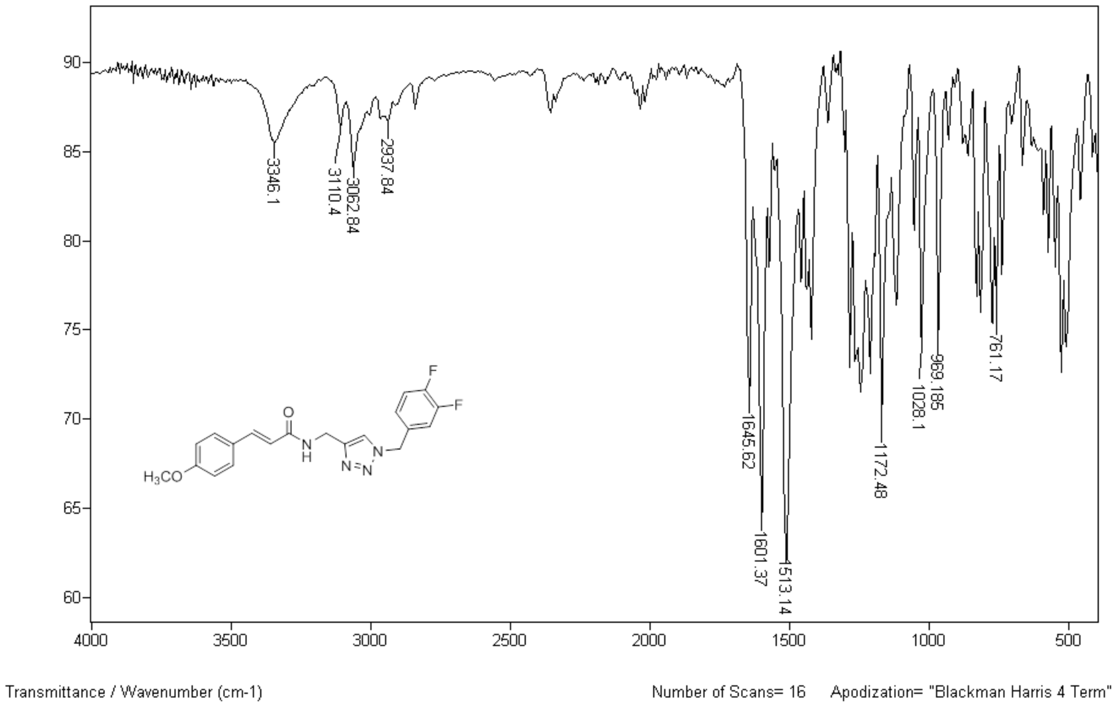

Figure S41 - IR spectrum (ATR) of **15**.

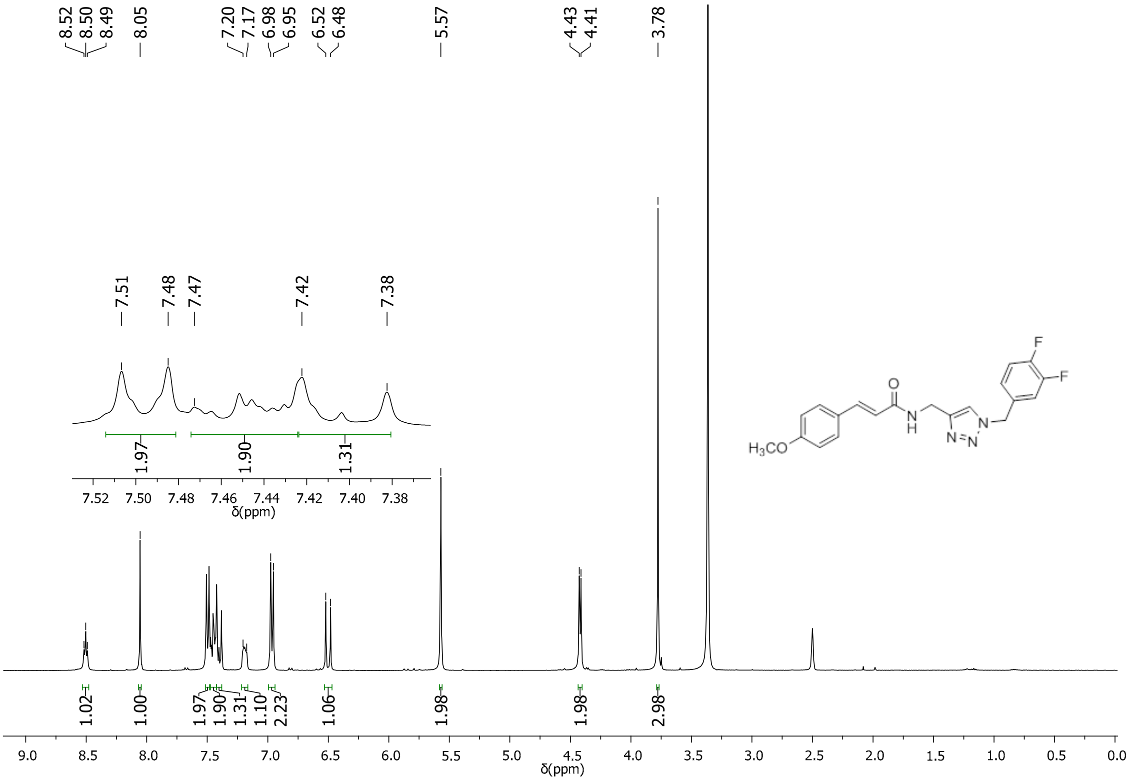

Figure S42 - ^{1}H NMR spectrum (400 MHz, DMSO-d_6) of **15**.

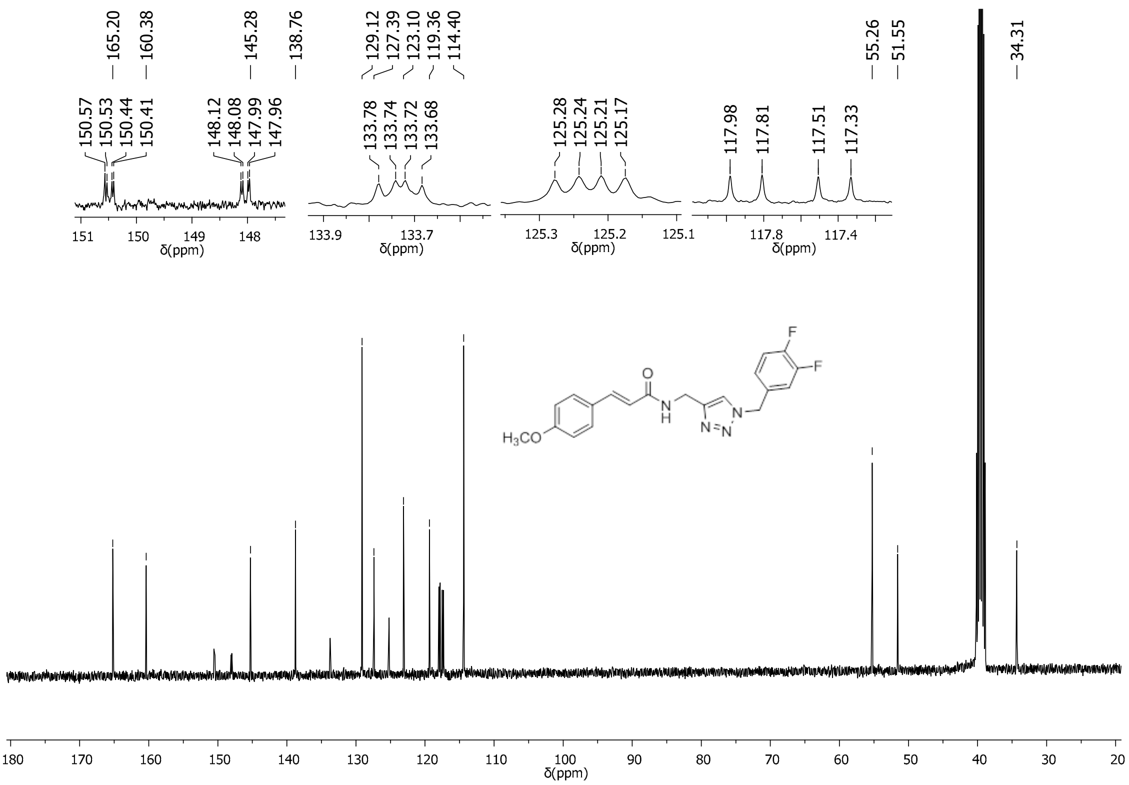

Figure S43 - ^{13}C NMR spectrum (100 MHz, DMSO-d_6) of **15**.

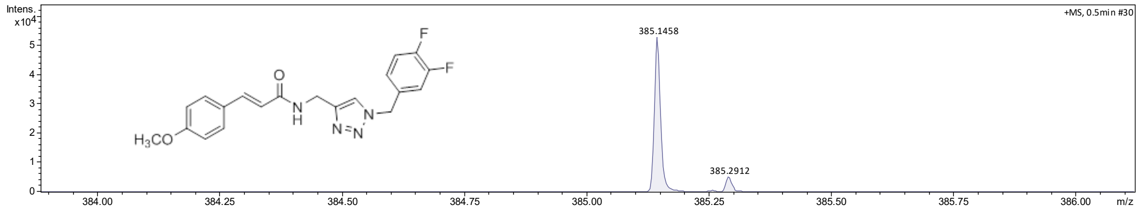

Figure S44 - HRMS spectrum of **15**.

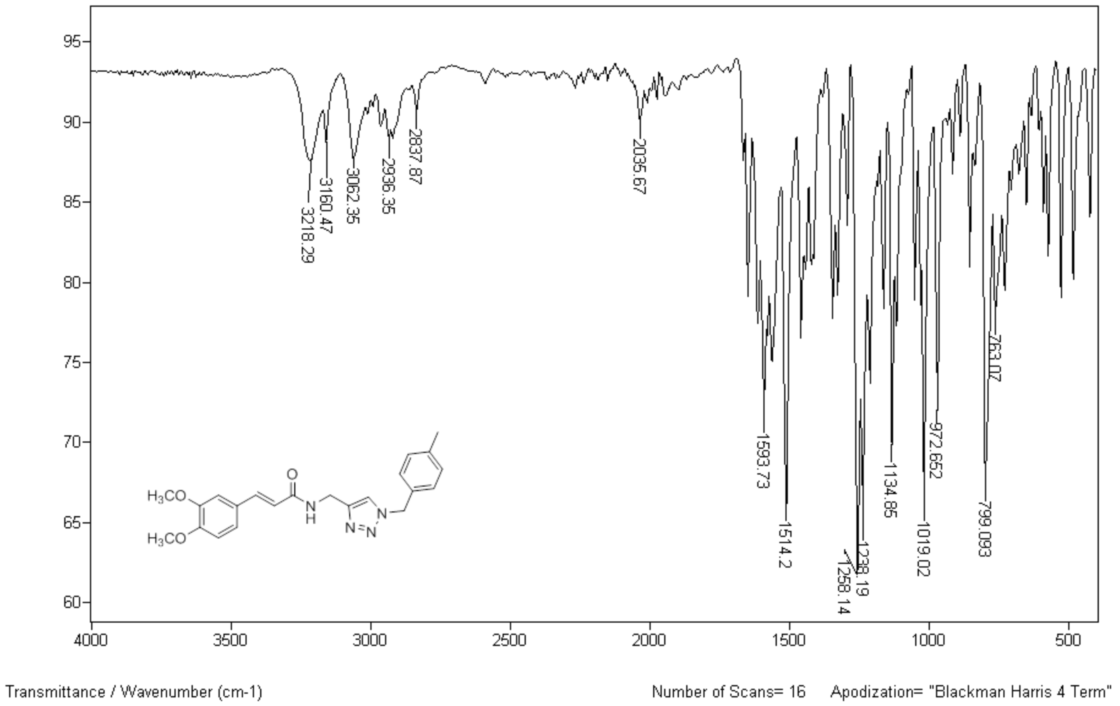

Figure S45 - IR spectrum (ATR) of **16**.

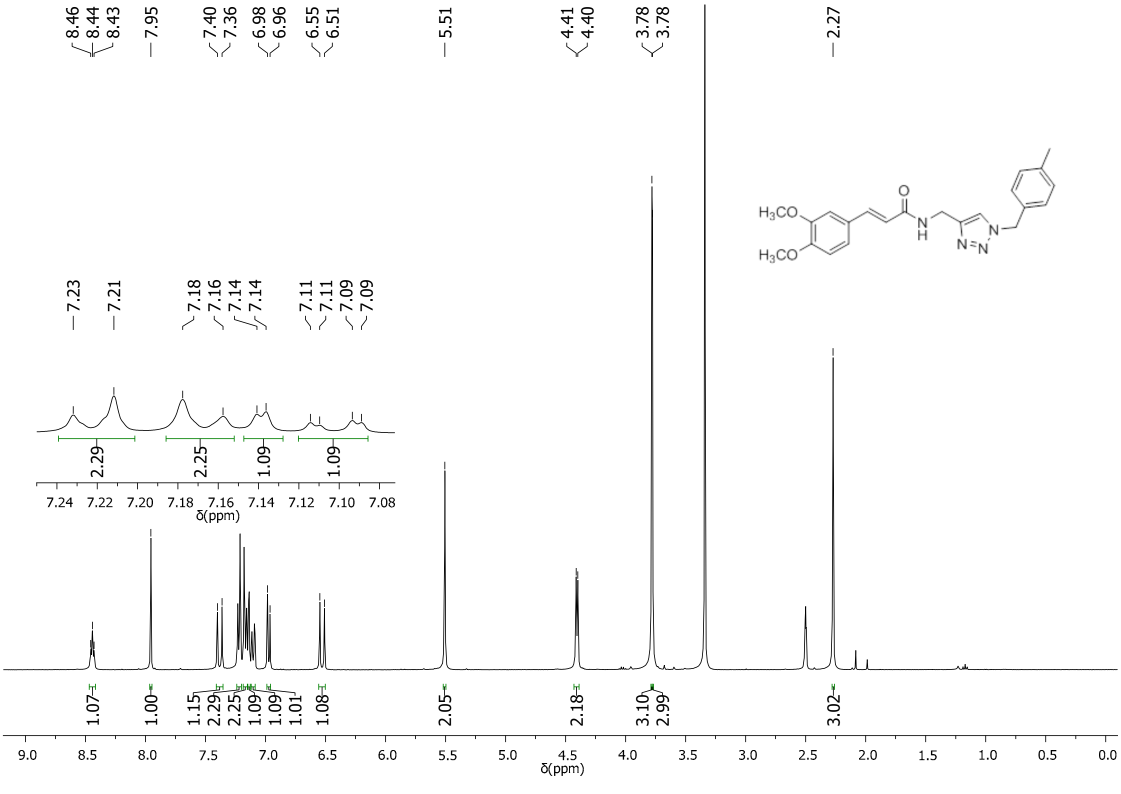

Figure S46 - ^{1}H NMR spectrum (400 MHz, DMSO-d_6) of **16**.

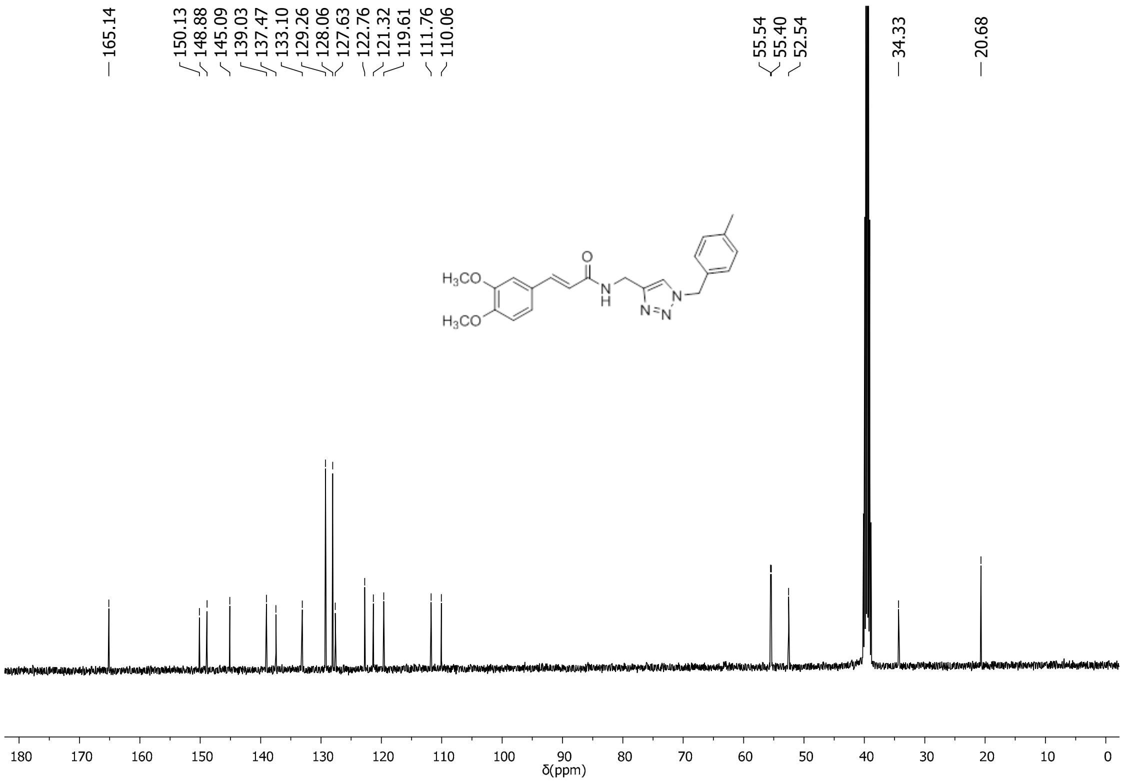

Figure S47 - ^{13}C NMR spectrum (100 MHz, DMSO-d_6) of **16**.

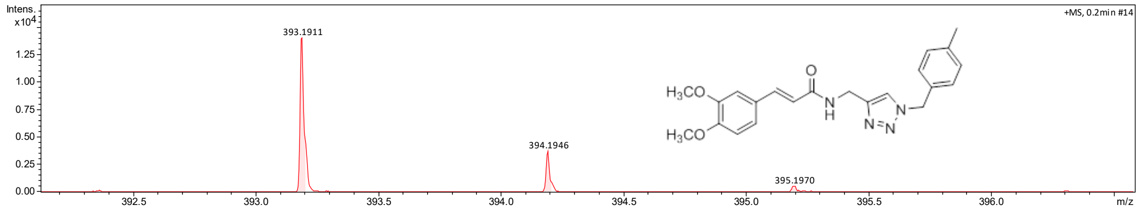

Figure S48 - HRMS spectrum of **16**.

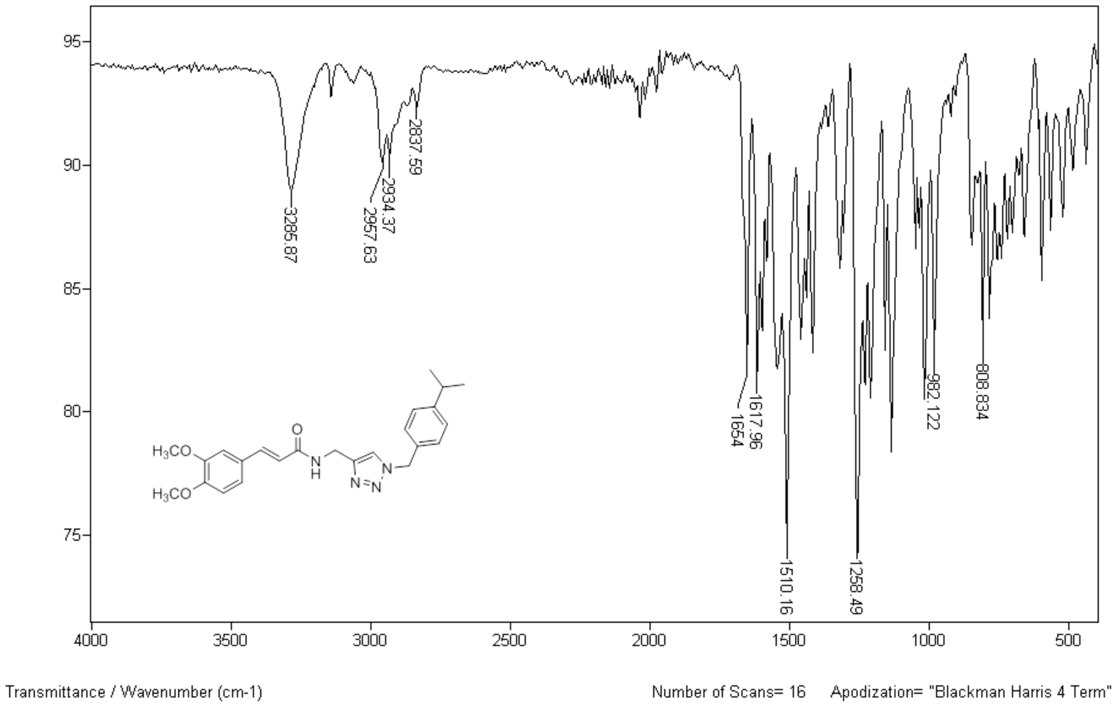

Figure S49 - IR spectrum (ATR) of **17**.

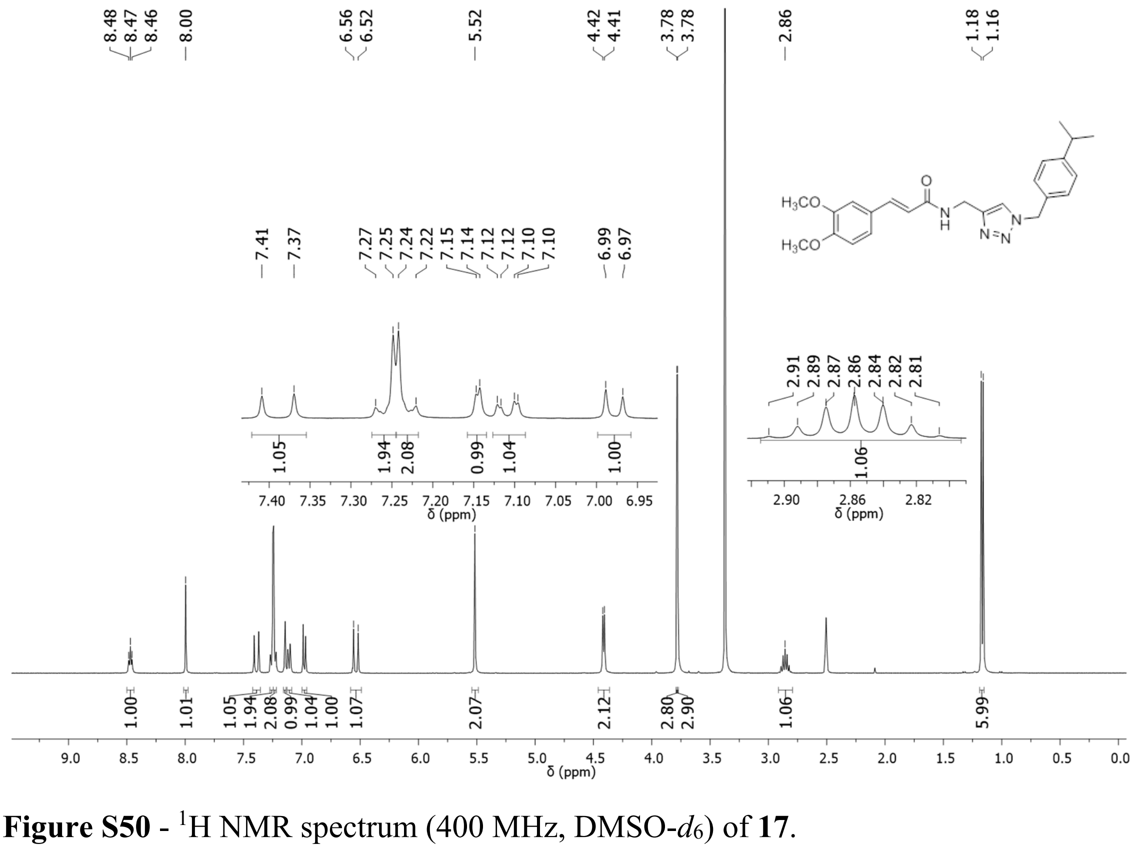

Figure S50 - ^{1}H NMR spectrum (400 MHz, DMSO-d_6) of **17**.

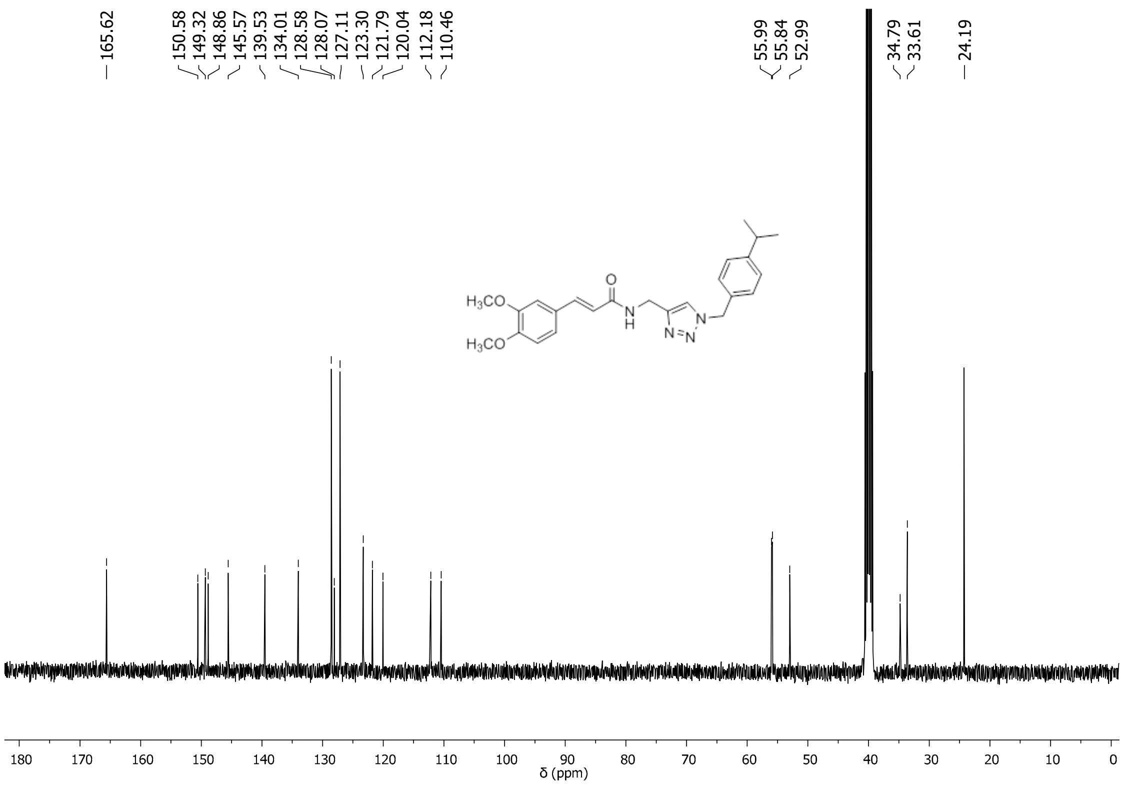

Figure S51 - ^{13}C NMR spectrum (100 MHz, DMSO-d_6) of **17**.

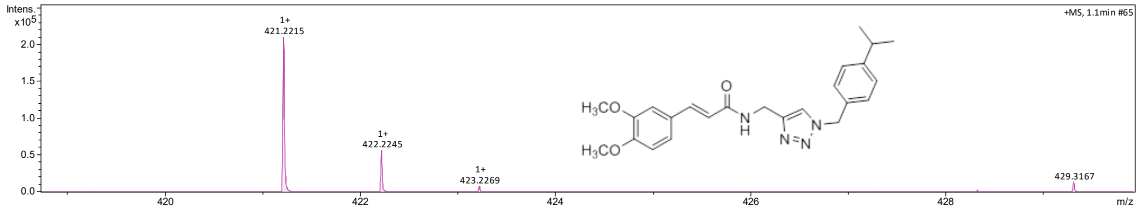

Figure S52 - HRMS spectrum of **17**.

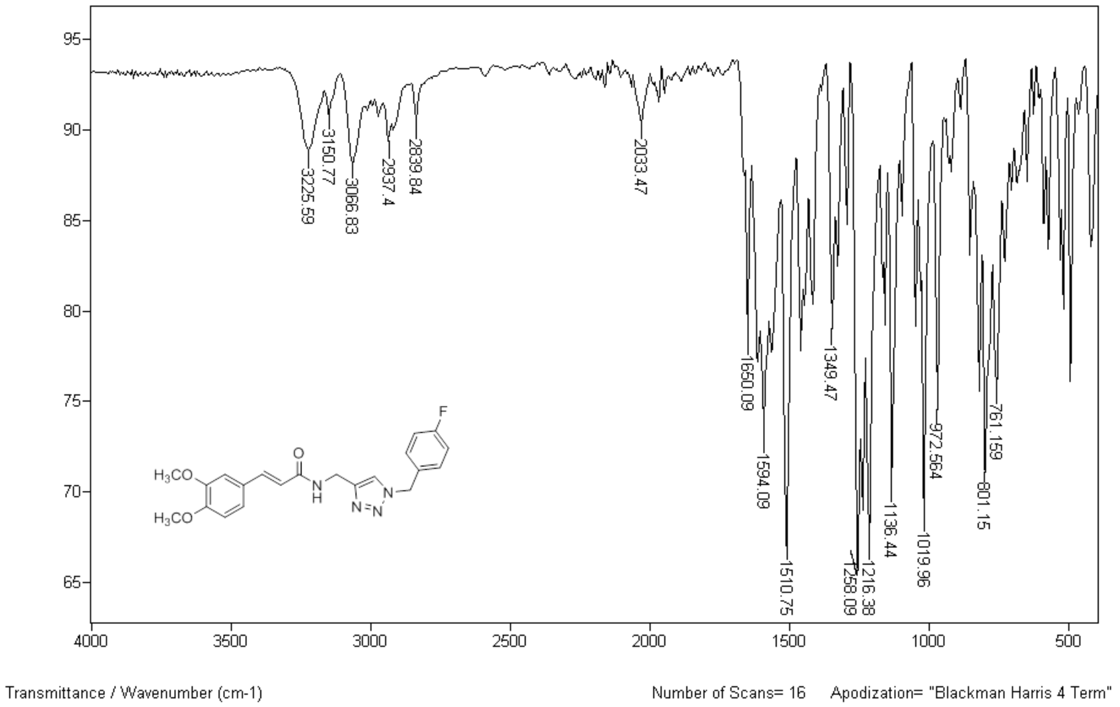

Figure S53 - IR spectrum (ATR) of **18**.

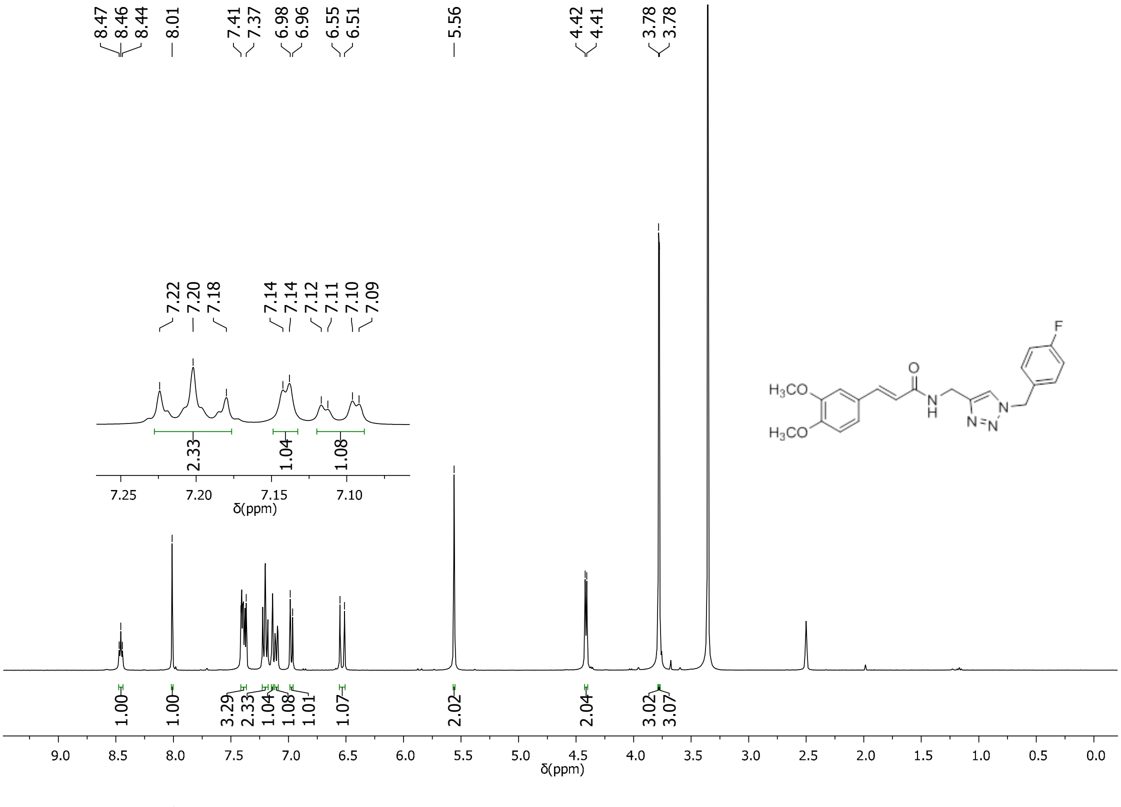

Figure S54 - ^{1}H NMR spectrum (400 MHz, DMSO-d_6) of **18**.

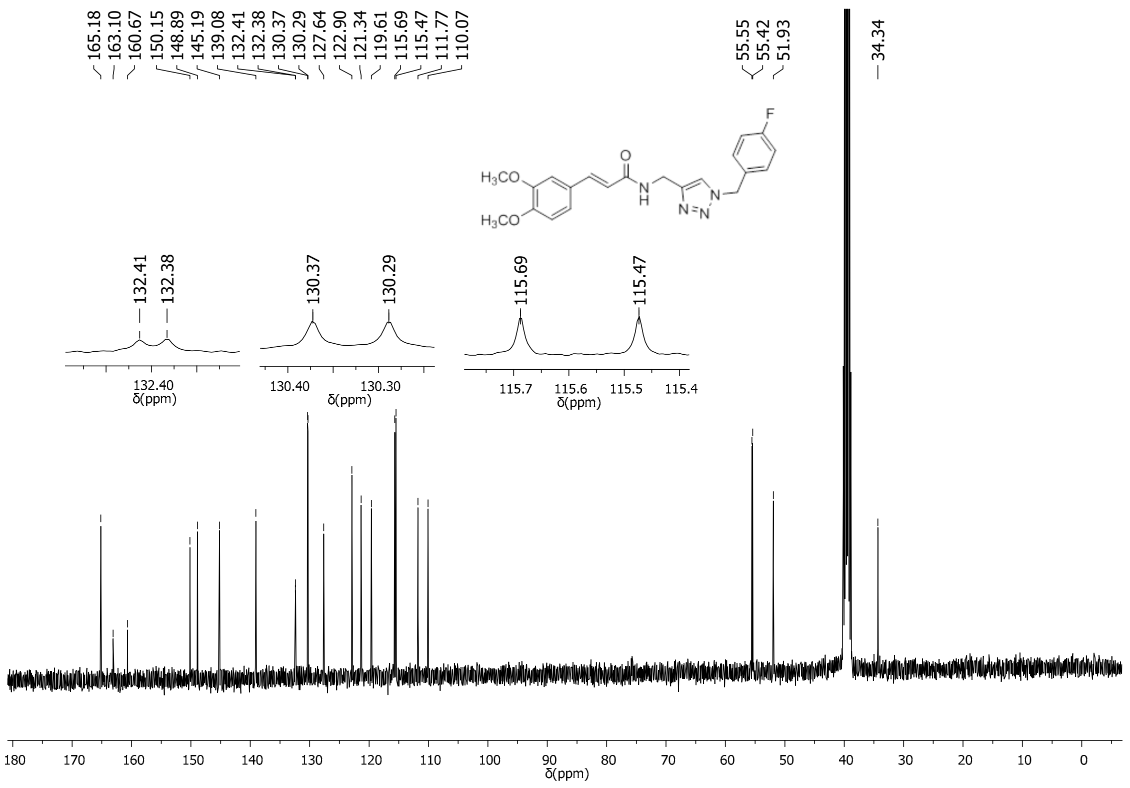

Figure S55 - ^{13}C NMR spectrum (100 MHz, DMSO-d_6) of **18**.

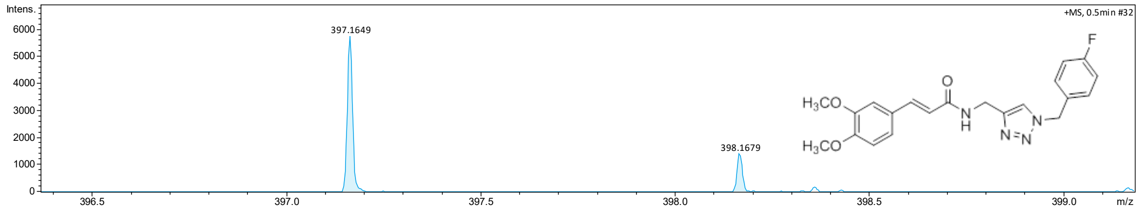

Figure S56 - HRMS spectrum of **18**.

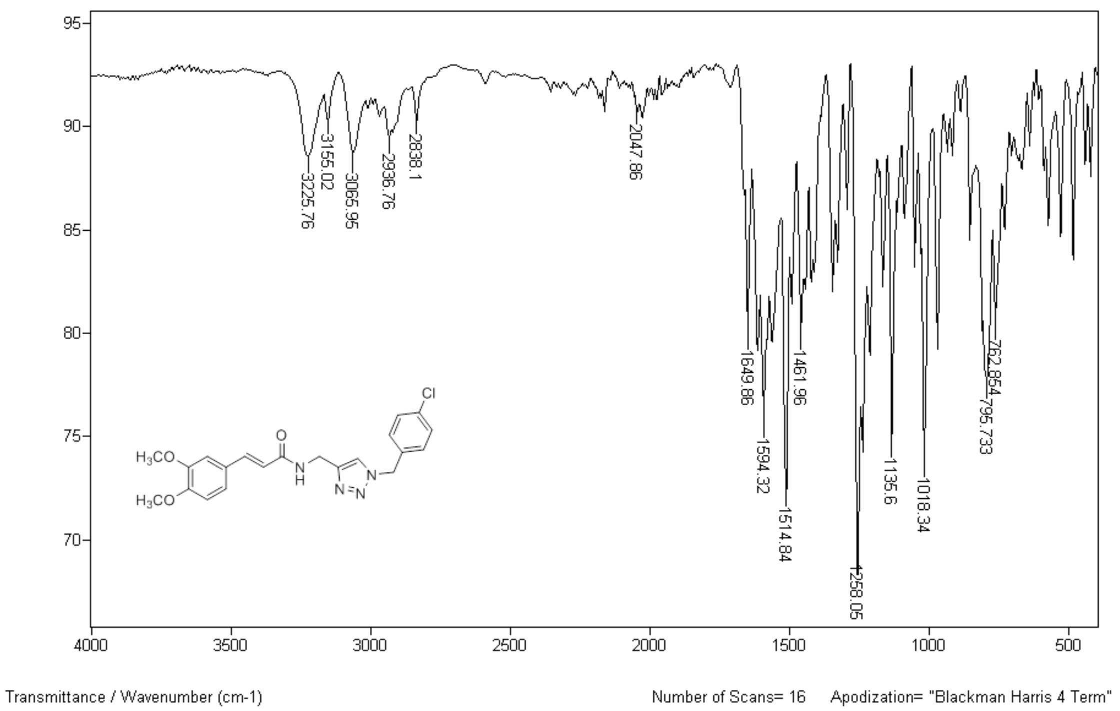

Figure S57 - IR spectrum (ATR) of **19**.

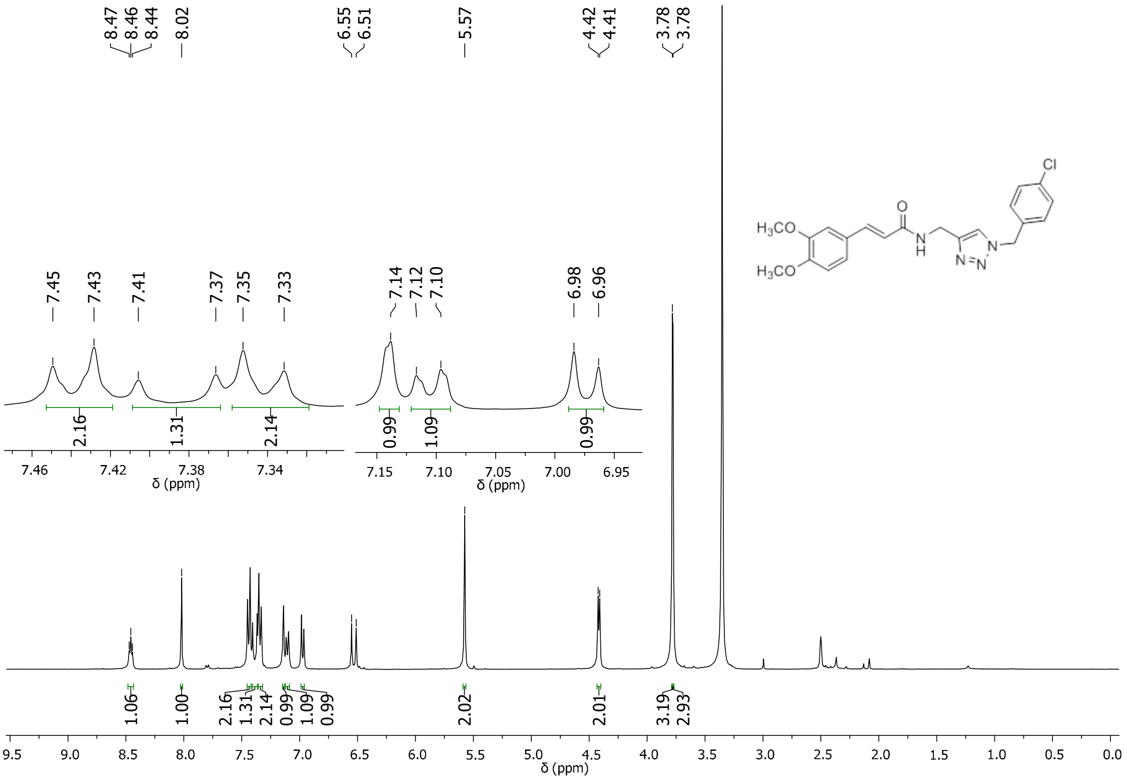

Figure S58 - ^{1}H NMR spectrum (400 MHz, DMSO-d_6) of **19**.

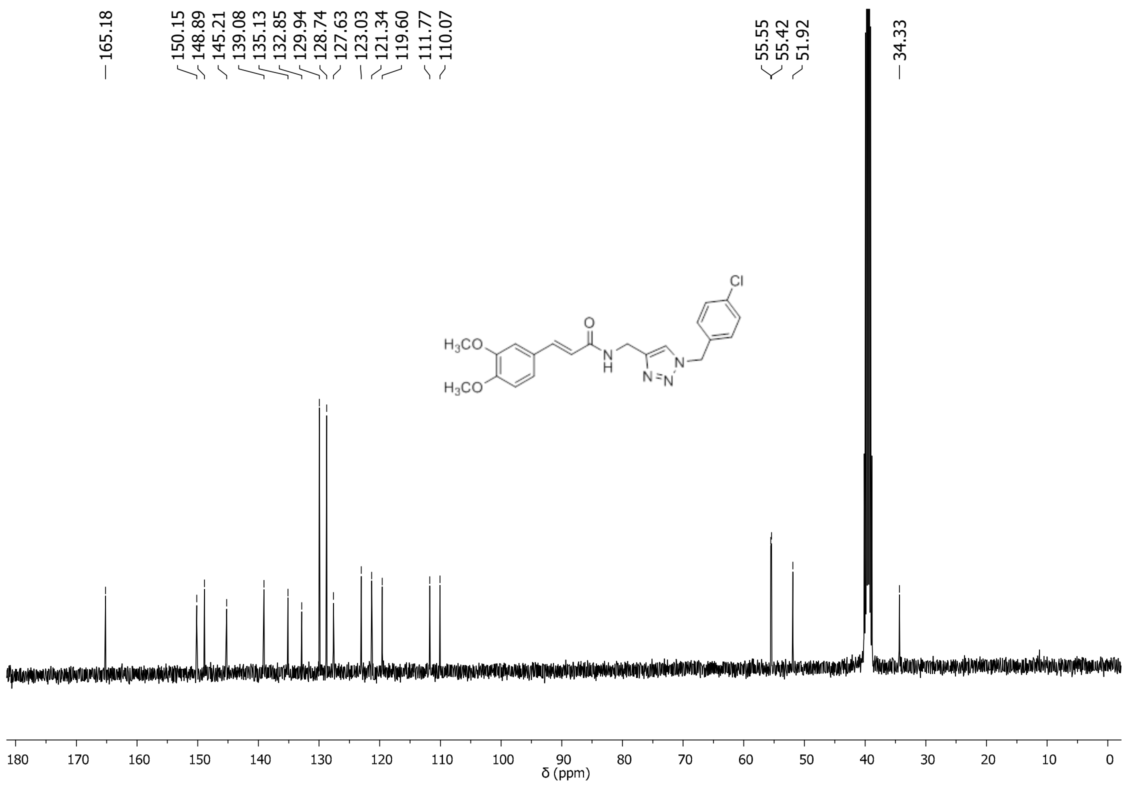

Figure S59 - ^{13}C NMR spectrum (100 MHz, DMSO-d_6) of **19**.

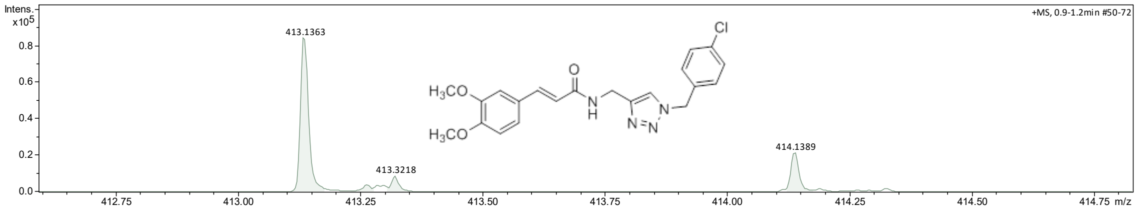

Figure S60 - HRMS spectrum of **19**.

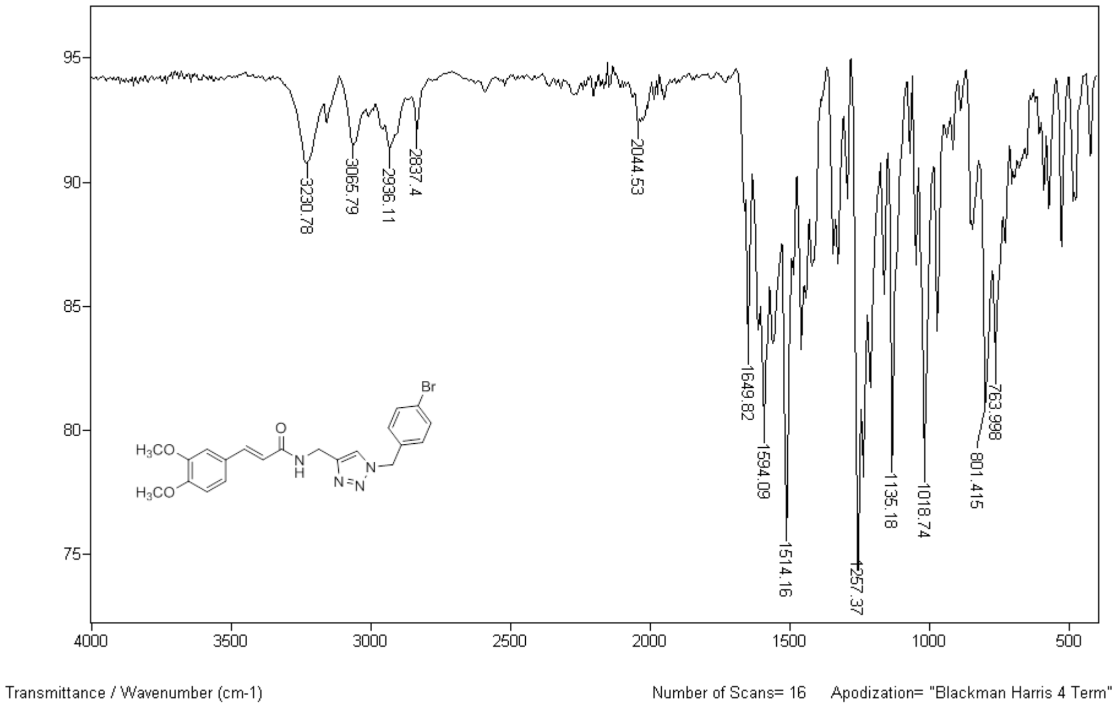

Figure S61 - IR spectrum (ATR) of **20**.

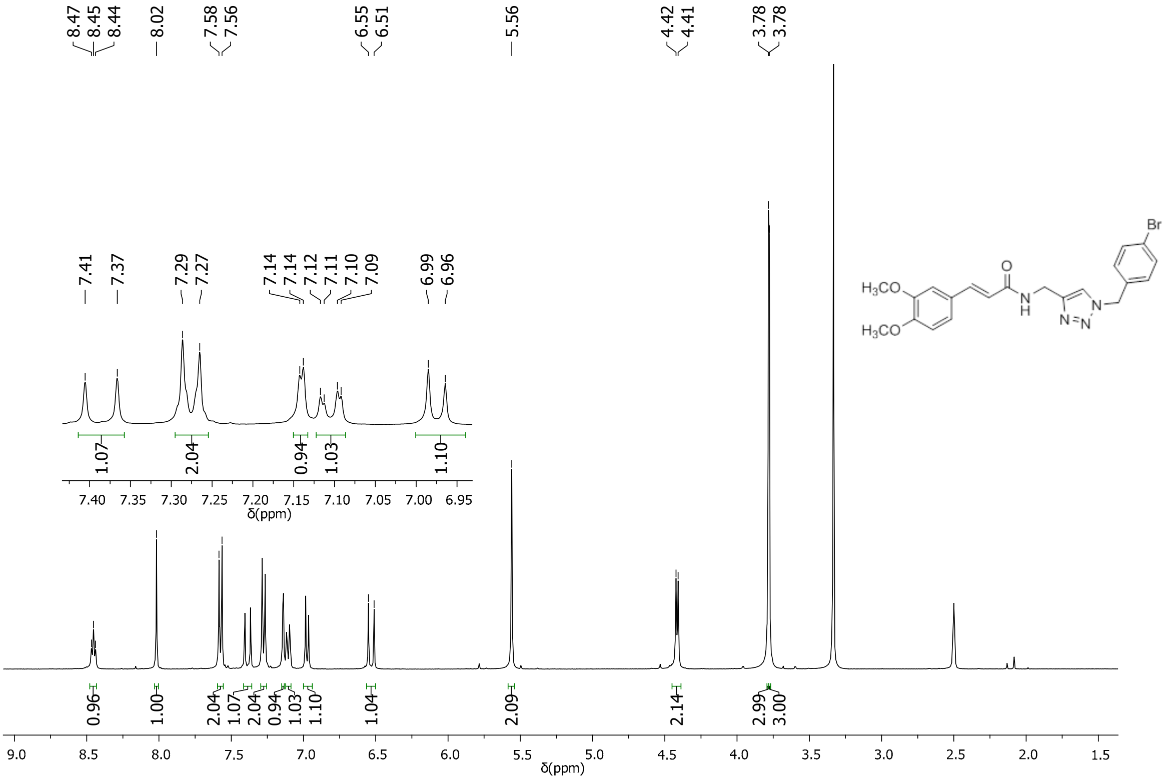

Figure S62 - ^{1}H NMR spectrum (400 MHz, DMSO-d_6) of **20**.

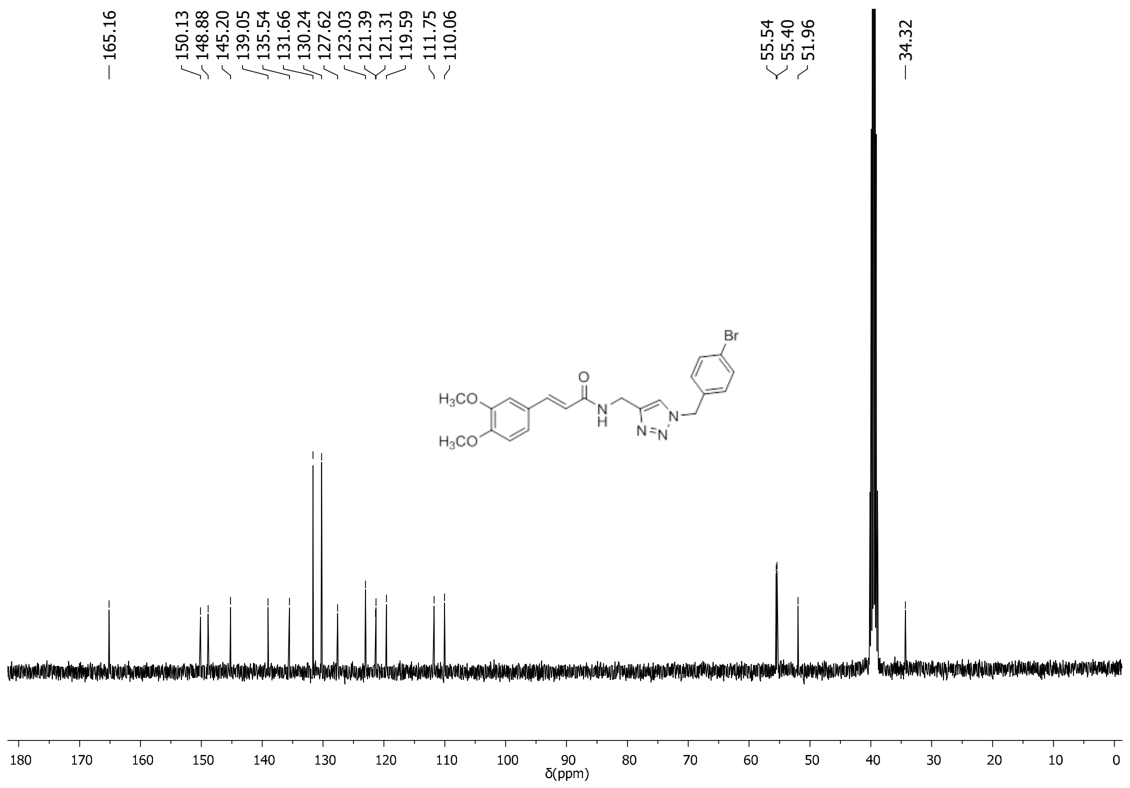

Figure S63 - ^{13}C NMR spectrum (100 MHz, DMSO-d_6) of **20**.

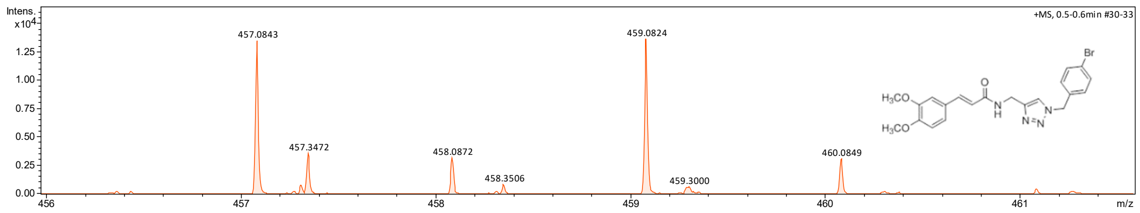

Figure S64 - HRMS spectrum of **20**.

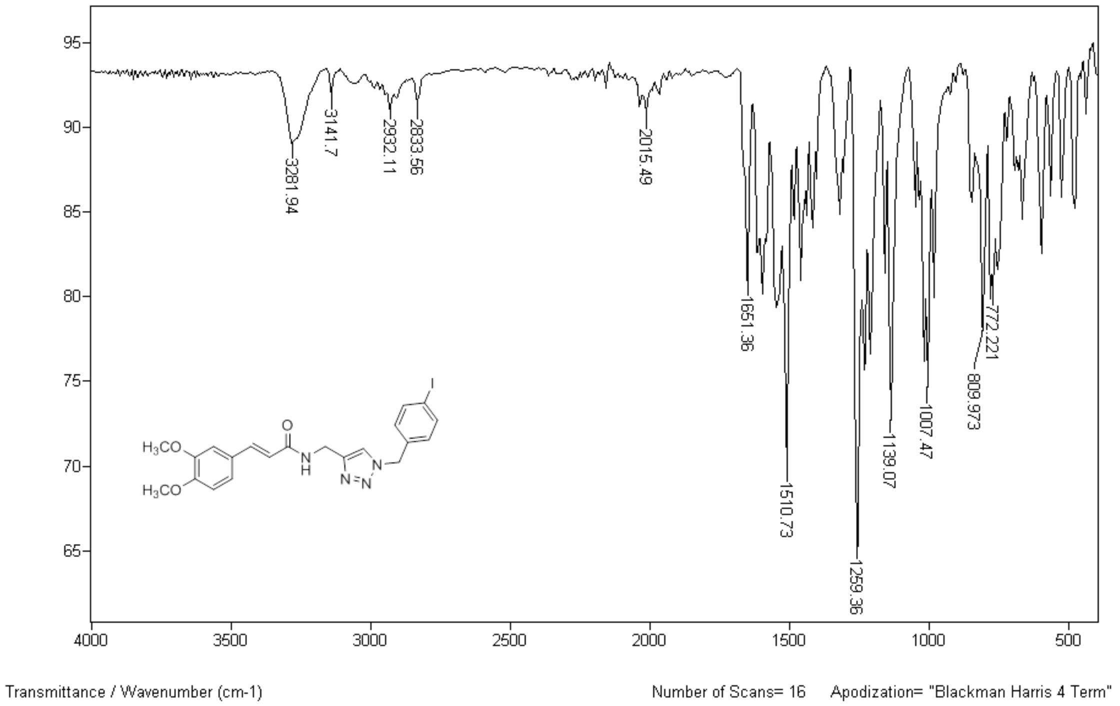

Figure S65 - IR spectrum (ATR) of **21**.

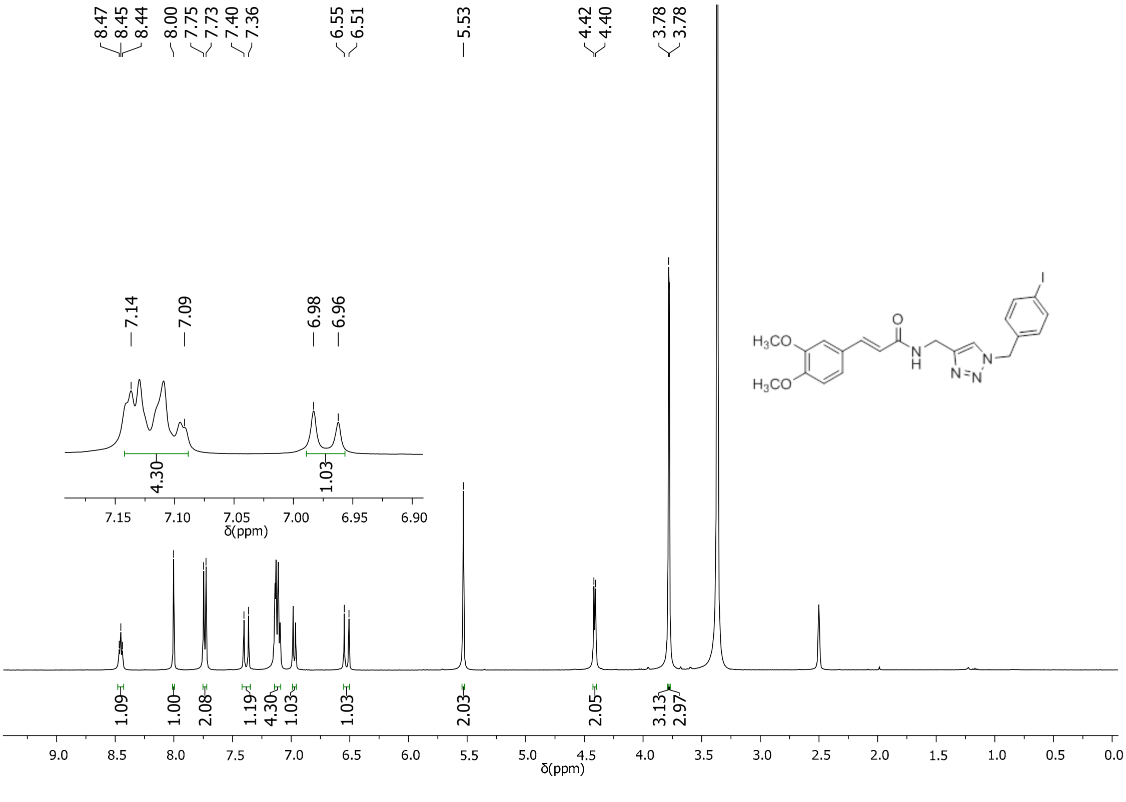

Figure S66 - ^{1}H NMR spectrum (400 MHz, DMSO-d_6) of **21**.

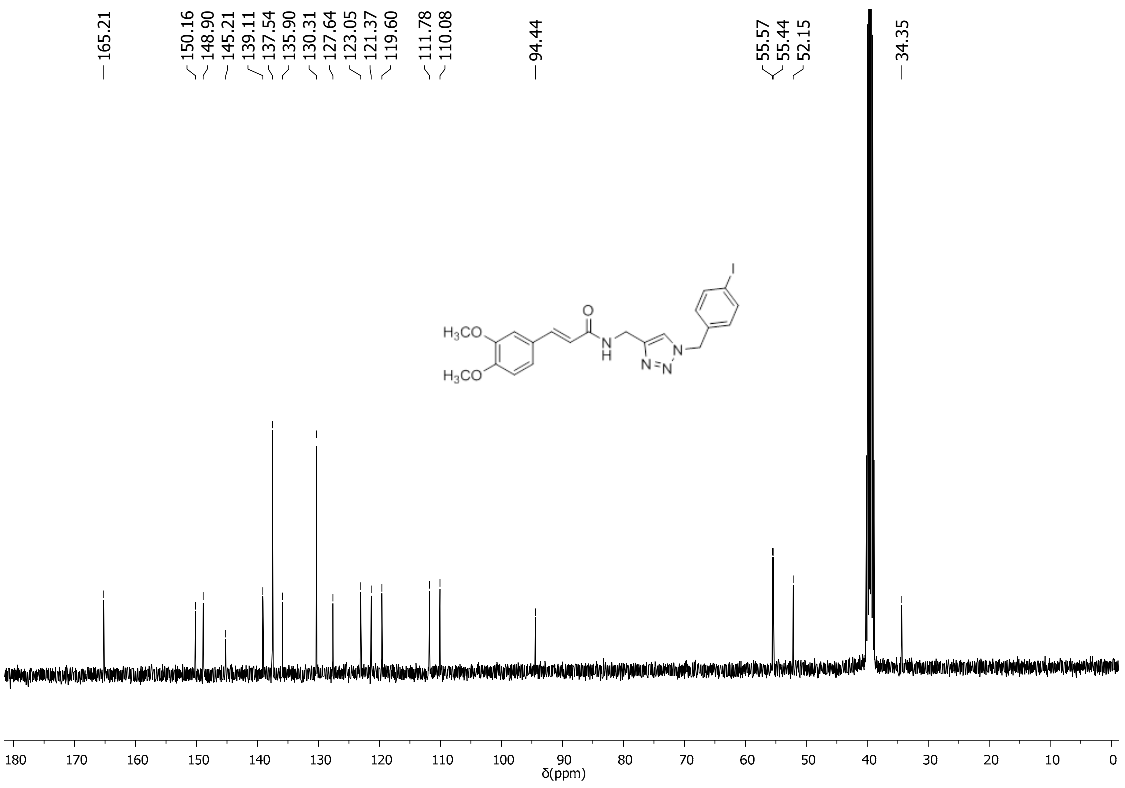

Figure S67 - ^{13}C NMR spectrum (100 MHz, DMSO-d_6) of **21**.

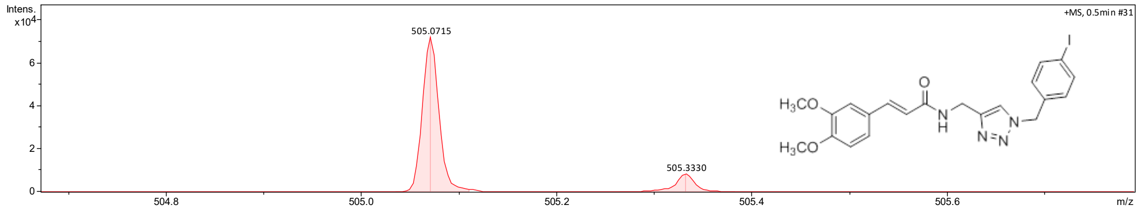

Figure S68 - HRMS spectrum of **21**.

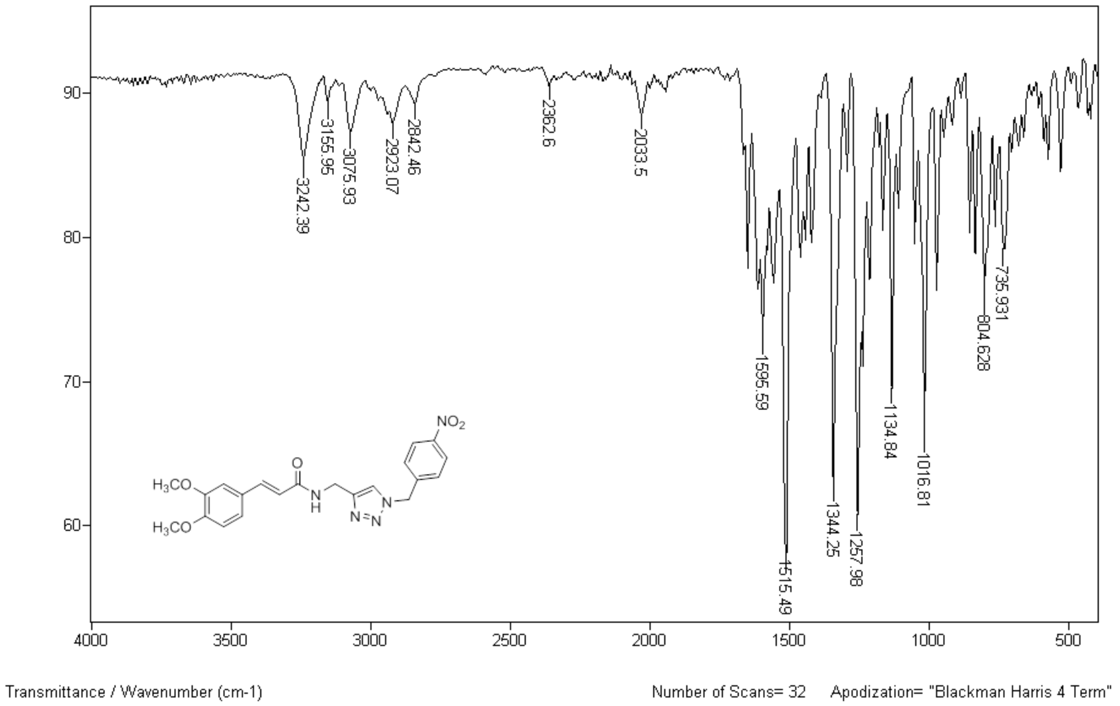

Figure S69 - IR spectrum (ATR) of **22**.

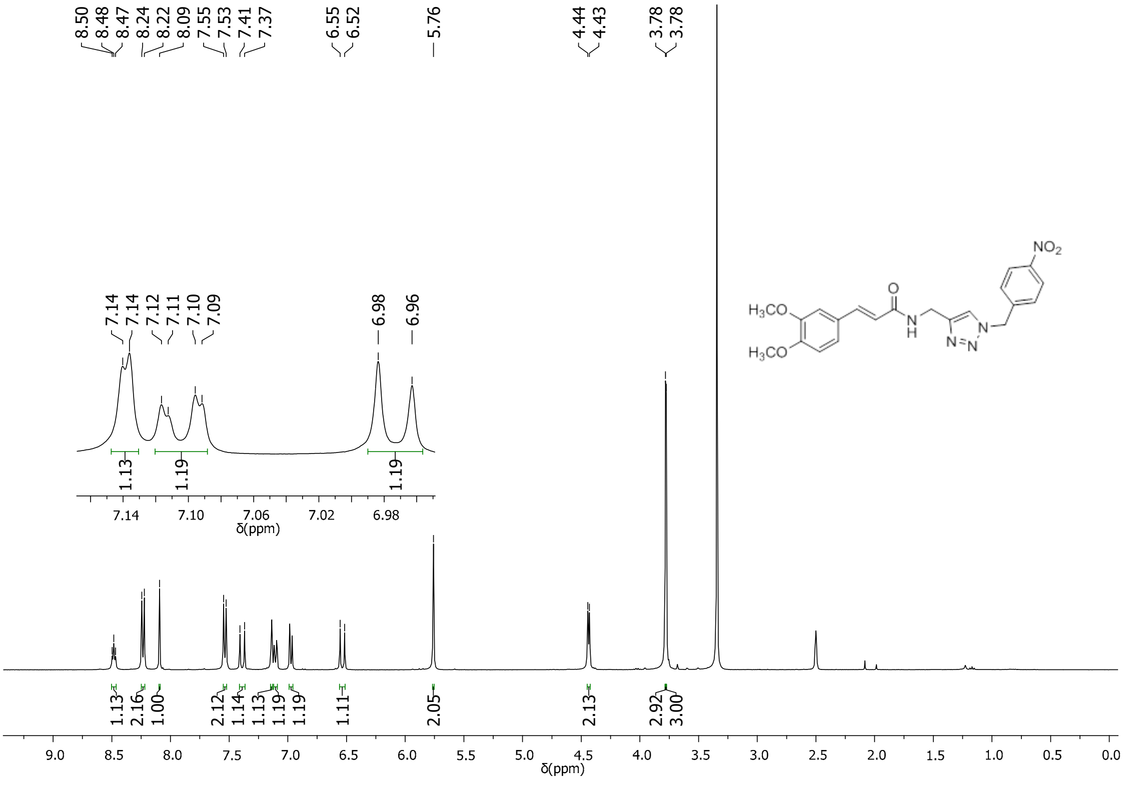

Figure S70 - ^{1}H NMR spectrum (400 MHz, DMSO-d_6) of **22**.

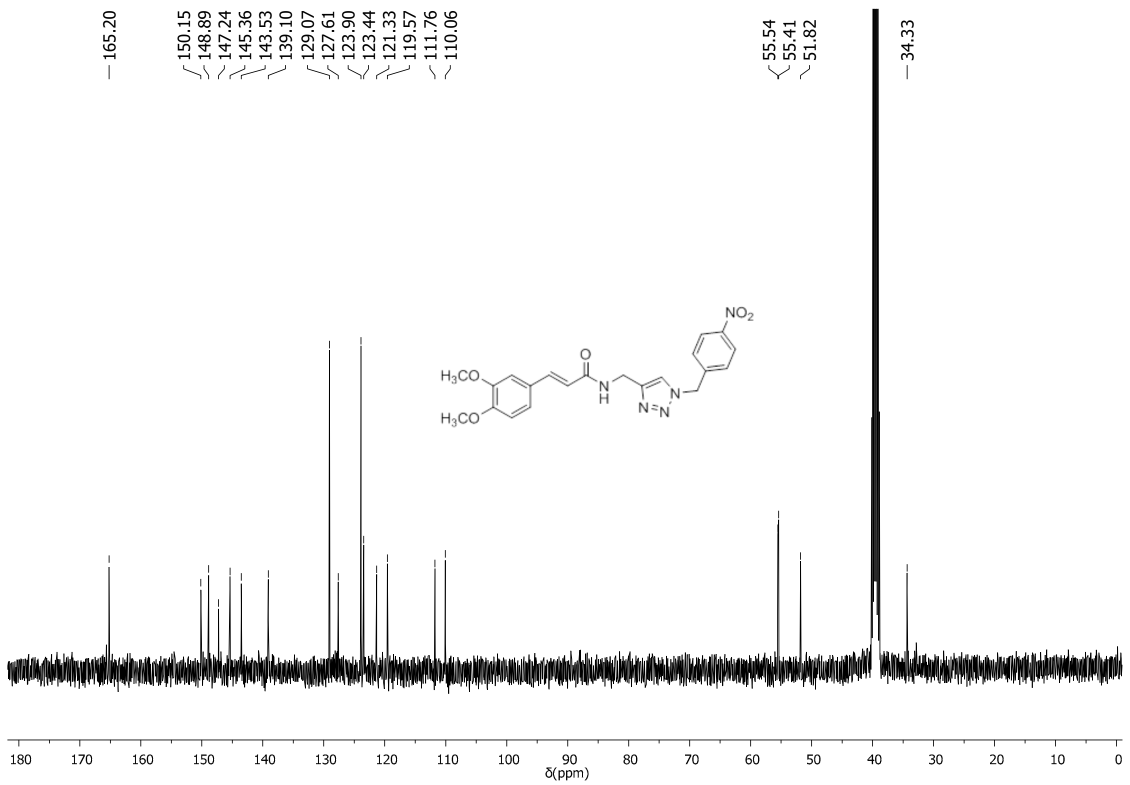

Figure S71 - ^{13}C NMR spectrum (100 MHz, DMSO-d_6) of **22**.

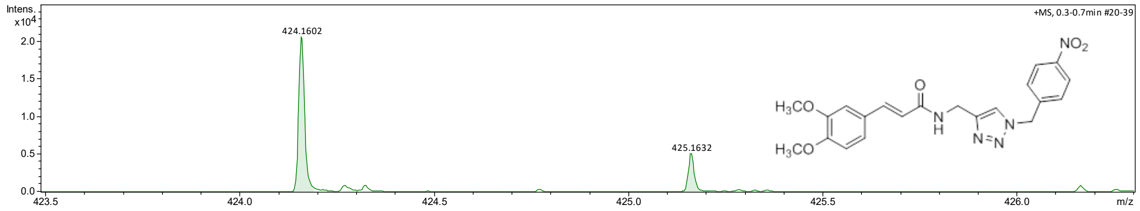

Figure S72 - HRMS spectrum of **22**.

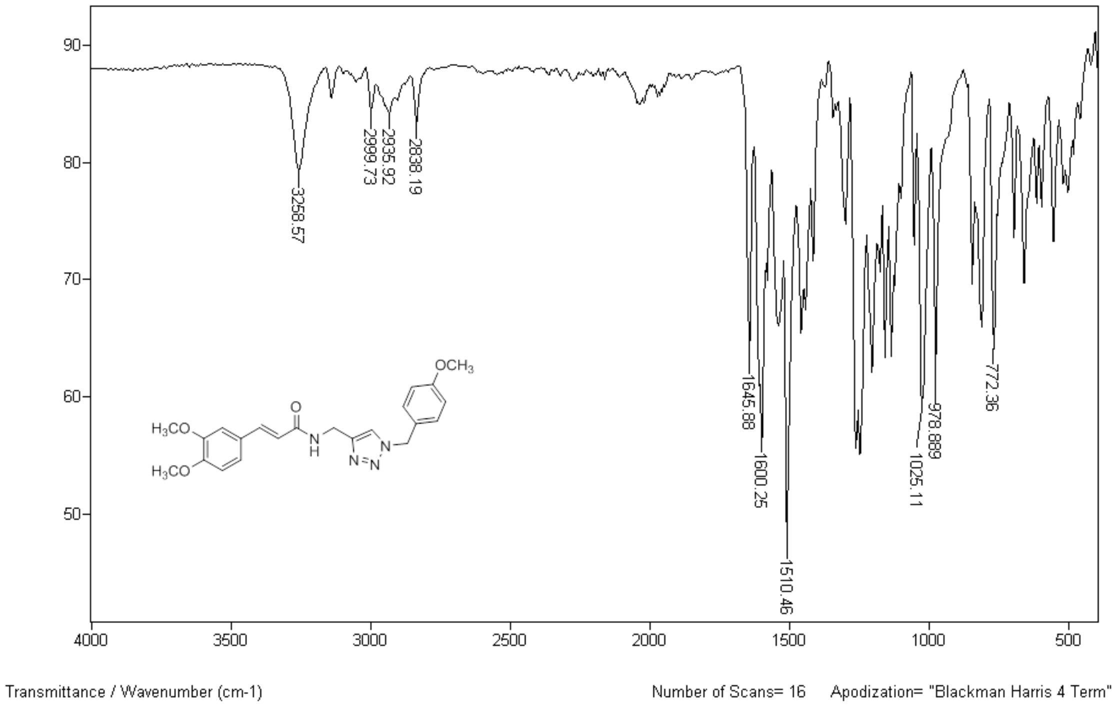

Figure S73 - IR spectrum (ATR) of **23**.

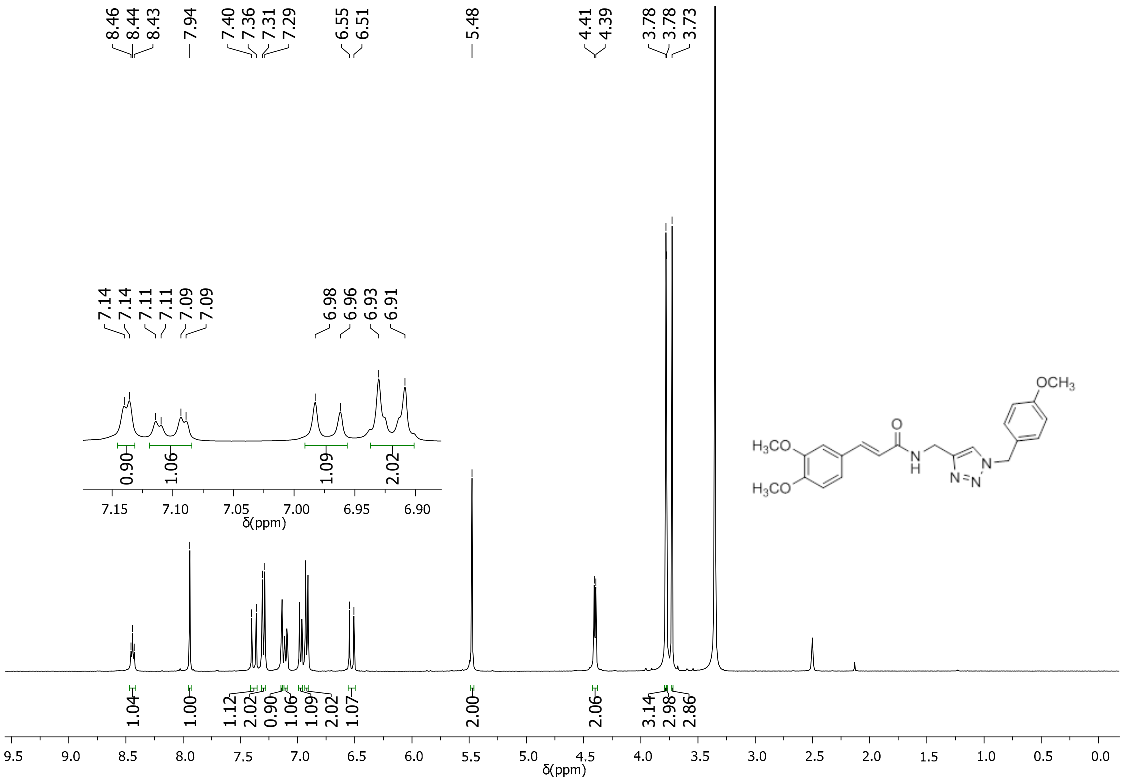

Figure S74 - ^{1}H NMR spectrum (400 MHz, DMSO-d_6) of **23**.

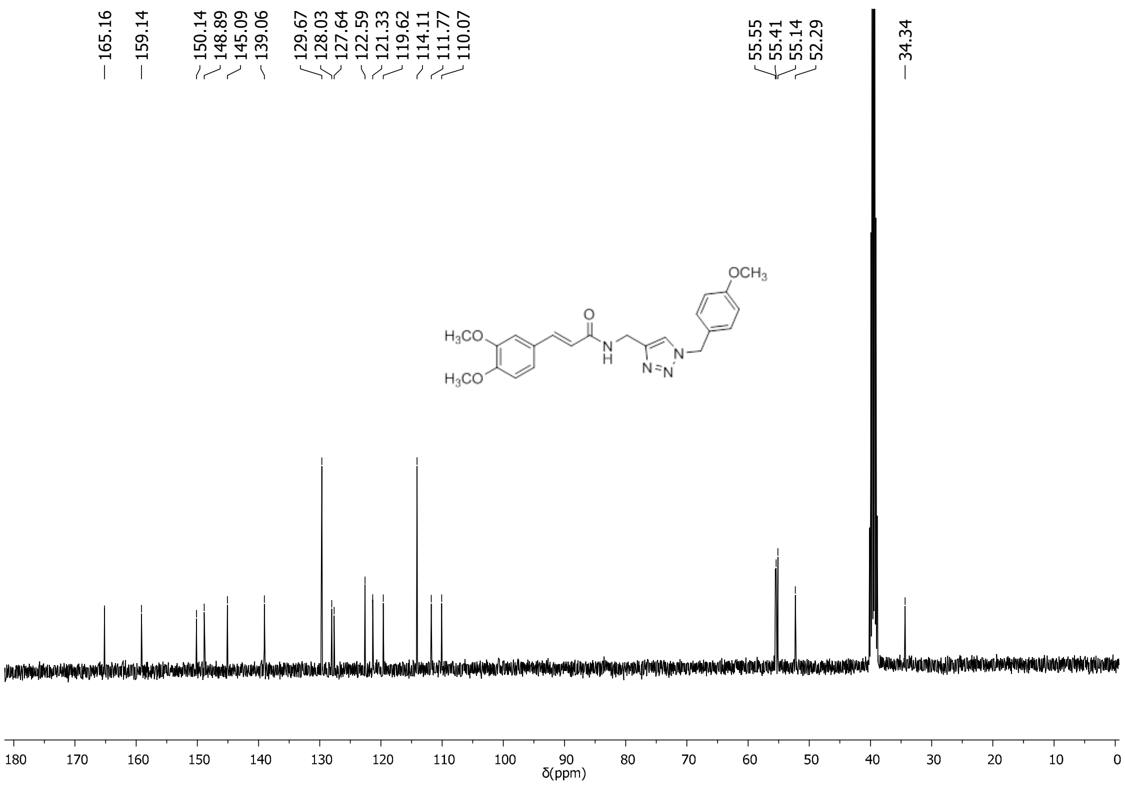

Figure S75 - ^{13}C NMR spectrum (100 MHz, DMSO-d_6) of **23**.

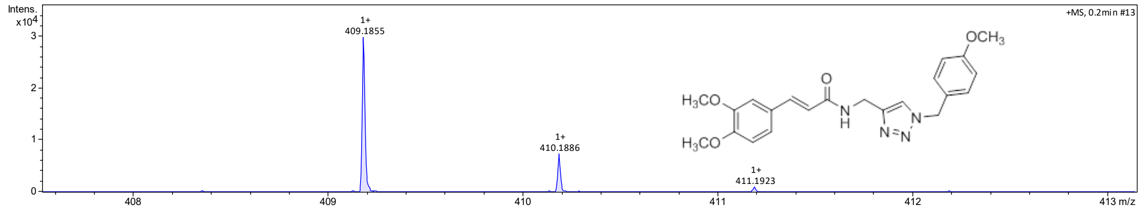

Figure S76 - HRMS spectrum of **23**.

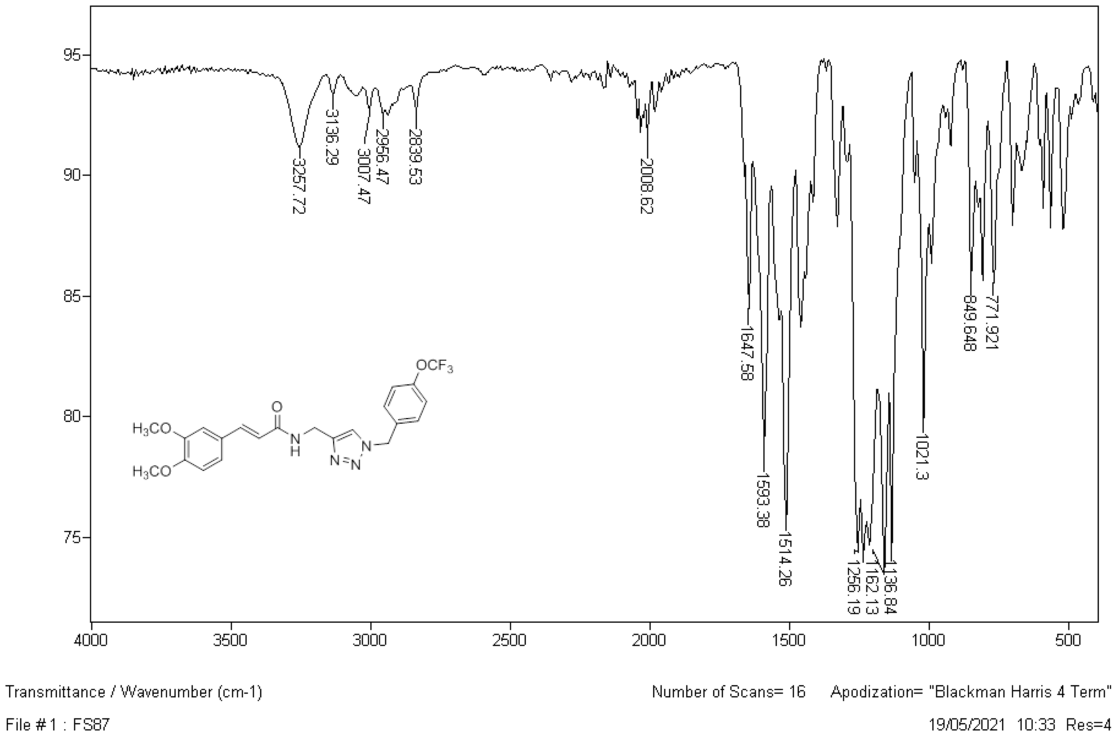

Figure S77 - IR spectrum (ATR) of **24**.

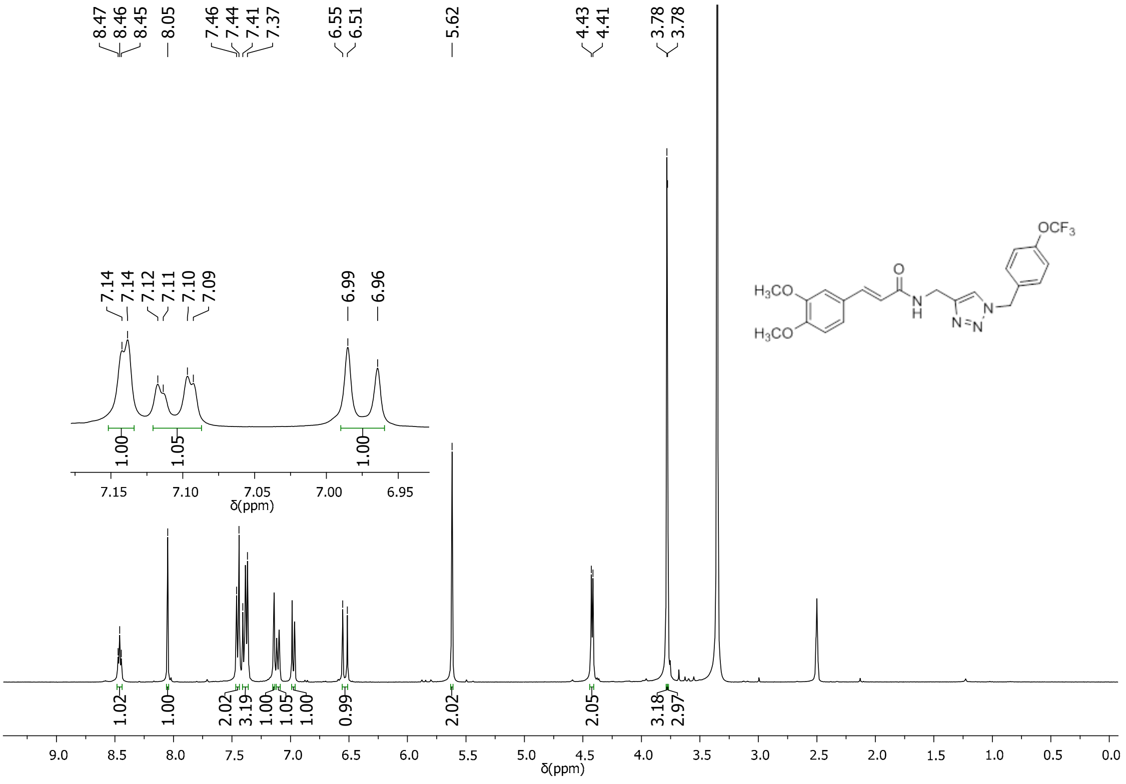

Figure S78 - ^{1}H NMR spectrum (400 MHz, DMSO-d_6) of **24**.

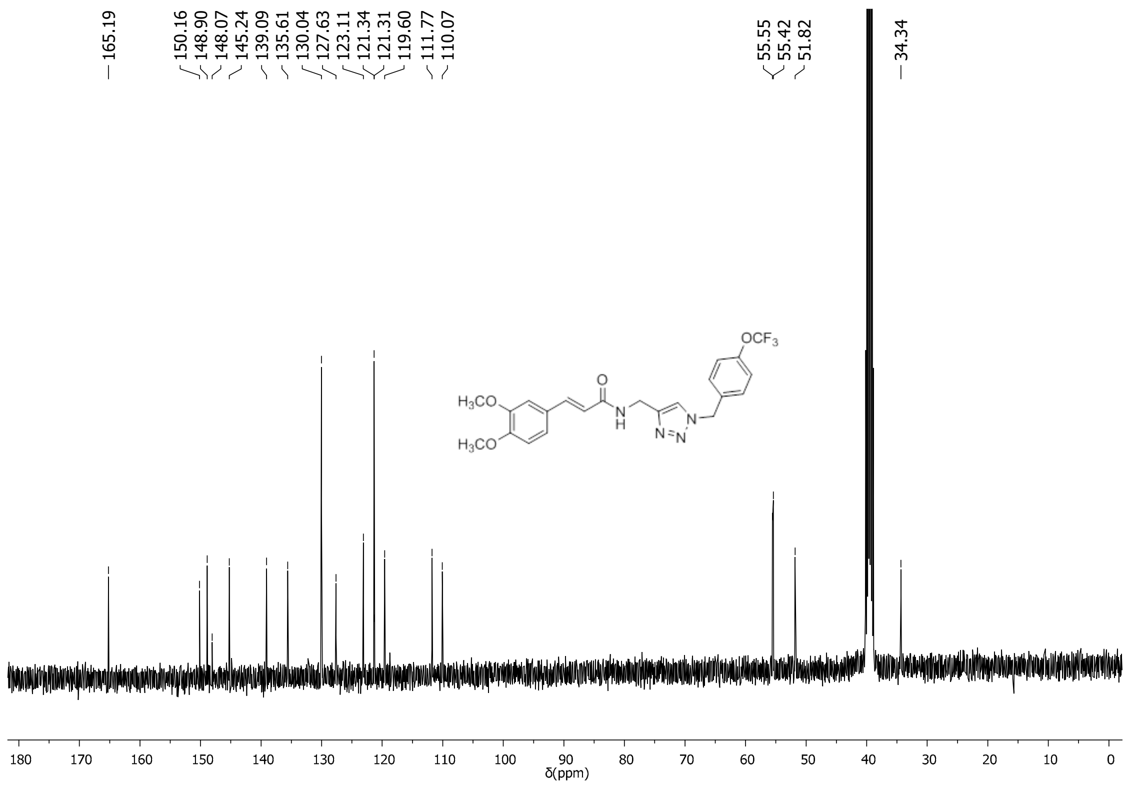

Figure S79 - ^{13}C NMR spectrum (100 MHz, DMSO-d_6) of **24**.

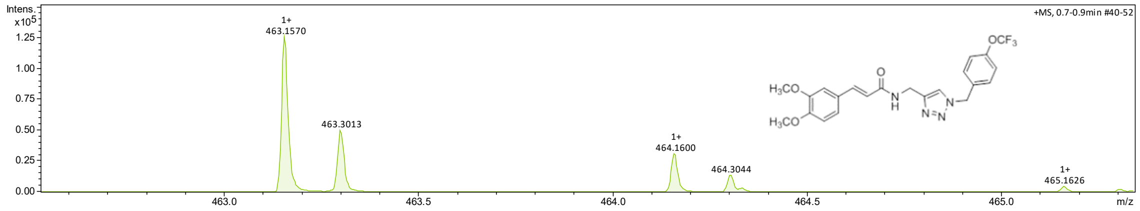

Figure S80 - HRMS spectrum of **24**.

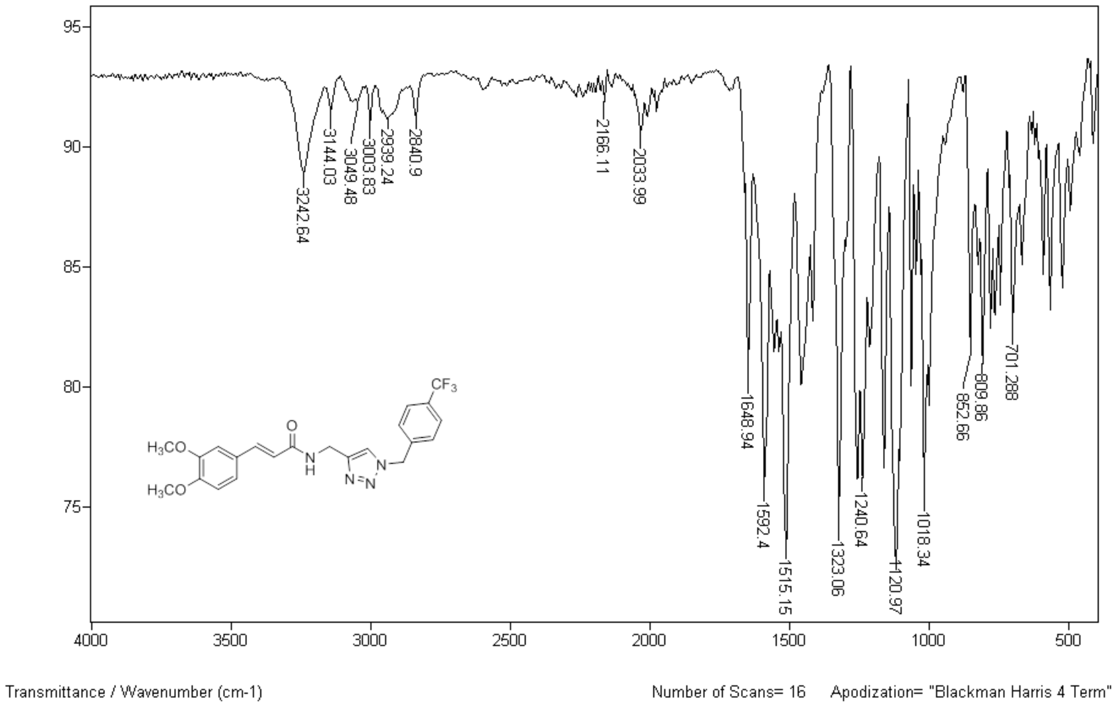

Figure S81 - IR spectrum (ATR) of **25**.

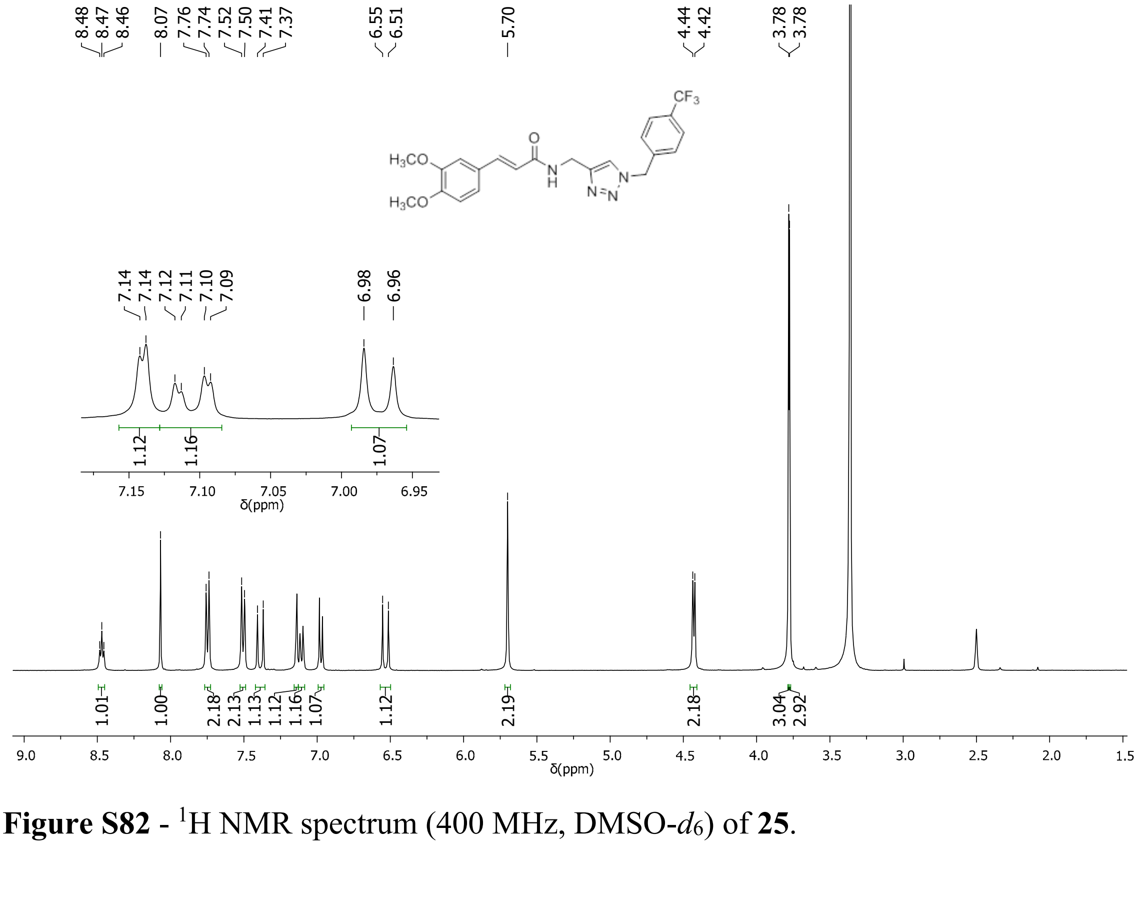

Figure S82 - ^{1}H NMR spectrum (400 MHz, DMSO-d_6) of **25**.

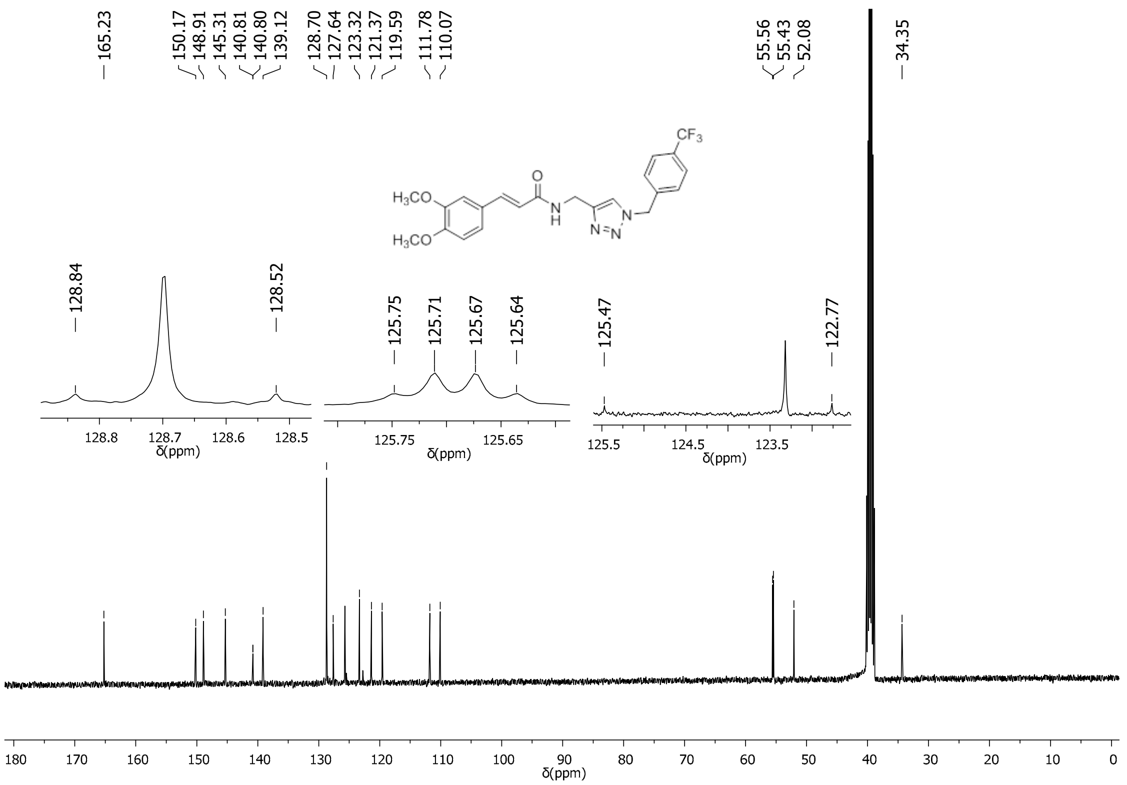

Figure S83 - ^{13}C NMR spectrum (100 MHz, DMSO-d_6) of **25**.

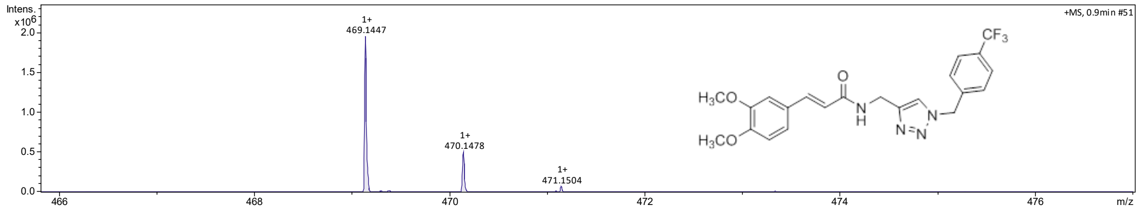

Figure S84 - HRMS spectrum of **25**.

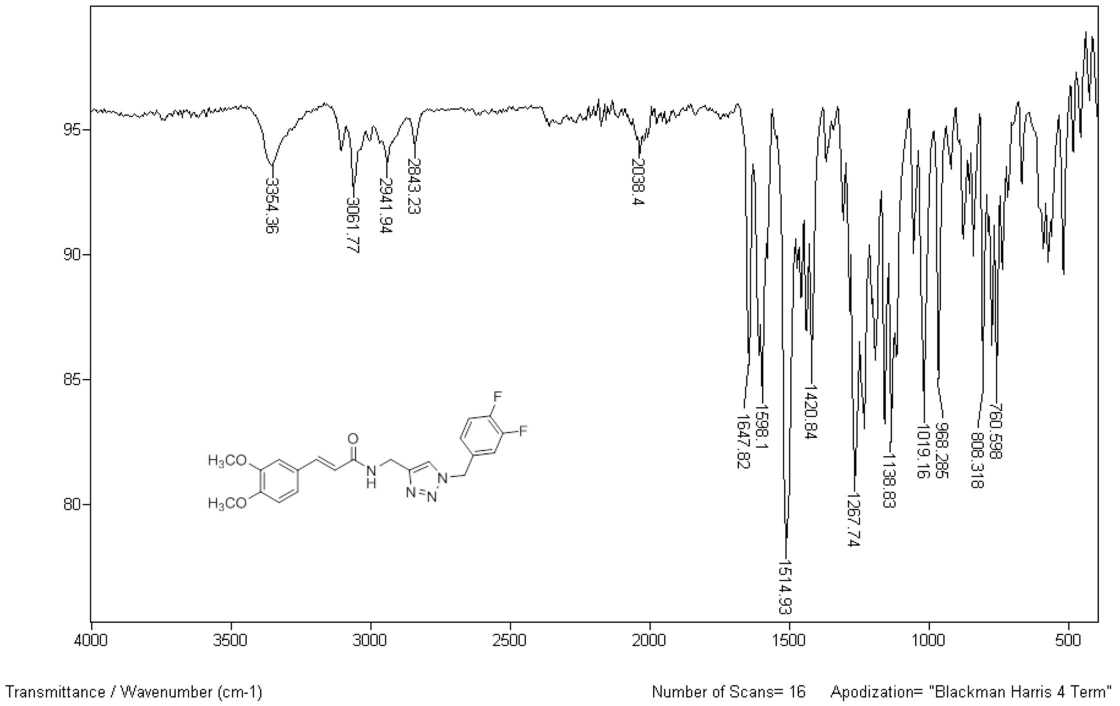

Figure S85 - IR spectrum (ATR) of **26**.

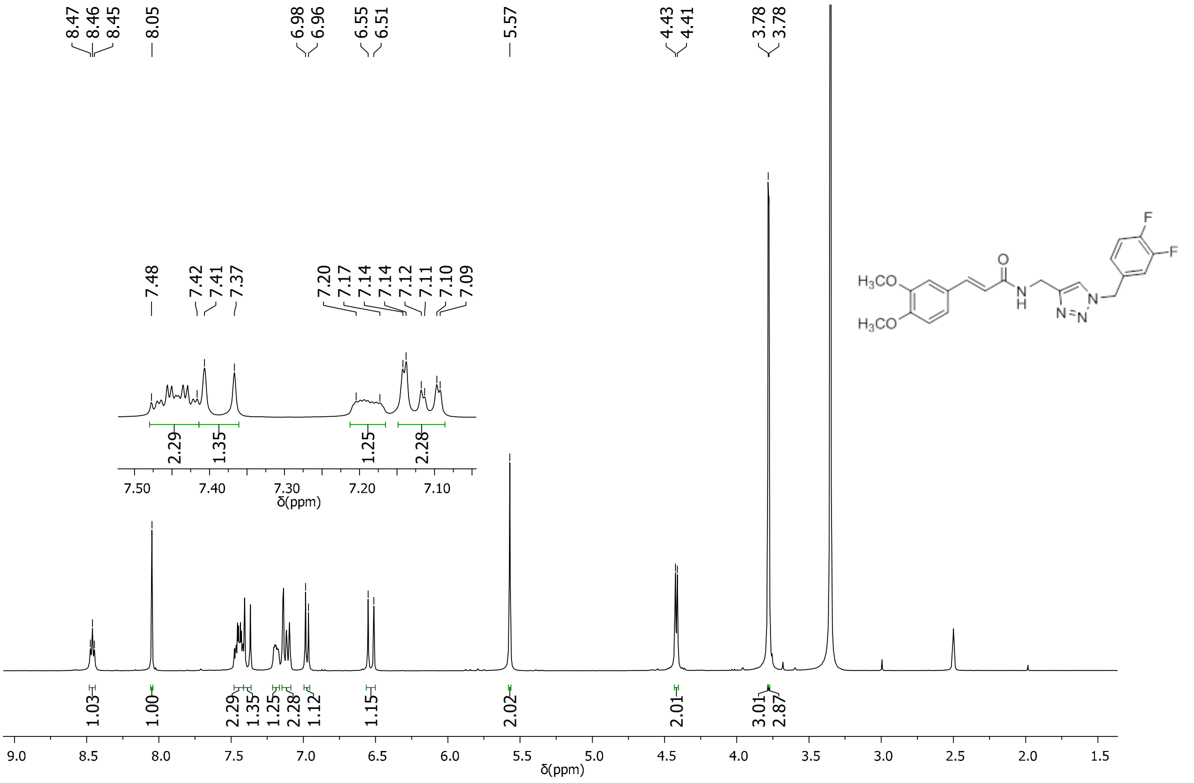

Figure S86 - ^{1}H NMR spectrum (400 MHz, DMSO-d_6) of **26**.

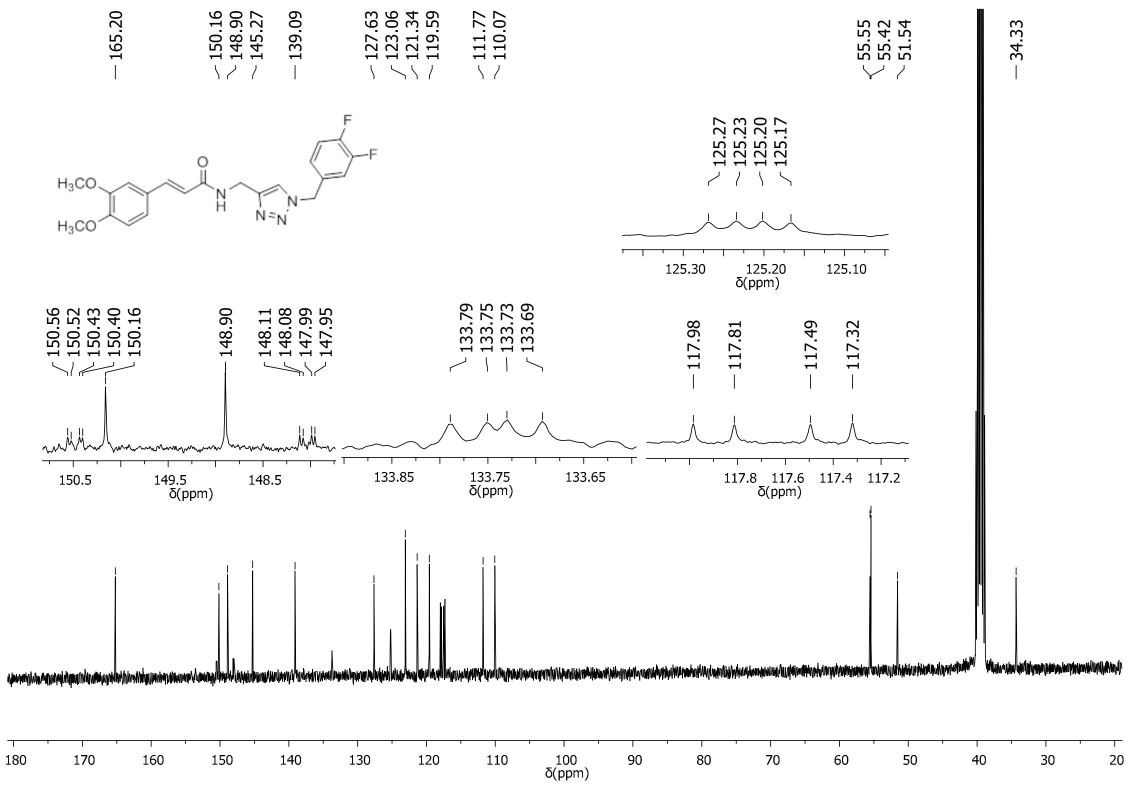

Figure S87 - ^{13}C NMR spectrum (100 MHz, DMSO-d_6) of **26**.

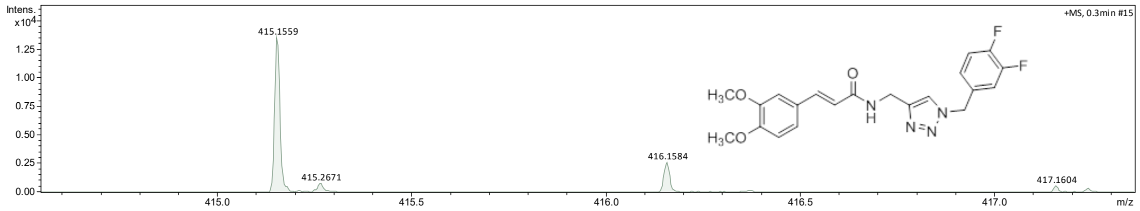

Figure S88 - HRMS spectrum of **26**.

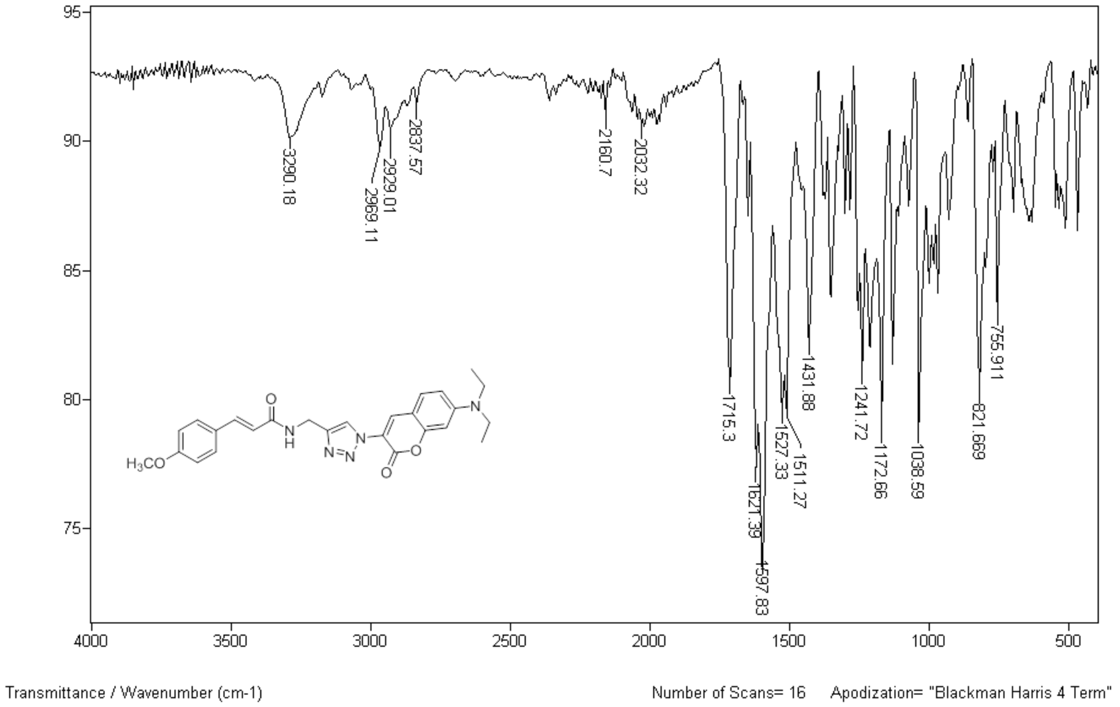

Figure S89 - IR spectrum (ATR) of **27**.

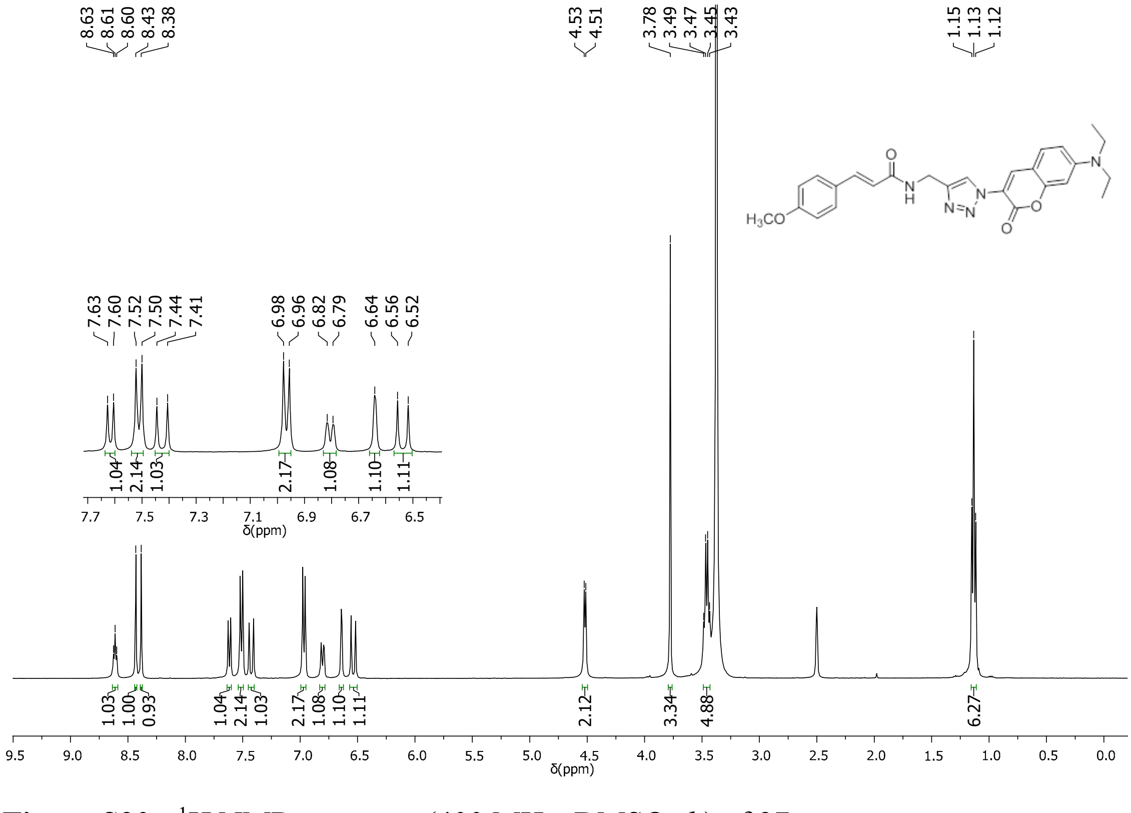

Figure S90 - ^{1}H NMR spectrum (400 MHz, DMSO-d_6) of **27**.

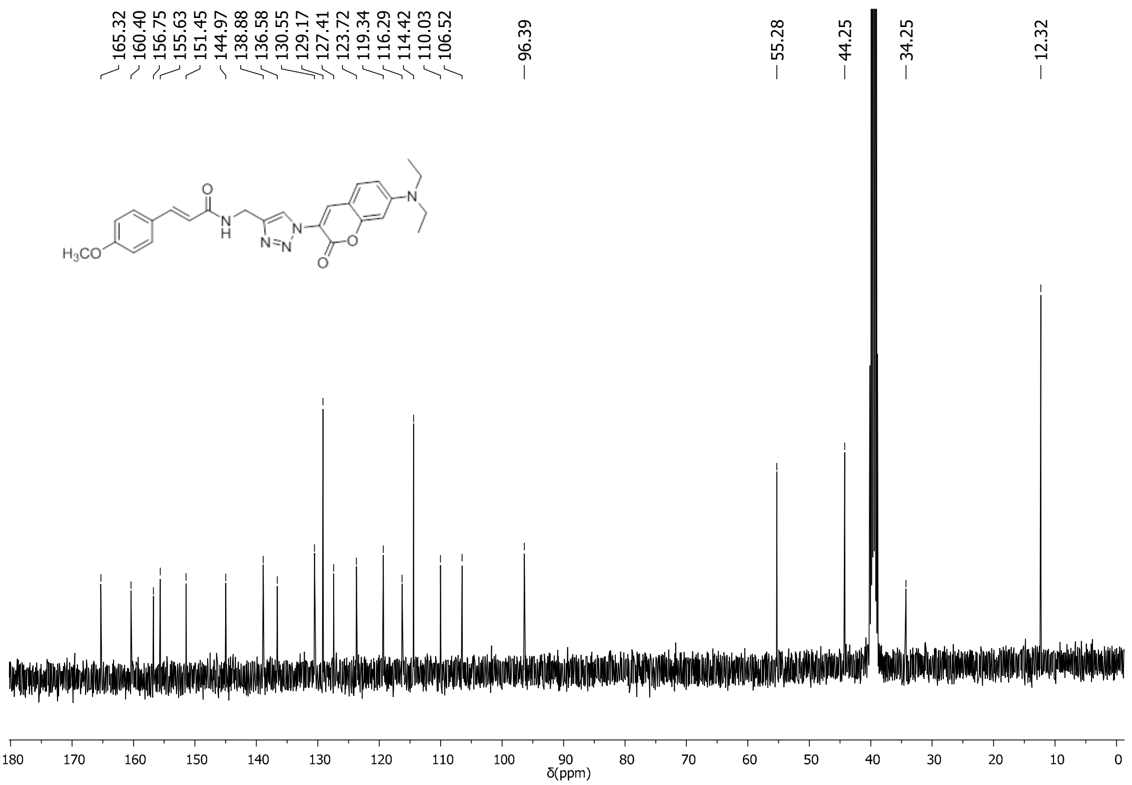

Figure S91 - ^{13}C NMR spectrum (100 MHz, DMSO-d_6) of **27**.

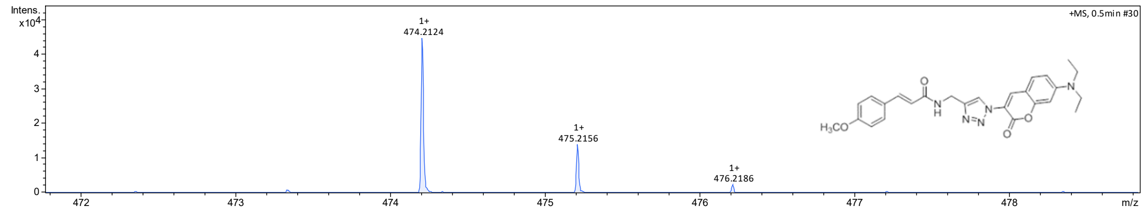

Figure S92 - HRMS spectrum of **27**.

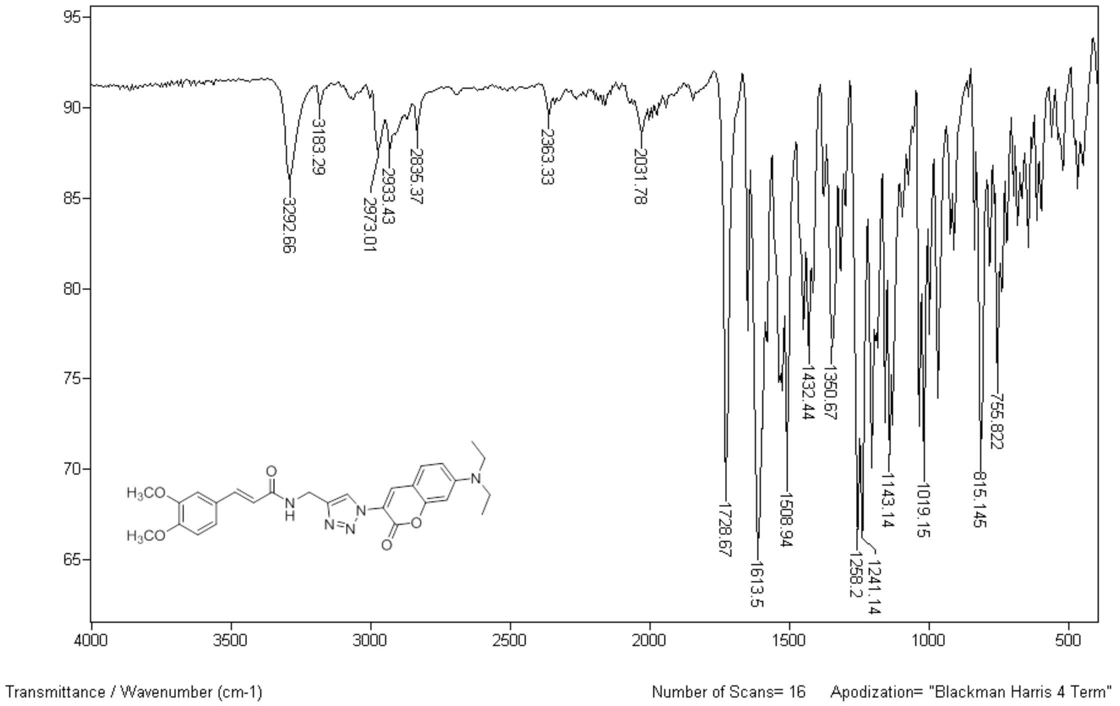

Figure S93 - IR spectrum (ATR) of **28**.

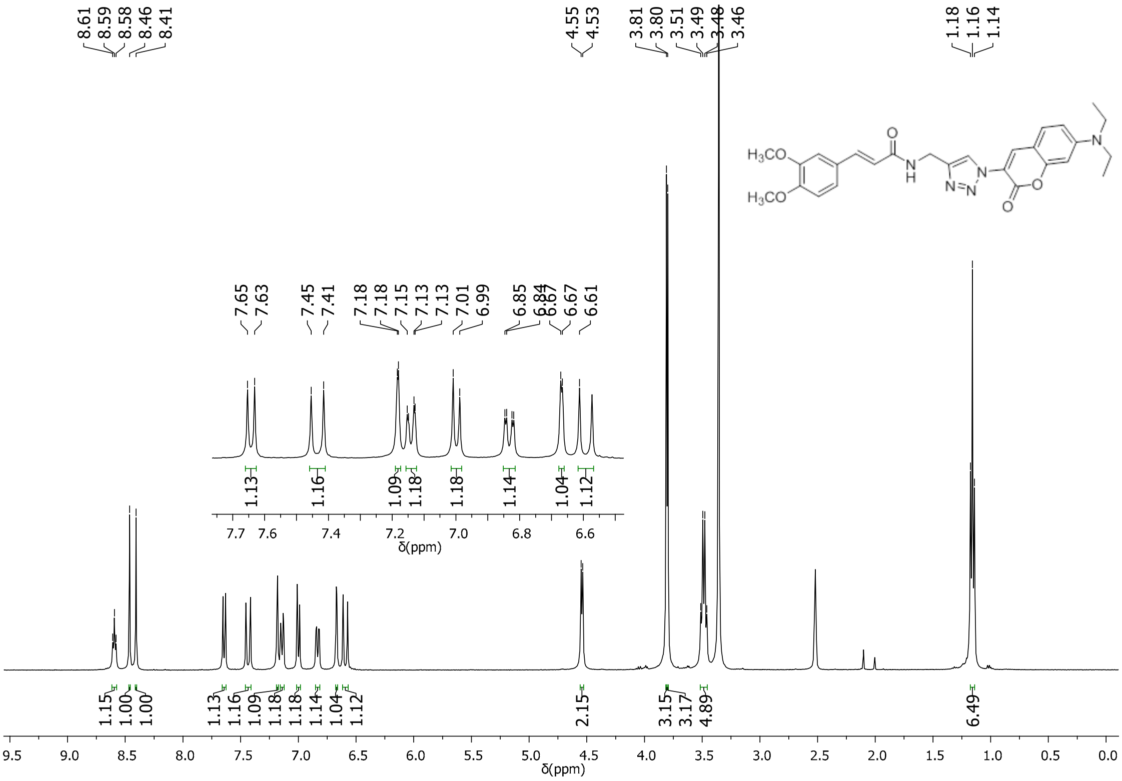

Figure S94 - ^{1}H NMR spectrum (400 MHz, DMSO-d_6) of **28**.

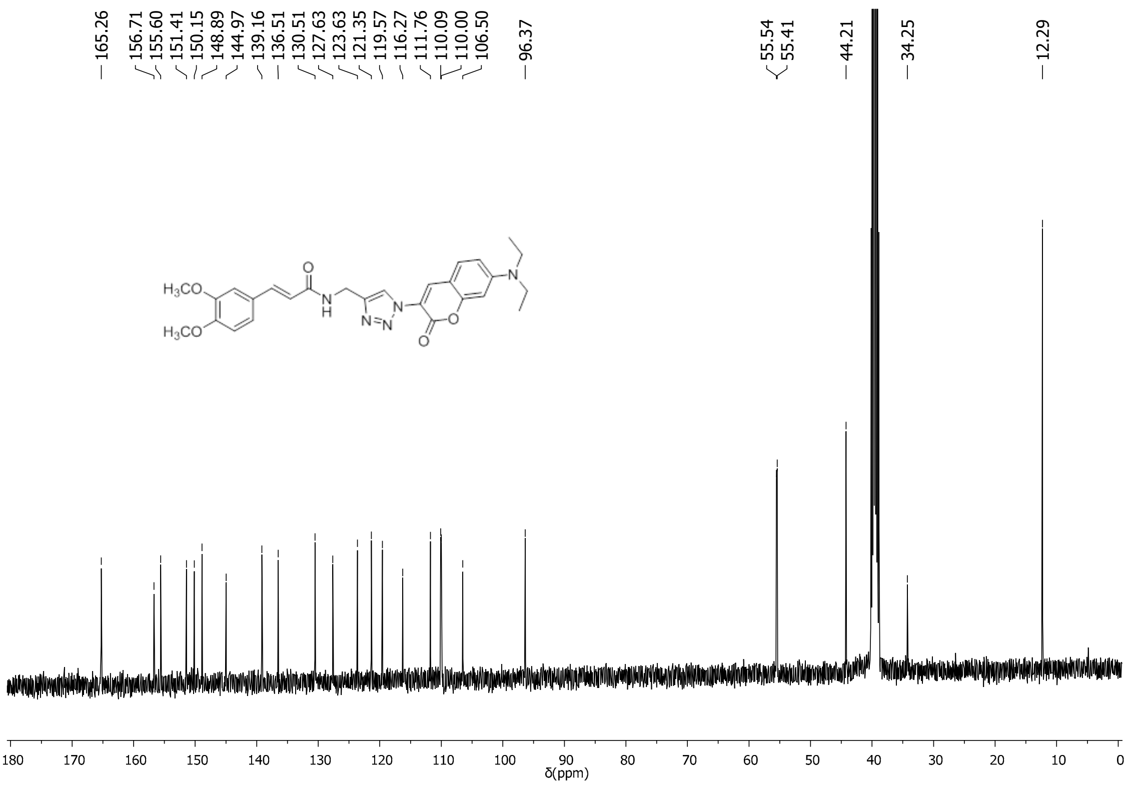

Figure S95 - ^{13}C NMR spectrum (100 MHz, DMSO-*d*$_6$) of **28**.

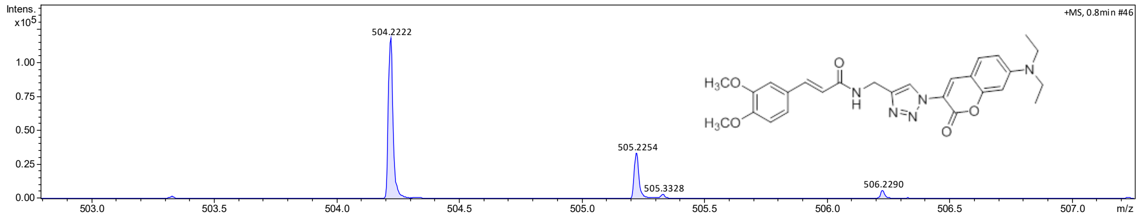

Figure S96 - HRMS spectrum of **28**.
